# Construction of bis-, tris- and tetrahydrazones by addition of azoalkenes to amines and ammonia

**DOI:** 10.3762/bjoc.12.241

**Published:** 2016-11-21

**Authors:** Artem N Semakin, Aleksandr O Kokuev, Yulia V Nelyubina, Alexey Yu Sukhorukov, Petr A Zhmurov, Sema L Ioffe, Vladimir A Tartakovsky

**Affiliations:** 1 Laboratory of functional organic compounds, N.D. Zelinsky Institute of Organic Chemistry Russian Academy of Sciences, Leninsky Prospect, 47, Moscow, 119991, Russia; 2 Moscow Chemical Lyceum 1303, Tamozhenniy proezd, 4, Moscow, 111033, Russia; 3 Laboratory for X-Ray Diffraction Studies, A.N.Nesmeyanov Institute of Organoelement Compounds of Russian Academy of Sciences, Vavilova Str. 28, Moscow, 119991, Russia

**Keywords:** azoalkenes, α-halogen hydrazones, heterocage compounds, hydrazone ligands, Michael addition

## Abstract

Exhaustive Michael-type alkylations of amines and ammonia with azoalkenes (generated from α-halohydrazones) were demonstrated as an efficient approach to poly(hydrazonomethyl)amines – a novel class of polynitrogen ligands. An intramolecular cyclotrimerization of C=N bonds in tris(hydrazonomethyl)amine to the respective 1,4,6,10-tetraazaadamantane derivative was demonstrated.

## Introduction

Hydrazones are extensively used as key structural units in the design of various functional molecular and supramolecular architectures [1–17]. The hydrazone group is a chemically stable, easily assembled motif with prospective coordination properties, which can be tuned by substitution at the carbon and nitrogen atoms. Furthermore, a reversible *E*/*Z*-isomerism of the C=N bond allows controllable modulation of the molecular geometry, for example through coordination with metal cations, hydrogen bond formation or irradiation. These unique structural features of the hydrazone fragment have been successfully exploited in the design of various molecular switches, fluorophores and machines.

**Figure 1:**
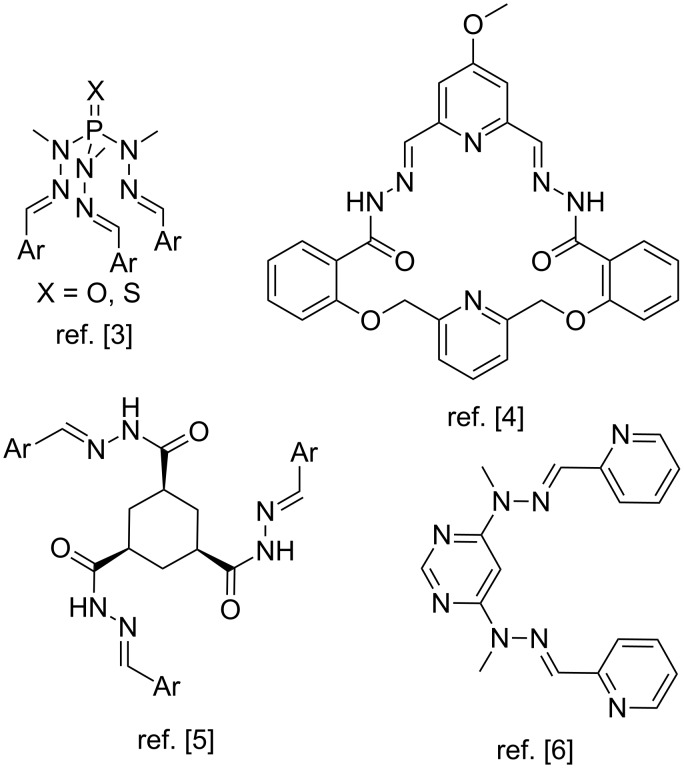
Selected examples of polyhydrazones.

Bis- and polyhydrazones exhibit a rich coordination chemistry owing to a variety of binding modes and are widely employed as ligands in metal-organic assemblies, sensors and catalytic systems [1–17]. More complex structures containing several hydrazone groups integrated with functional fragments upon coordination with metals can undergo significant changes in molecular shape and aggregation state that can be used in the design of smart adaptive materials [1–2]. Some important bis- and trishydrazone ligands used in catalysis, coordination and supramolecular chemistry are shown in Figure 1.

**Scheme 1:**
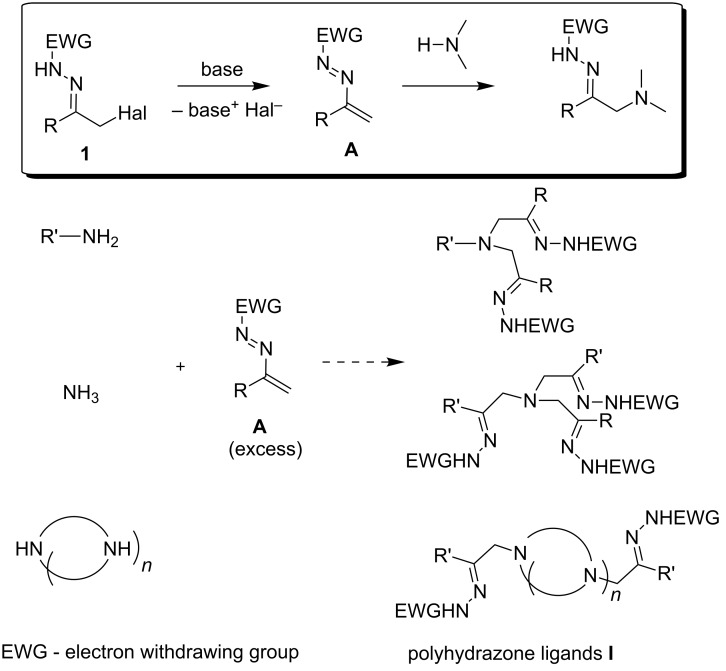
Proposed approach to the synthesis of **I**.

Despite complex and sophisticated polyhydrazone ligands have been designed in the last decade, more structurally simple poly(hydrazonomethyl)amines of type **I** (Scheme 1), which are analogs of well-known poly(oximinomethyl)amine and poly(azolylmethyl)amine ligands [18–34], have not been prepared so far. In the present work, we focused on the development of a general approach to tertiary amines and polyamines bearing several hydrazonomethyl arms at the nitrogen atom(s). To achieve this goal, we suggested a straightforward methodology based on multiple Michael-type additions of azoalkenes **A** (generated from α-halogen azacarbonyl precursor **1** [35–39]) to amines or ammonia (Scheme 1).

Though the chemistry and synthetic potential azoalkenes **A** have been a subject of considerable interest in the recent years [38–39], their reactivity with amines is poorly explored. It has been demonstrated that amines react with azoalkenes **A** forming α-aminohydrazones (Scheme 1) [35–49], however, addition of several azoalkene molecules to amines is virtually unknown. To our knowledge, there is only one report on the formation of bishydrazones as undesirable products in reactions of some primary amines with *N*-tosylhydrazone of *o*-bromophenacyl bromide [50]. We suppose that extension of the scope of azoalkene–amine coupling to ammonia, primary amines and polyamines would open an easy access to various polyhydrazones of type **I**. Therefore, a comprehensive study on the interaction of various amines with α-halogen-substituted hydrazones **1** with amines and ammonia was undertaken.

## Results and Discussion

### Synthesis of α-halogen-substituted hydrazones **1**

**Scheme 2:**
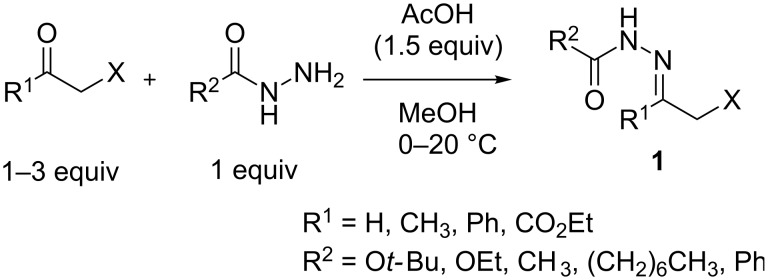
Synthesis of α-halogen-substituted hydrazones **1** from α-halocarbonyl compounds and acylhydrazines or carbazates.

Initially, α-halogen-substituted hydrazones **1** were prepared from the corresponding carbonyl compounds and acylhydrazines or carbazates to study the reaction with amines (Scheme 2, for details see Supporting Information File 1). Acetic acid was added as catalyst and for suppression of the side reaction of the formed α-halogen hydrazones with starting hydrazide [51]. The presence of acetic acid and mild reaction conditions (0 °C) was essential for the synthesis of hydrazones **1c** and **1d** (R^1^ = CH_3_, R^2^ = CH_3_ or (CH_2_)_6_CH_3_), probably because of the their enhanced NH-acidity.

### Reaction of α-halogen hydrazones **1** with benzylamine

In our initial studies, benzylamine was chosen as model amine in reactions with α-halogen-substituted hydrazones **1**. After brief optimization of the reaction conditions (solvent, base and ratio of reagents), it was found that alkylation of benzylamine with 2.0 equiv of Boc-hydrazone **1a** and 2.0 equiv of potassium carbonate as a base in MeOH led to bishydrazone **2a** in highest yield. The bright yellow color appeared in course of reagents mixing indicating the formation of azoalkene intermediate **A** [35–39]. Under these conditions, a range of other α-halogen hydrazones **1b–d**,**f**,**g** were successfully converted to corresponding bishydrazones **2b–d**,**f**,**g** in good to high yields (Table 1). In case of **1e**, bearing a benzoyl group, the formation of a complex mixture was observed and target product **2e** was not isolable (Table 1, entry 5).

**Table 1:** Reaction of α-halogen-substituted hydrazones **1** with benzylamine.

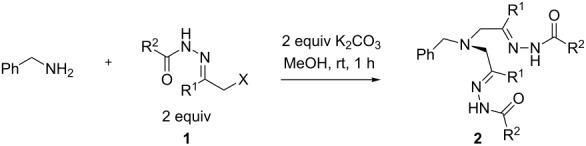					

Entry	**1**	**2**	R^1^	R^2^	Yield, %^a^

1	**a**	**a**	CH_3_	O*t*-Bu	92
2	**b**	**b**	CH_3_	OEt	87
3	**c**	**c**	CH_3_	CH_3_	84
4	**d**	**d**	CH_3_	(CH_2_)_6_CH_3_	76
5	**e**	**e**	CH_3_	Ph	–^b^
6	**f**	**f**	Ph	O*t*-Bu	82
7	**g**	**g**	CO_2_Et	O*t*-Bu	66

^a^Isolated yields. ^b^Complex mixture of products.

### Variation of the amine component in the reaction with chloroacetone hydrazone **1a**

**Figure 2:**
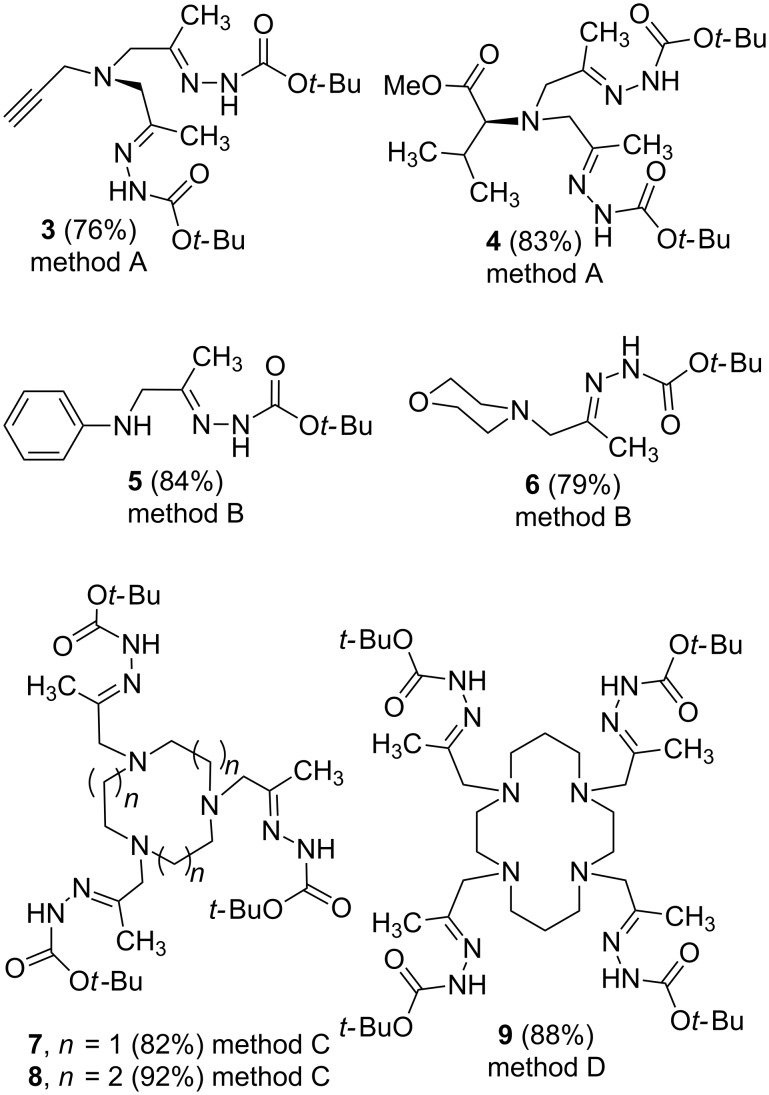
Structures of polyhydrazones **3**-**9**. Methods: A: 1 equiv of amine, 2 equiv of **1a**, 2 equiv of K_2_CO_3_; B; 1 equiv of amine, 1 equiv of **1a**, 1 equiv of K_2_CO_3_; C; 1 equiv of amine, 3.1 equiv of **1a**, 3 equiv of K_2_CO_3_; D. 1 equiv of amine, 4.2 equiv of **1a**, 4 equiv of K_2_CO_3_. Yield based on the amine used.

The suggested reaction conditions were successfully extended to a range of primary and secondary amines providing corresponding polyhydrazones **3**–**9** (Figure 2).

Thus, propargylamine and (L)-valine methyl ester (generated in situ from the corresponding hydrochloride and an additional equivalent of potassium carbonate) in the reaction with two equivalents of chloroacetone hydrazone **1a** provided the corresponding functionalized bishydrazones **3** and **4** in good yields (method A in Figure 2). On the other hand, an aromatic amine (aniline) under the aforementioned conditions led to monohydrazone **5** as a major product. Even when a 3-fold excess of **1a** was used, a mixture of mono- and bisadducts was obtained. This may be attributed to the reduced nucleophility of the secondary amino group in the primarily formed adduct **5**. The reaction of aniline with 1.0 equiv of chlorohydrazone **1a** gave **5** in 84% yield (method B in Figure 2). Similarly, the reaction with a secondary amine (morpholine) according to this procedure provided the monoalkylated adduct **6** in good yield.

Importantly, secondary polyamines could be exhaustively alkylated with chloroacetone hydrazone **1a** demonstrating the efficiency of our approach for the synthesis of polyhydrazones. Thus, treatment of macrocyclic polyamines tacn (1,4,7-triazacyclononane), tacd (1,5,9-triazacyclododecane) and cyclam (1,4,8,11-tetraazacyclotetradecane) with **1a** gave the corresponding tris- and tetra-hydrazones **7**, **8** and **9**, respectively, in high yields (methods C,D in Figure 2, a small excess of **1a** was used to ensure complete alkylation). Macrocyclic polynitrogen ligands with several hydrazone arms may be of interest for the design of sensors [52] and contrast agents [53].

Unfortunately, alkylation of ethylenediamine with 4 equivalents of **1a** led to an indecipherable mixture of products. In this case, the primary alkylation adducts might be unstable and undergo heterocyclization reactions (on the synthesis of heterocyclic compounds from azoalkenes and diamines see [54–56]).

**Scheme 3:**
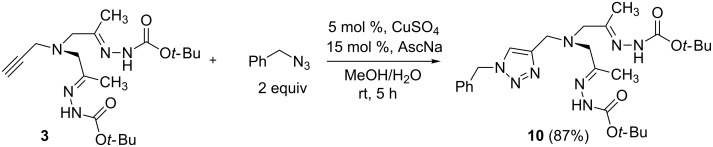
Synthesis of a mixed triazole-hydrazone ligand **10**.

Bishydrazones containing clickable groups (like **3**) can be introduced into functional molecules or immobilized on a support. This was demonstrated by the synthesis of a mixed triazole-hydrazone ligand **10** by CuAAC reaction of **3** with phenyl azide (Scheme 3) (for application of mixed triazole-imine ligands see [31–32,34]).

### Reaction of α-halogen-substituted hydrazones **1** with ammonia

Addition of α-halohydrazones **1** to ammonia (Table 2) have a special significance because the expected trishydrazones **11** are obvious analogs of tris(iminomethyl)amines widely used in the catalysis of azide–alkyne cycloadditions [29–32,34]. Furthermore, intramolecular cyclotrimerization of C=N bonds in trishydrozones would lead to unusual 1,4,6,10-tertraazaadamantane derivatives (vide infra) [57–60].

**Table 2:** Synthesis of trishydrazones **11**.

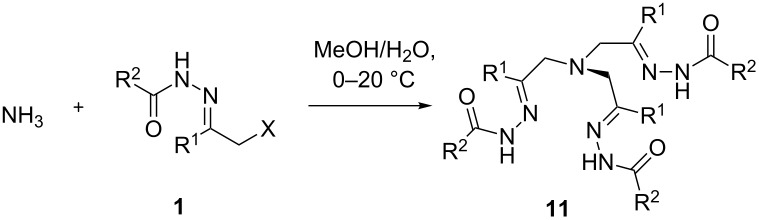					

Entry	**1**	**11**	R^1^	R^2^	Yield, %

1	**a**	**a**	CH_3_	O*t-*Bu	83
2	**b**	**b**	CH_3_	OEt	46
3	**d**	**d**	CH_3_	(CH_2_)_6_CH_3_	73
4	**f**	**f**	Ph	O*t-*Bu	65^a^
5	**h**	**h**	H	O*t-*Bu	66^b^

^a^Secondary amine HN(CH_2_C(=N-NHBoc)Ph)_2 _**12f** was also isolated in 24% yield. ^b^Yield on two steps from BocNHNH_2_.

The treatment of model hydrazone **1a** in MeOH with an excess of aqueous ammonia led to the desired trishydrazone **11a** without the formation of corresponding primary and secondary amines or quaternary ammonium salts (Table 2, entry 1). Other hydrazones of α-haloketones **1b**,**d**,**f** and the hydrazone of chloroacetaldehyde **1h** were successfully involved in the reaction with ammonia providing the corresponding trishydrazones **11b**,**d**,**f**–**h** in moderate to good yields (Table 2). In the case of phenyl-substituted hydrazone **1f**, a bis-adduct **12f** was obtained in addition to trishydrazone **11f** (Table 2, entry 4).

### Cyclization of trishydrazones **11**

**Scheme 4:**
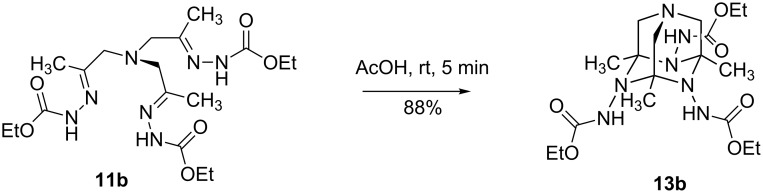
Cyclisation of 11b into 1,4,6,10-tetraazaadamantane derivative.

Upon treatment with acetic acid, trishydrazone **11b** underwent a remarkable transformation to the tetraazaadamantane derivative **13b** via intramolecular cyclotrimerization of C=N bonds (Scheme 4). A similar reaction leading to *N*-hydroxy-substituted 1,4,6,10-tetraazadamantanes was recently observed by us for trisoximes [57–60]. However, 1,4,6,10-tetraazaadamantanes with three N-amino groups are not accessible by the previously reported method from trisoximes [57–59]. Tetraazaadamantane with this substitution pattern is a promising platform for the design of supramolecular recognizing systems and for the construction of new molecular cage architectures.

**Figure 3:**
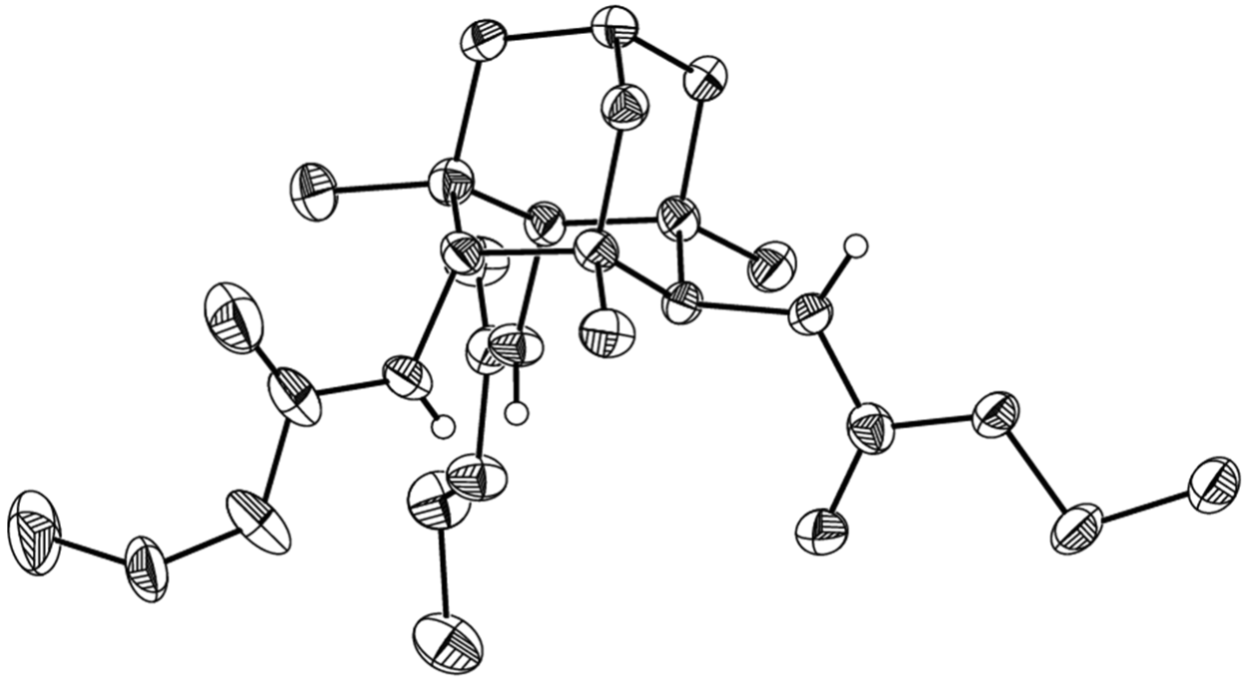
General view of **13b** in representation of atoms with thermal ellipsoids at 50% probability level; all hydrogen atoms (except for those of the NH groups) are omitted for clarity. The compound crystallizes as a crystallosolvate with two water molecules and one methanol entity (those are not shown) per two symmetry-independent molecules of the product. CCDC 1501437 contains the supplementary crystallographic data for **13b**. These data can be obtained free of charge via http://www.ccdc.cam.ac.uk/conts/retrieving.html (or from the CCDC, 12 Union Road, Cambridge, CB21EZ, UK; or deposit@ccdc.cam.ac.uk).

The formation of the 1,4,6,10-tetraazaadamantane cage was unambiguously confirmed by X-ray analysis of the crystal solvate of **13b** with water and methanol (Figure 3) as well as by ^1^H and ^13^C NMR spectra.

Considering the reversible character of the imine cyclotrimerization [57,61], such a process may be viewed as a way to modulate the molecular geometry of trishydrazones bearing functional fragments at nitrogen atoms. Further studies of this remarkable cyclization are ongoing.

### Structure and isomerism in hydrazones **2**–**12**

All newly obtained hydrazones were **2**–**12** characterized by ^1^H, ^13^C NMR spectroscopy and HRMS data. Most of the hydrazones were obtained as mixtures of *E*/*Z*-isomers (see Supporting Information File 1). The ratio of isomers depends on the substitution pattern and solvent. For example, the *E*,*E*-isomer was predominant for **2a** in DMSO-*d*_6_, while in CDCl_3_ *E*,*Z*-**2a** was the major isomer. The assignment of stereoisomers was performed using known correlations between the configuration of the C=N bond and the chemical shift of hydrogen and carbon atoms attached to it [62].

## Conclusion

In conclusion, we developed a convenient approach for the synthesis of hitherto unknown poly(hydrazonomethyl)amines **I** from α-haloketones, hydrazides and simple amines (ammonia). Using this combinatorial approach, a series of new prospective bis-, tris- and tetrahydrazone ligands were prepared. Trishydrazone **11b** was shown to undergo an intramolecular cyclotrimerization of the C=N bonds resulting in the formation of the respective *N*-amino-substituted 1,4,6,10-tetraazaadamantane derivative. Further studies of coordination chemistry aspects of poly(hydrazonomethyl)amines **I** and their applications as ligands in transition metal catalysis are currently underway.

## Supporting Information

File 1 Experimental procedures, characterization data for new compounds, copies of ^1^H and ^13^C NMR spectra.

File 2 Crystal structure file for compound **13b**.
