# Supplementary material for: Construction of bis-, tris- and tetrahydrazones by addition of azoalkenes to amines and ammonia
Source: Beilstein J Org Chem. 2016 Nov 21;12:2471–7. doi: 10.3762/bjoc.12.241 (PMC5238579; doi:10.3762/bjoc.12.241)
Supplement: File 1 — Experimental procedures, characterization data for new compounds, copies of 1H and 13C NMR spectra. [file Beilstein_J_Org_Chem-12-2471-s001.pdf]

Supporting Information

for

Construction of bis-, tris- and tetrahydrazones by addition of azoalkenes to amines and ammonia

Artem N. Semakin[1,2]*, Aleksandr O. Kokuev[2], Yulia V. Nelyubina[3], Alexey Yu. Sukhorukov[1], Petr A. Zhmurov[1], Sema L. Ioffe[1,2], and Vladimir A. Tartakovsky[1]

Address: [1]Laboratory of functional organic compounds, N.D. Zelinsky Institute of Organic Chemistry Russian Academy of Sciences, Leninsky Prospect, 47, Moscow, 119991, Russia, [2]Moscow Chemical Lyceum 1303, Tamozhenniy proezd, 4, Moscow, 111033, Russia and [3]Laboratory for X-Ray Diffraction Studies, A.N.Nesmeyanov Institute of Organoelement Compounds of Russian Academy of Sciences, Vavilova Str. 28, Moscow, 119991, Russia

Email: Artem N. Semakin* - artyomsemakin@mail.ru

*Corresponding author

Experimental procedures, characterization data for new compounds, copies of ^{1}H and ^{13}C NMR spectra

Contents

Experimental part S2

Data for compounds and copies of NMR spectra S5

X-ray data for 2(**13b**)·$2H_2O$·MeOH S51

Experimental part

Reactions were monitored by analytical TLC using silica gel TLC plates with QF-254. Visualization was accomplished with UV light and staining with a solution of ninhydrin in methanol. NMR spectra were acquired on Bruker AM300 and AC200 spectrometers at 297 K with residual solvents peaks as an internal standard. Coupling constants (*J*) are given in Hz. The ratio of *E*/*Z*-fragments is determined by NMR and refers to the ratio of isomers in solution at the moment of spectra acquisition. HRMS spectra were acquired on Bruker MicrOTOF instrument. Elemental analyses were performed at the Analytical center of N.D. Zelinsky Institute of Organic Chemistry. Melting points (uncorrected) were determined on a Kofler hot-stage microscope. Commercial reagents were used without additional purification. Compound **1g** was prepared accordingly to a literature procedure.[1]

Synthesis of α-chloro hydrazones 1

To a solution of acylhydrazine (10 mmol) in MeOH (20 mL) acetic acid (15 mmol) was added. The solution was cooled on an ice-bath and α-haloketone (15 mmol of chloroacetone, dropwise; 10 mmol of phenacylchloride, in one portion; or 30 mmol of chloroacetaldehyde (50% w/w in water), in one portion) was added. The reaction mixture was kept at same temperature for 0.5–2 h (TLC control of conversion) and further isolation of product was performed as following:

For products **1c**–**e**: The precipitate was filtered, washed with chilled (approx. −20 °C) MeOH (5 mL) and dried on a filter.

For products **1a**,**g**: The reaction mixture was poured in cold water (100 mL), the precipitate was filtered, washed with water and dried on filter.

For product **1b**: The reaction mixture was evaporated, the residue triturated with Et_2O (15 mL) and dried in vacuo (0.1 Torr).

For product **1h**: The reaction mixture was poured in cold water (100 mL) and extracted with Et_2O (100 ml). After washing with brine (50 ml) and drying with Na_2SO_4, the extract was evaporated (25 °C) and crude **1h** was used without additional purification.

NMR and physical data for compounds **1** are in accordance with literature data (**1a** and **1b**,[2] **1e**,[3] **1f**[4]).

[1] Clarke, S.; Gilchrist, T.; Lemos, A.; Roberts, T. *Tetrahedron* **1991**, *47*, 5615-5624
[2] A. Attanasi, O.; De Crescentini, L.; Giorgi, R.; Perrone, A.; Santeusanio, S. *Heterocycles* **1996**, *43*, 1447.
[3] Gillis, B.Kadunce, R. *J. Org. Chem.* **1967**, *32*, 91-94.
[4] Chen, J.; Dong, W.; Candy, M.; Pan, F.; Jörres, M.; Bolm, C. *J. Am. Chem. Soc.* **2012**, *134*, 6924-6927.

Table 1: Synthesis of α-halogen-substituted hydrazones **1** from α-halocarbonyl compounds and acyl hydrazines or carbazates.

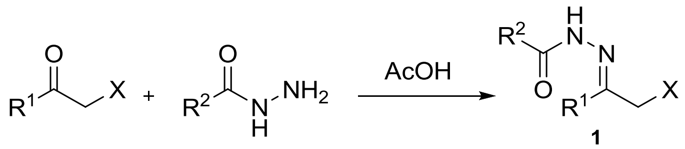

Entry	1	R[1]	R[2]	Procedure[a]	X	Yield, %
1	**a**	CH_3	O^{t}Bu	A	Cl	98[b]
2	**b**	CH_3	OEt	A	Cl	92[b]
3	**c**	CH_3	CH_3	A	Cl	45[b]
4	**d**	CH_3	$(CH_2)_6CH_3$	A	Cl	77[b]
5	**e**	CH_3	Ph	A	Cl	55[b]
6	**f**	Ph	O^{t}Bu	B	Cl	89
7	**g**	CO_2Et	O^{t}Bu	C	Br	65[c]
8	**h**	H	O^{t}Bu	D	Cl	-[d]

[a]Procedures: A: 1.5 equiv of α-haloketone, 1.0 equiv of hydrazide, 1.5 equiv of AcOH, MeOH, 0 °C; B: 1.0 equiv of α-haloketone, 1.0 equiv of hydrazide, 1.5 equiv of AcOH, MeOH, 0 °C; C: 1.0 equiv of α-haloketone, 1.5 equiv of hydrazide, 0.05 equiv of AcOH, Et_2O, 0 °C [1]; D: 3 equiv of chloroacetaldehyde, 1.0 equiv of hydrazide, 1.5 equiv of AcOH, MeOH, 0 °C. [b]Yield based on hydrazide used. [c]Yield based on α-haloketone. [d]Was used in further step in crude form due to low stability.

Reaction of α-halogen hydrazones with amines

To a solution of amine or its hydrochloride salt (1.0 mmol) in MeOH (7 mL) K_2CO_3 (1.0 mmol for morpholine and aniline, 2.0 mmol for benzylamine and propargylamine, 3.0 mmol for valine methyl ester hydrochloride and tacd, 4.0 mmol for cyclam, 6.0 mmol for tacn tryhydrochloride) was added and the mixture was stirred for 30 min. Then α-halohydrazone (1.0 mmol for morpholine and aniline, 2.0 mmol for benzylamine, propargylamine and valine methyl ester hydrochloride, 3.1 mmol for tacn and tacd or 4.2 mmol for cyclam) was added in one portion with vigorous stirring. The reaction mixture was stirred for 1 h and evaporated in vacuo. Water (50 mL) was added to the residue and further purification was performed as following:

For products **2a**–**d**, **3**–**9**: The precipitate was filtered off and washed with appropriate solvent (water for **2a**–**c**, MeOH for **7**–**9**, Et_2O for **3**–**6**, acetone for **2d**) and dried with air.

For products **2f**,**g**: EtOAc (50 mL) was added, the organic extract was separated, washed with brine (50 mL), dried with Na_2SO_4 and evaporated. The residue was purified by

column chromatography on silica gel (hexane–EtOAc (5:1) → EtOAc) to give products **2f** and **2g**.

Reaction of α-halogen hydrazones with ammonia

Trishydrazones **1a**,**b**,**h**. To a stirred solution of **1** (5 mmol) in MeOH (10 mL) aqueous ammonia (25–28%, 5 mL) was added. After 15 min water (50 mL) was added and the precipitate was filtered, thoroughly washed with water (**11a**,**h**) or water, MeOH and acetone (**11b**), and dried on filter.

Trishydrazones **1d**,**f**: To a stirred solution of **1** (2 mmol) in MeOH (5 mL) aqueous ammonia (25–28%, 1 mL) was added dropwise with cooling on ice-bath. After 15 min water (15 mL) was added, the precipitate was filtered off and further purification was performed as following:

For products **11d**: The precipitate was quickly washed with cooled (0 °C) acetone and dried in vacuo (0.1 Torr, 20 °C).

For products **11f** and **12f**: The precipitate was purified by column chromatography on silica gel (hexane–EtOAc (5:1) → EtOAc) to give **11f** and **12f**.

Reaction of 3 with BnN_3

To a stirred solution of **3** (99 mg, 0.25 mmol) in MeOH (5 ml) BnN_3 (67 mg, 0.5 mmol) was added, followed by solutions of $CuSO_4 \cdot 5H_2O$ (3.1 mg, 0.0125 mmol) in water (0.5 mL) and sodium L-ascorbate (7.4 mg, 0.0375 mmol) in water (1 mL). The reaction mixture was stirred for 5 h evaporated and water (25 mL) was added to the residue. The resulting precipitate was filtered, washed with 0.1 M EDTA solution and water, and then dried on filter to give 115 mg of **10** (87%).

Cyclization of 11b

To **11b** (443 mg, 1 mmol) AcOH (1.5 mL) was added. After ca. 5 min the starting compound dissolved forming a transparent solution. The mixture was diluted with water (30 mL) and K_2CO_3 was added in small portions until neutral pH. The solution was extracted with EtOAc (50 mL), organic extract was washed with brine (20 mL) and dried under Na_2SO_4. The solution was concentrated in vacuo, and the residue was crystallized form pentane–EtOAc, filtered, washed with pentane and dried on filter to give 390 mg of **13b** (88%).

For single-crystal X-ray diffraction analysis **13b** was recrystallized from MeOH/MTBE to give solvate **13b**$\cdot H_2O \cdot$MeOH (mp 130–138 °C) (CCDC 1501437).

Data for compounds and copies of NMR spectra

Compound **1c**

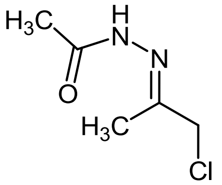

White cryst., m.p. 109-113 °C, mixture of *E*- and *Z*-**1c** in ratio 12:1.
^{1}H NMR (200 MHz, $CDCl_3$): *E*-**1c**, δ = 1.98 (s, 3 H, C*H*$_3$), 2.21 (s, 3 H, C*H*$_3$CO), 4.09 (s, 2 H, C*H*$_2$), 9.63 (s, 1 H, N*H*); selected signals of *Z*-**1c**, δ = 2.07 (s), 4.17 (s), 8.93 (s).
^{13}C NMR (50 MHz, $CDCl_3$): *E*-**1c**, δ = 13.5 (*C*H$_3$), 20.5 (*C*H$_3$CO), 49.0 (*C*H$_2$), 146.7 (*C*=N), 174.6 (*C*=O).
HRMS: Calcd for $C_5H_9ClN_2ONa$ [MH$^+$] m/z: 171.0296. Found: 171.0310.

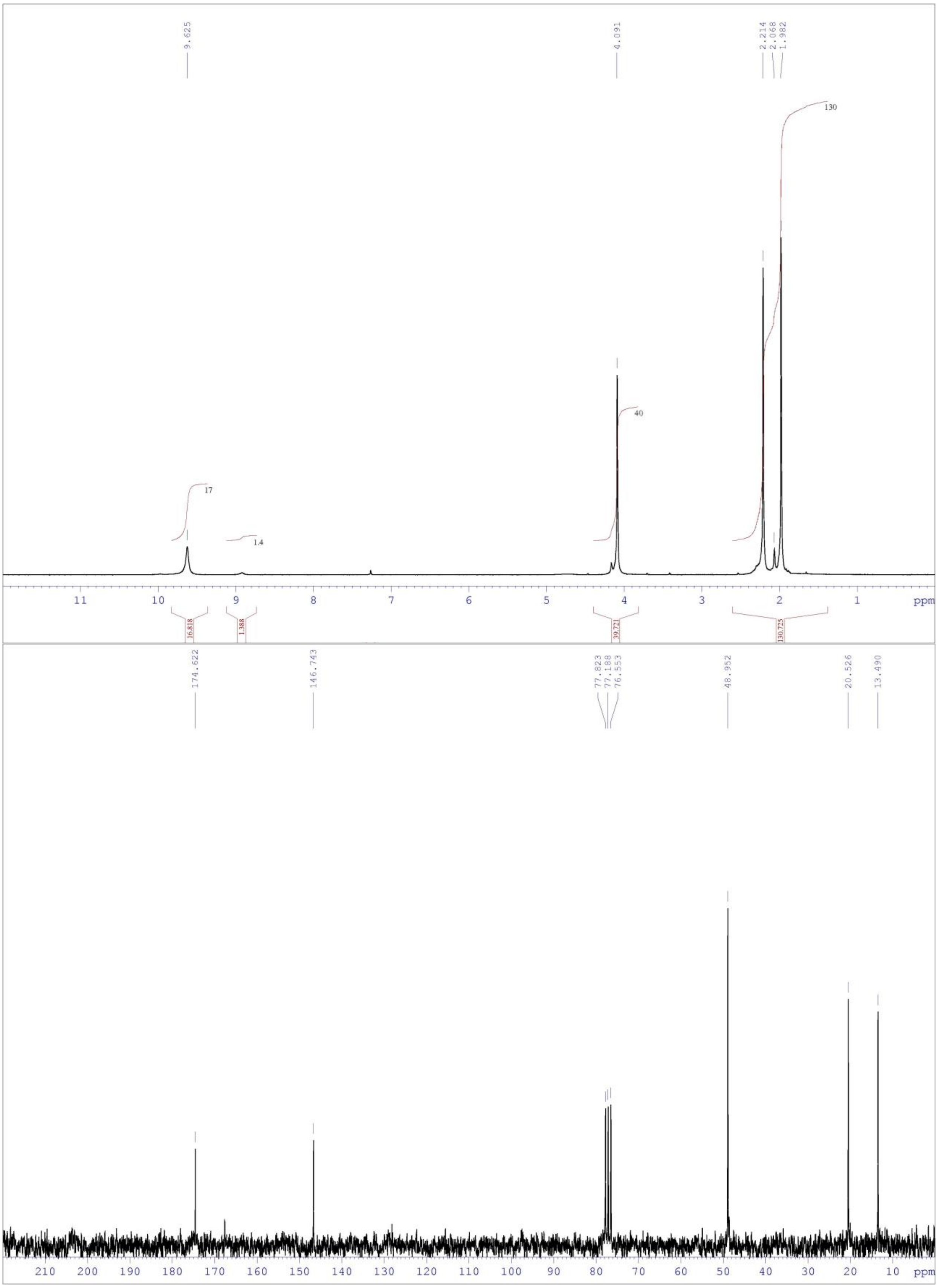
9.625
4.091
2.214
2.068
1.982
130
40
17
1.4
11 10 9 8 7 6 5 4 3 2 1 ppm
16.818
1.388
39.721
130.725
174.622
146.743
77.823
77.188
76.553
48.952
20.526
13.490
210 200 190 180 170 160 150 140 130 120 110 100 90 80 70 60 50 40 30 20 10 ppm

Compound **1d**

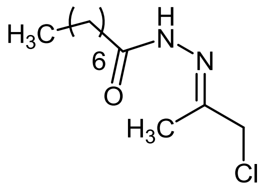

White cryst., m.p. 44-42 °C, mixture of *E*- and *Z*-**1d** in ratio 8:1.

^{1}H NMR (200 MHz, CDCl$_3$): main isomer, δ = 0.87 (m, 3 H, C*H$_3$*CH$_2$), 1.29 (m, 8 H, CH$_3$(C*H$_2$*)$_4$), 1.62 (m, 2 H, C*H$_2$*(CH$_2$)$_4$), 1.99 (s, 3 H, C*H$_3$*), 2.61 (t, *J* = 7.4, 2 H, C*H$_2$*(CH$_2$)$_5$), 4.12 (s, 2 H, C*H$_2$*), 9.34 (s, 1 H, N*H*); selected signals of minor isomer, δ = 2.09 (s), 2.30 (m), 4.21 (s), 8.47 (s).

^{13}C NMR (50 MHz, CDCl$_3$): main isomer, δ = 13.3 and 14.1 (*C*H$_3$ and *C*H$_3$CH$_2$), 22.7, 24.6, 29.1, 29.4, 31.8 and 32.6 ((*C*H$_2$)$_6$), 49.0 (*C*H$_2$), 146.0 (*C*=N), 176.9 (*C*=O).

HRMS: Calcd for $C_{11}H_{21}ClN_2ONa$ [MNa$^+$] m/z: 255.1235. Found: 255.1259.

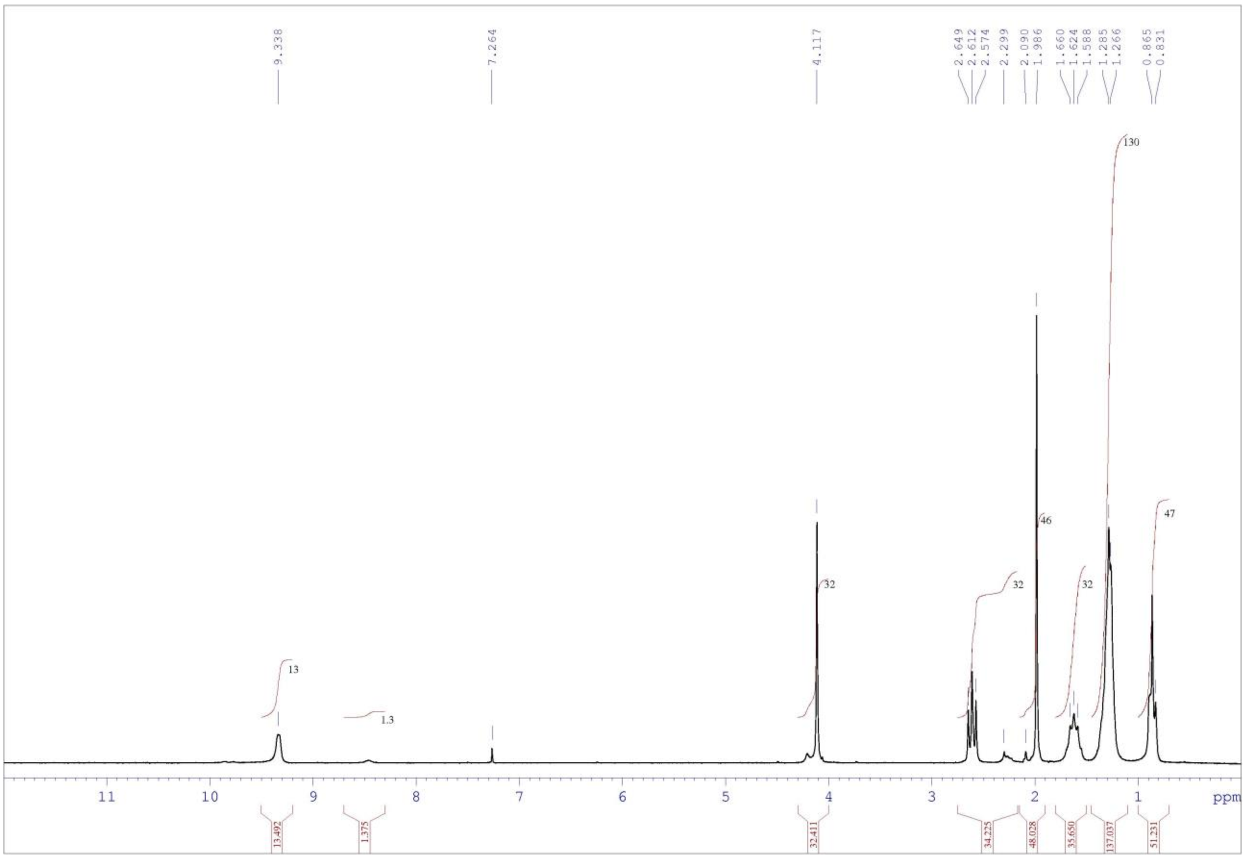
9.338
7.264
4.117
2.649
2.612
2.574
2.299
2.090
1.986
1.660
1.624
1.588
1.285
1.266
0.865
0.831
130
46
47
32
32
32
13
1.3
ppm
13.492
1.375
32.411
34.225
48.028
35.650
137.037
51.231

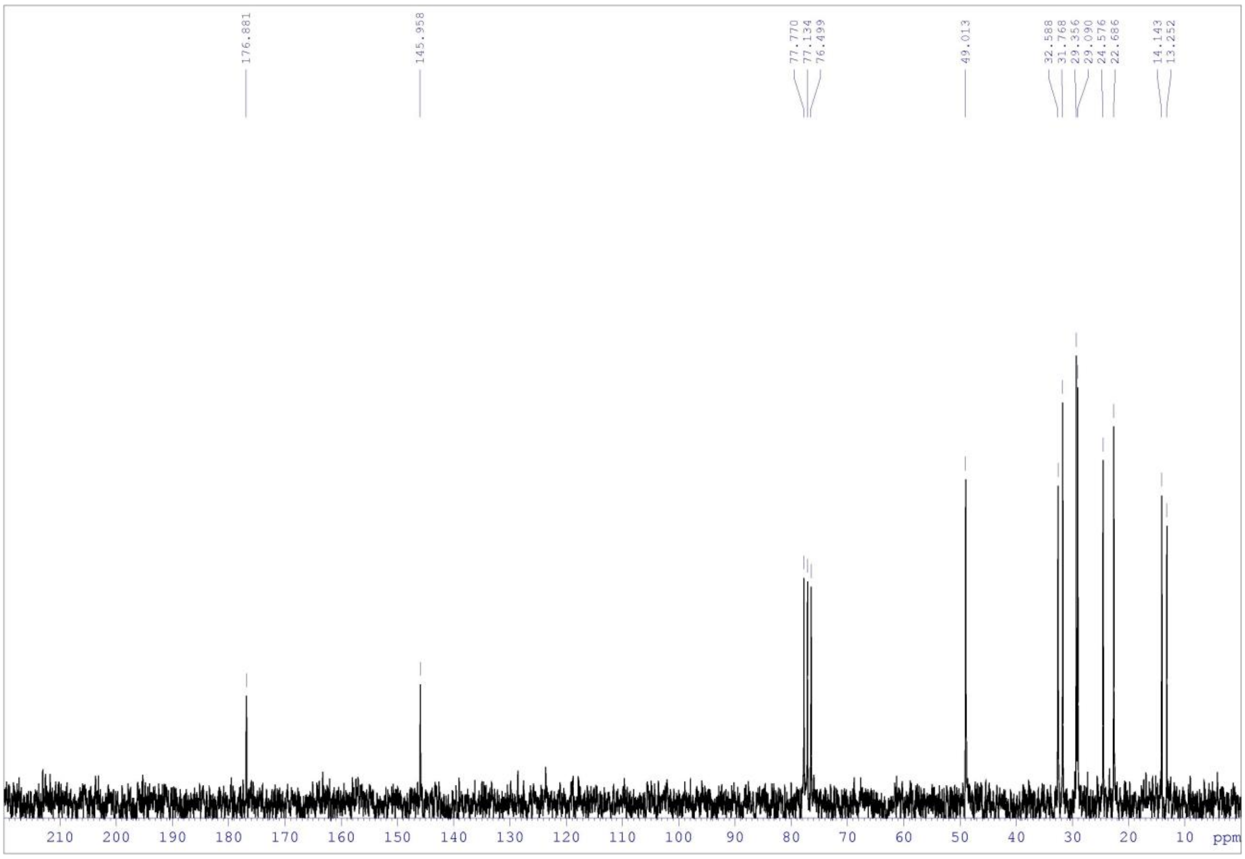
176.881
145.958
77.770
77.134
76.499
49.013
32.588
31.768
29.356
29.090
24.576
22.686
14.143
13.252
ppm

Compound **2a**

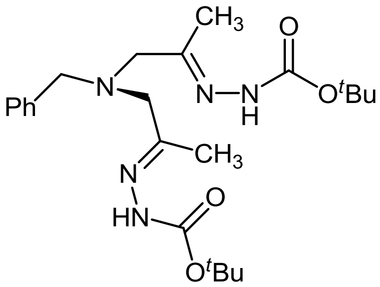

White solid, m.p. 204-206 °C, mixture of isomers with ratio of *E*- and *Z*- fragments >20:1 .
^{1}H NMR (300 MHz, DMSO-d_6): main isomer (*E*,*E*), δ = 1.45 (s, 18 H, 6 CH_3 (^{t}Bu)), 1.83 (s, 6 H, 2 CH_3), 2.97 (s, 4 H, 2 CH_2), 3.46 (s, 2 H, CH_2Ph), 7.2-7.4 (m, 5 H, *Ph*), 9.45 (s, 2 H, 2 N*H*); selected signals of minor isomers, δ = 1.77 and 1.87 (2 s), 3.21 (s), 3.50 (s).
^{13}C NMR (75 MHz, $CDCl_3$): main isomer, δ = 14.4 (2 *C*H_3), 28.1 (6 *C*H_3 (^{t}Bu)), 56.9 (*C*H_2), 59.9 (2 *C*H_2), 79.0 (2 *C* (^{t}Bu)), 126.9, 128.1, 129.0 and 138.2 (*Ph*), 151.6 and 153.1 (2 *C*=N and 2 *C*=O); selected signals of minor isomers, δ = 23.5, 55.7, 58.3, 60.7.
Elemental analysis. For $C_{23}H_{37}N_5O_4$ calcd: C, 61.72%; H, 8.33%; N, 15.65%. Found: C, 61.38%; H, 8.21%; N, 15.11%.

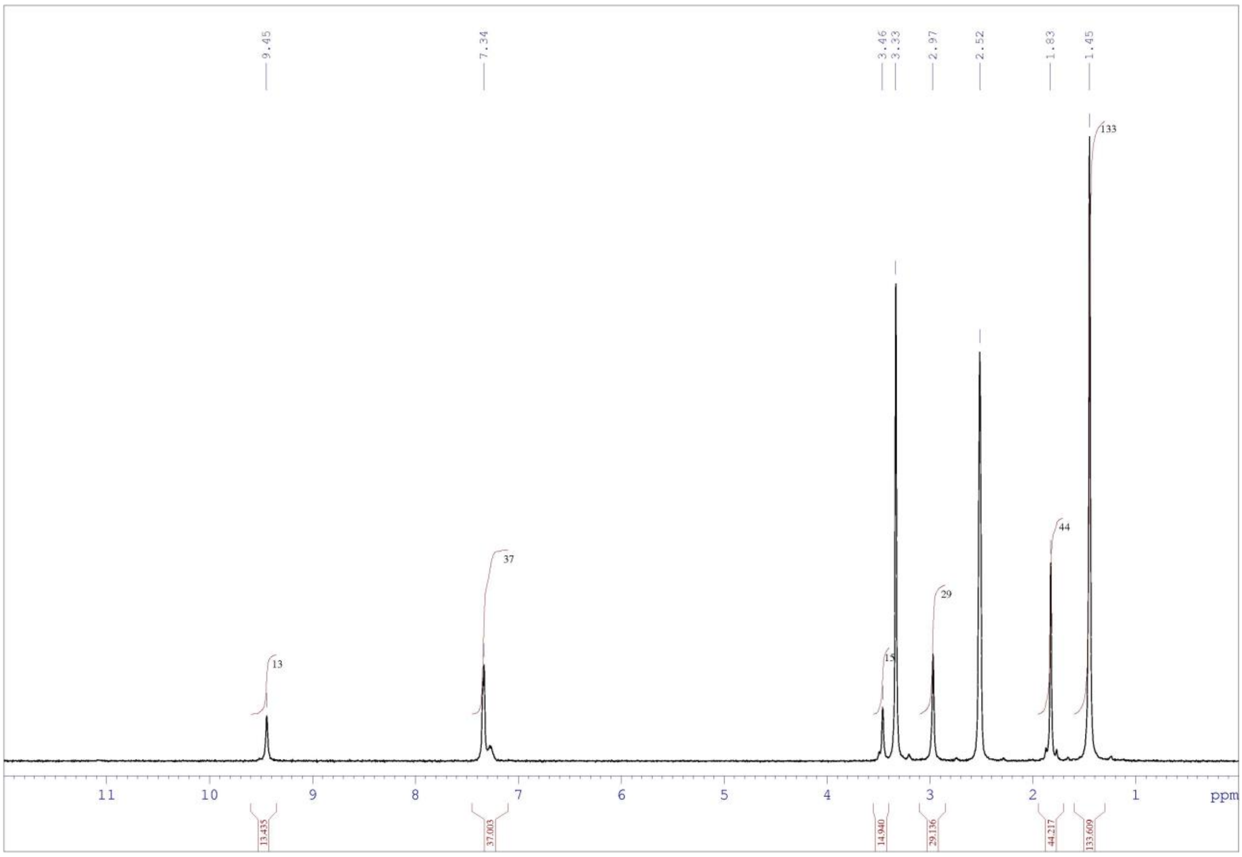
9.45
7.34
3.46
3.33
2.97
2.52
1.83
1.45
13
37
15
29
44
133
13.435
37.003
14.940
29.136
44.217
133.609
ppm

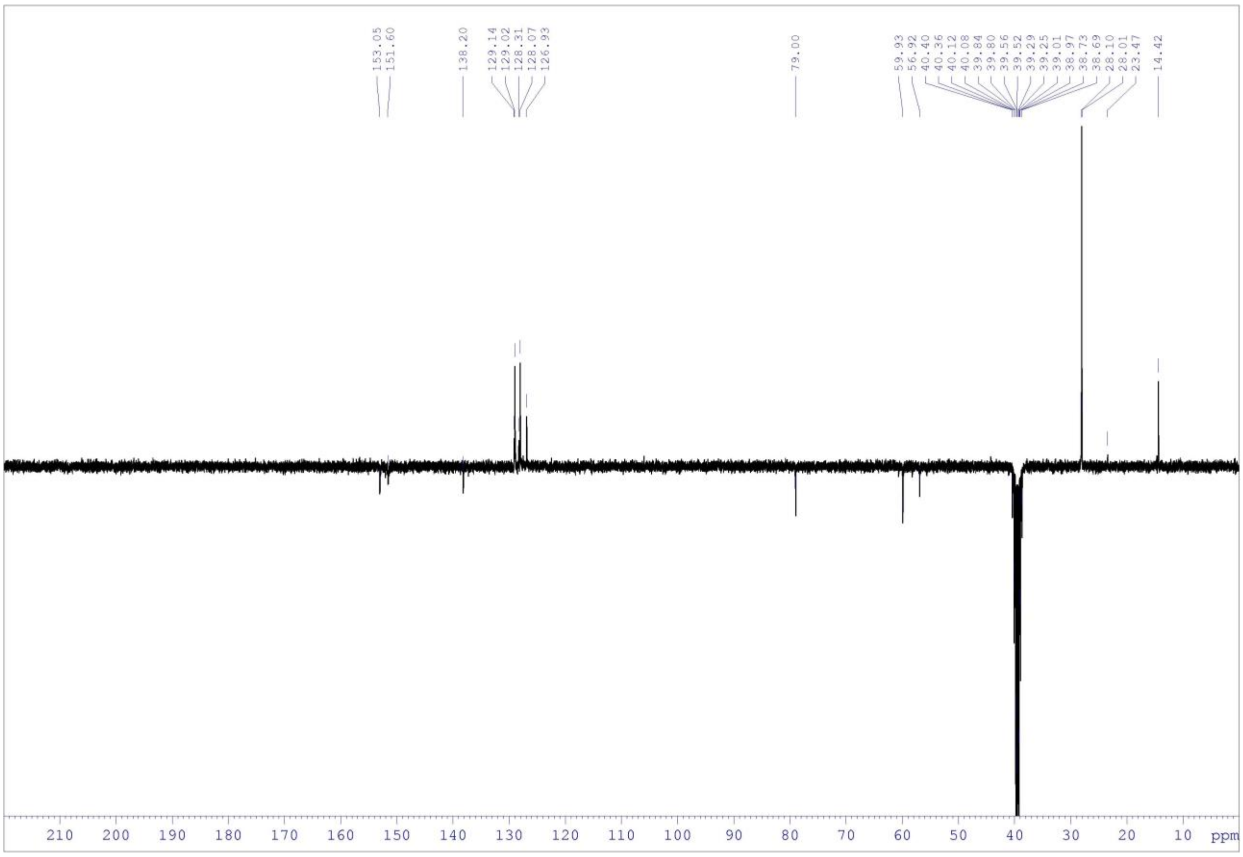
153.05
151.60
138.20
129.14
129.02
128.31
128.07
126.93
79.00
59.93
56.92
40.40
40.36
40.12
40.08
39.84
39.80
39.56
39.52
39.29
39.25
39.01
38.97
38.73
38.69
28.10
28.01
23.47
14.42
ppm

Compound **2b**

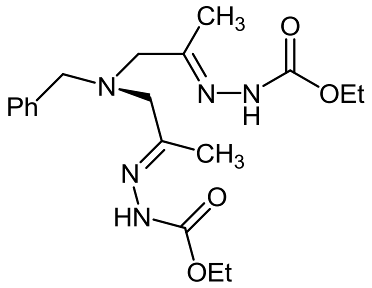

White solid, m.p. 154-158 °C, mixture of isomers with ratio of *E*- and *Z*- fragments 6:1.

^{1}H NMR (300 MHz, DMSO-d$_6$): *E*,*E*-**2b**, δ = 1.22 (t, *J* = 7.1, 6 H, 2 CH_3CH$_2$), 1.84 (s, 6 H, 2 CH_3), 3.00 (s, 4 H, 2 CH_2), 3.47 (s, 2 H, CH_2Ph), 4.11 (q, *J* = 7.1, 4 H, 2 CH$_3$CH_2), 7.2-7.4 (m, 5 H, *Ph*), 9.74 (s, 2 H, 2 NH); selected signals of minor isomers, δ = 1.79 and 1.89 (2 s), 3.04 and 3.22 (2 s), 3.51 (s), 9.81 and 11.03 (2 s).

^{13}C NMR (75 MHz, CDCl$_3$): *E*,*E*-**2b**, δ = 14.4 and 14.5 (2 *C*H$_3$ and 2 *C*H$_3$CH$_2$), 57.0 (*C*H$_2$Ph), 59.9 and 60.2 (2 *C*H$_2$ and 2 CH$_3$*C*H$_2$), 126.9, 128.0, 129.0 and 138.2 (*Ph*), 152.1 and 154.0 (2 *C*=N and 2 *C*=O); selected signals of minor isomers, δ = 14.4, 14.7, 23.2, 55.2, 58.3, 60.3, 60.5, 127.4, 128.3, 129.1, 137.2.

HRMS: Calcd for $C_{19}H_{30}N_5O_4$ [MH$^+$] m/z: 392.2292. Found: 392.2292.

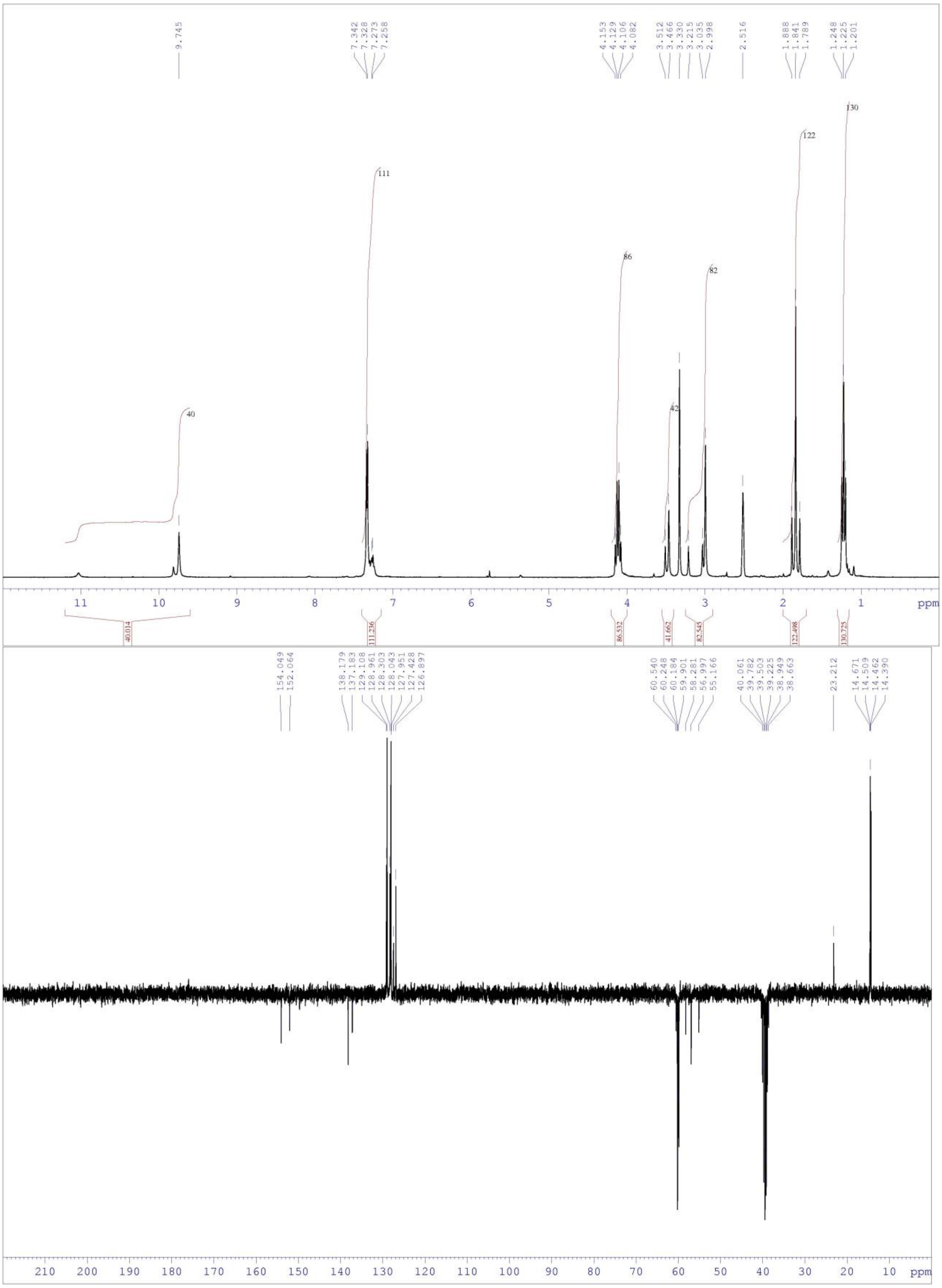
9.745
7.342
7.328
7.273
7.258
4.153
4.129
4.106
4.082
3.512
3.466
3.330
3.215
3.035
2.998
2.516
1.888
1.841
1.789
1.248
1.225
1.201
ppm
40.014
111.236
86.532
41.662
82.545
122.498
130.725
154.049
152.064
138.179
137.183
129.108
128.961
128.303
128.043
127.951
127.428
126.897
60.540
60.248
60.184
59.901
58.281
56.997
55.166
40.061
39.782
39.503
39.225
38.949
38.663
23.212
14.671
14.509
14.462
14.390
ppm

Compound **2c**

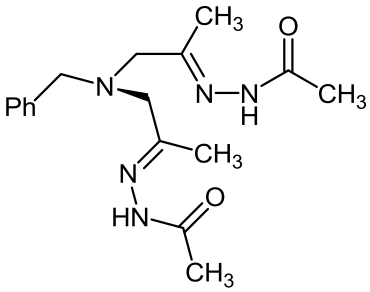

White solid, m.p. 171-173 °C, mixture of isomers with ratio of *E*- and *Z*- fragments 1.4:1.

^{1}H NMR (300 MHz, DMSO-d$_6$): *E*,*E*-**2c**, δ = 1.84 (s, 6 H, 2 C*H*$_3$), 2.10 (s, 6 H, 2 C*H*$_3$CO), 3.06 (s, 4 H, 2 C*H*$_2$), 3.52 (s, 2 H, C*H*$_2$Ph), 7.2-7.4 (m, 5 H, *Ph*), 10.05 (s, 2 H, 2 N*H*); selected signals of other isomers, δ = 1.88 (s), 1.94 (s), 3.03 (s), 3.55 (s).

^{13}C NMR (75 MHz, CDCl$_3$): *E*,*E*-**2c**,, δ = 14.3 (2 *C*H$_3$), 20.5 (2 *C*H$_3$CO), 57.5 (*C*H$_2$Ph), 60.3 (2 *C*H$_2$), 126.9, 128.0, 128.9 and 138.2 (*Ph*), 150.0 (2 *C*=N), 172.2 (2 *C*=O); selected signals of others isomers, δ = 14.6, 21.4, 60.2, 128.3, 129.1, 154.1, 165.7.

HRMS: Calcd for $C_{17}H_{26}N_5O_2$ [MH$^+$] m/z: 332.2081. Found: 332.2079.

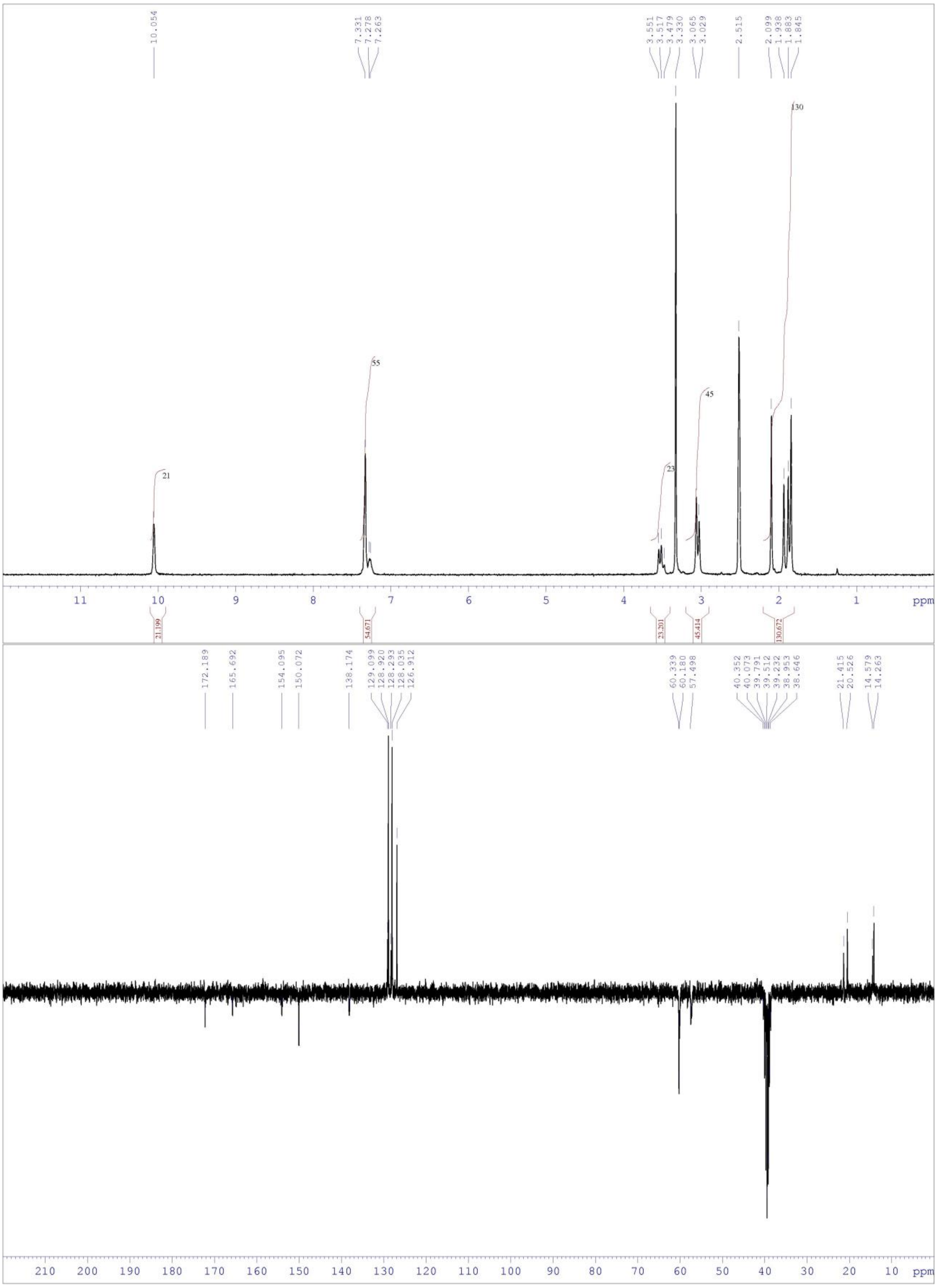
10.054
7.331
7.278
7.263
3.551
3.517
3.479
3.330
3.065
3.029
2.515
2.099
1.938
1.883
1.845
21.199
54.671
23.201
45.414
130.672
ppm
172.189
165.692
154.095
150.072
138.174
129.099
128.920
128.293
128.035
126.912
60.339
60.180
57.498
40.352
40.073
39.791
39.512
39.232
38.953
38.646
21.415
20.526
14.579
14.263
ppm

Compound **2d**

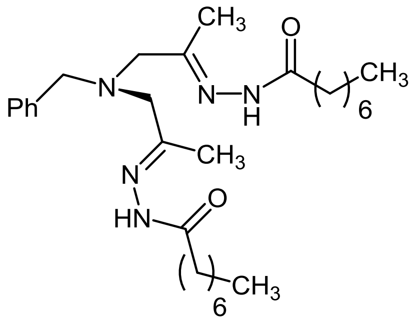

White solid, m.p. 120-124 °C, mixture of isomers with ratio of *E*- and *Z*- fragments 2:1.

^{1}H NMR (300 MHz, DMSO-d_6): δ = 0.86 (s, 6 H, 2 C*H_3* (Hept)), 1.26 (s, 16 H, 2 (C*H_2*)$_4$), 1.54 (s, 4 H, 2 C*H_2*(CH_2)$_4$) 1.86, 1.88 and 1.84 (3 s, 6 H, 2 C*H_3*), 2.10, 2.22 and 2.48 (3 s, 4 H, C*H_2*CO), 3.08 and 3.24 (2 s, 4 H, 2 C*H_2*), 3.52 and 3.54 (2 s, 2 H, C*H_2*Ph), 7.1-7.4 (m, 5 H, *Ph*), 10.00 (s, 2 H, 2 N*H*).

^{13}C NMR (75 MHz, $CDCl_3$): δ = 13.8 and 14.2 (2 *C*H_3 and 2 *C*H_3 (Hept)), 21.9, 24.0, 24.2, 25.0, 28.3, 28.6, 31.1, 32.1 and 33.8 (2 (*C*H_2)$_6$), 57.3 and 60.2 (2 *C*H_2 and *C*H_2), 126.9, 128.0, 128.3, 128.9, 129.1 and 138.2 (*Ph*), 149.8 (2 *C*=N), 174.6 (2 *C*=O).

HRMS: Calcd for $C_{29}H_{50}N_5O_2$ [MH^+] m/z: 500.3959. Found: 500.3964.

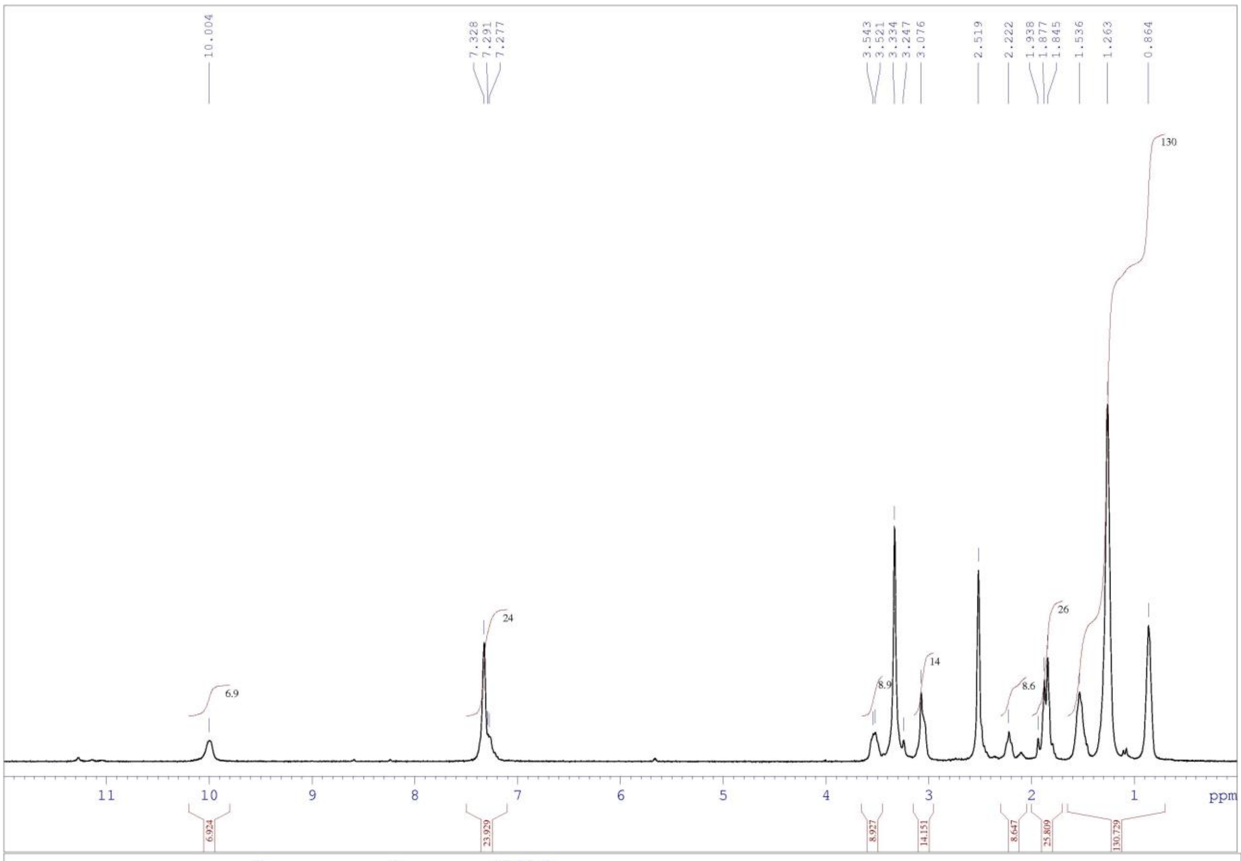
10.004
7.328
7.291
7.277
3.543
3.521
3.334
3.247
3.076
2.519
2.222
1.938
1.877
1.845
1.536
1.263
0.864
6.9
24
8.9
14
8.6
26
130
11 10 9 8 7 6 5 4 3 2 1 ppm
6.924
23.929
8.927
14.151
8.647
25.809
130.729

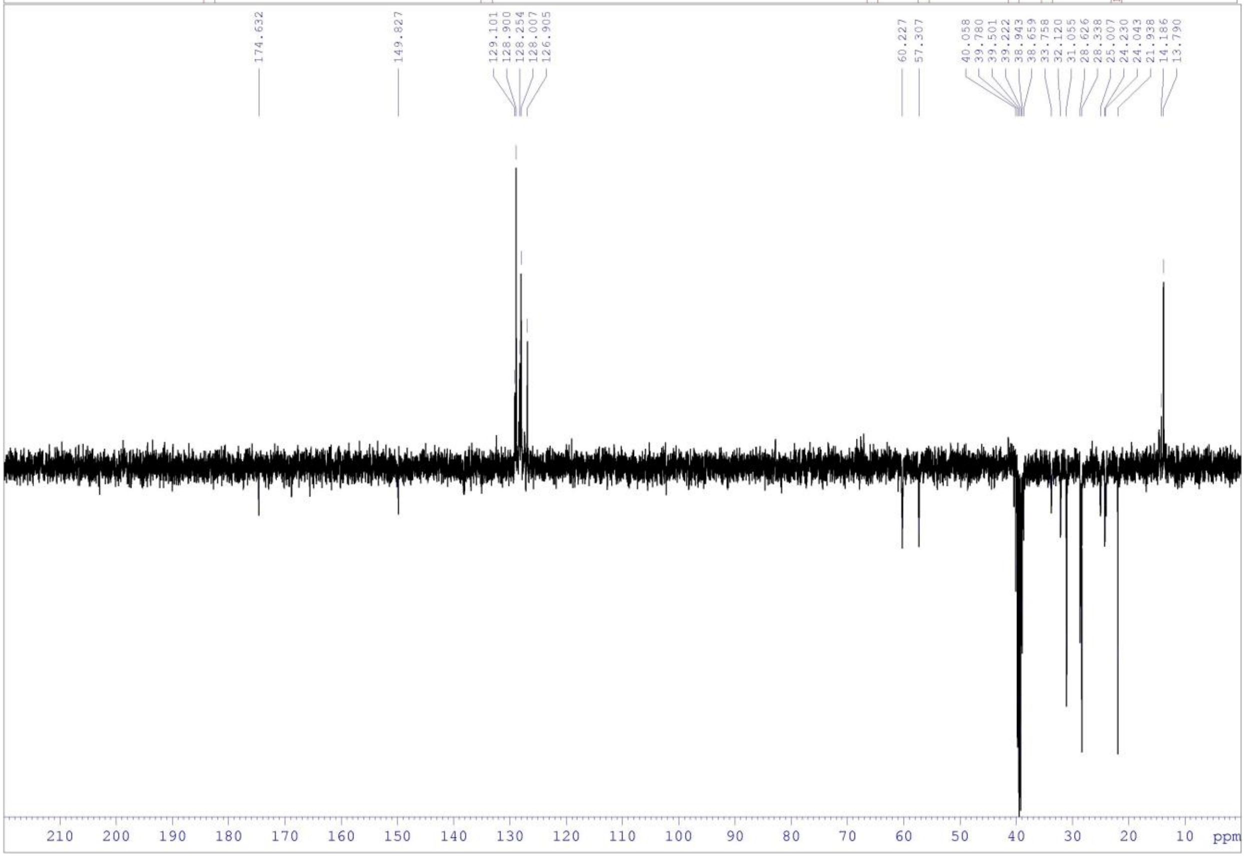
174.632
149.827
129.101
128.900
128.254
128.007
126.905
60.227
57.307
40.058
39.780
39.501
39.222
38.943
38.659
33.758
32.120
31.055
28.626
28.338
25.007
24.230
24.043
21.938
14.186
13.790
210 200 190 180 170 160 150 140 130 120 110 100 90 80 70 60 50 40 30 20 10 ppm

Compound **2f**

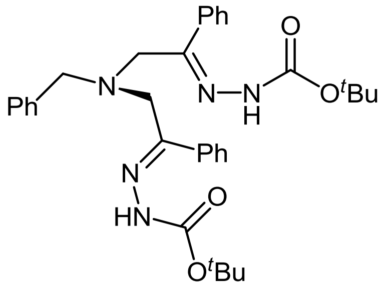

Pale yellow solid, softening at 67 °C, melting at 95 °C, mixture of isomers.
^{1}H NMR (300 MHz, DMSO-d_6): δ = 1.41, 1.43 and 1.47 (3 s, 18 H, 6 CH_3 (tBu)), 3.42, 3.51, 3.60, 3.66 and 3.76 (5 s, 6 H, 2 CH_2 and CH_2), 6.8-7.8 (m, 15 H, 2 *Ph* and *Ph*), 8.47, 8.72 and 10.56 (3 s, 2 H, 2 N*H*).
^{13}C NMR (75 MHz, DMSO-d_6): δ = 27.8 and 27.9 (6 *C*H_3 (tBu)), 56.4, 57.5 and 59.1 (2 *C*H_2 and *C*H_2), 79.1 and 79.6 (2 *C* (tBu)), 126.2, 126.6, 127.0, 127.3, 127.4, 127.6, 128.1, 128.2, 128.5, 128.6, 128.7, 128.9, 129.0, 129.4, 132.0, 132.7, 137.2, 137.5 and 137.7 (2 *Ph* and *Ph*), 150.1, 151.1, 151.7 and 152.1 (2 *C*=N and 2 *C*=O).
HRMS: Calcd for $C_{33}H_{42}N_5O_4$ [MH^+] m/z: 572.3231. Found: 572.3226.

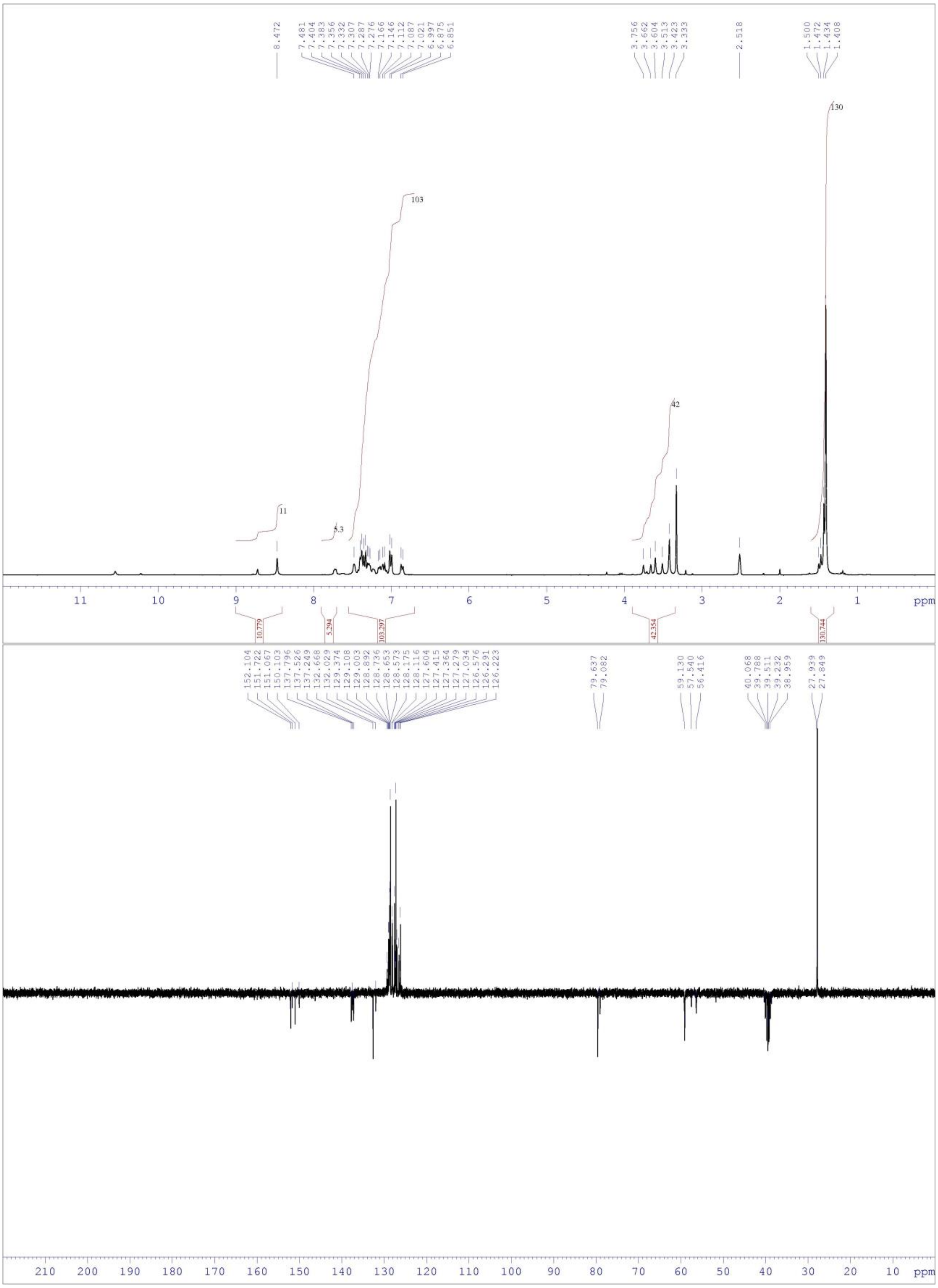
8.472
7.481
7.404
7.383
7.356
7.332
7.307
7.287
7.276
7.166
7.146
7.112
7.087
7.021
6.997
6.875
6.851
3.756
3.662
3.604
3.513
3.423
3.333
2.518
1.500
1.472
1.434
1.408
11
5.3
103
42
130
11 10 9 8 7 6 5 4 3 2 1 ppm
10.779
5.294
103.297
42.354
130.744
152.104
151.722
151.067
150.103
137.796
137.526
137.249
132.668
132.029
129.374
129.178
129.003
128.892
128.736
128.653
128.573
128.175
128.116
127.604
127.415
127.364
127.279
127.034
126.576
126.291
126.223
79.637
79.082
59.130
57.540
56.416
40.068
39.788
39.511
39.232
38.959
27.939
27.849
210 200 190 180 170 160 150 140 130 120 110 100 90 80 70 60 50 40 30 20 10 ppm

Compound **2g**

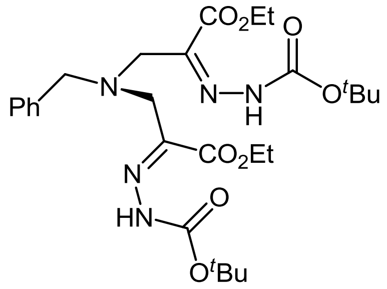

Pale yellow foam, softening at 79 °C, melting at 98 °C, mixture of isomers with ratio of *E*- and *Z*- fragments 1:7).

^{1}H NMR (300 MHz, DMSO-d$_6$): main isomer (*Z*,*Z*), δ = 1.23 (t, *J* = 7.1, 6 H, 2 C*H*$_3$CH$_2$), 1.49 (s, 18 H, 6 C*H*$_3$ (tBu)), 3.49 (s, 2 H, C*H*$_2$), 3.55 (s, 4 H, 2 C*H*$_2$), 4.17 (q, 4 H, 2 C*H*$_2$CH$_3$), 7.2-7.4 (m, 5 H, *Ph*), 10.79 (s, 2 H, 2 N*H*); selected signals of minor isomers, δ = 1.47 (s), 3.70 (s).

^{13}C NMR (75 MHz, DMSO-d$_6$): δ = 13.9 (2 *C*H$_3$CH$_2$), 27.8 (6 *C*H$_3$ (tBu)), 49.6, 58.8 and 60.8 (2 *C*H$_2$, 2 *C*H$_2$CH$_3$, *C*H$_2$), 80.7 (2 *C* (tBu)), 127.6, 128.3, 128.8 and 136.8 (*Ph*), 138.6, 151.5 and 163.7 (2 *C*=N and 4 *C*=O).

HRMS: Calcd for $C_{27}H_{41}N_5O_8Na$ [MNa^+] m/z: 586.2847. Found: 586.2839.

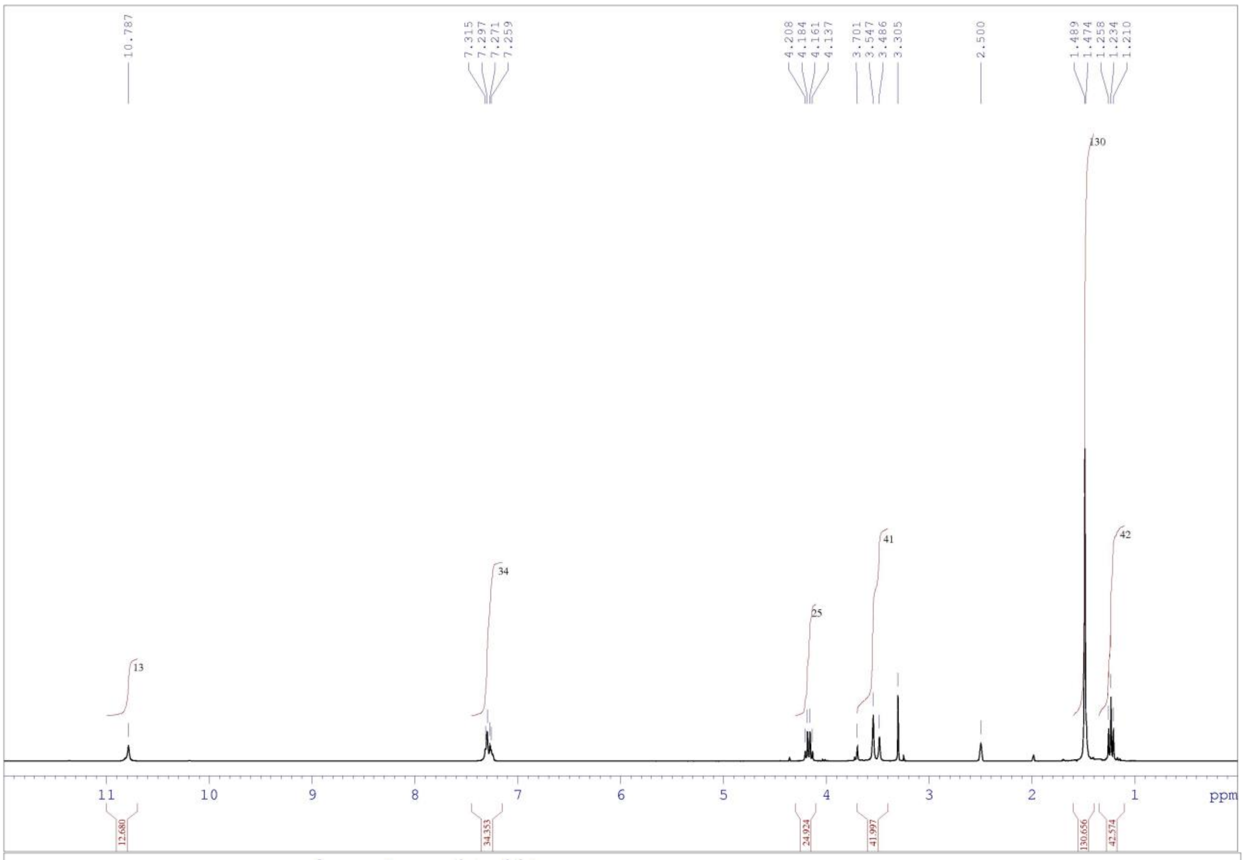
10.787
7.315
7.297
7.271
7.259
4.208
4.184
4.161
4.137
3.701
3.547
3.486
3.305
2.500
1.489
1.474
1.258
1.234
1.210
13
34
25
41
130
42
11 10 9 8 7 6 5 4 3 2 1 ppm
12.680
34.353
24.924
41.997
130.656
42.574

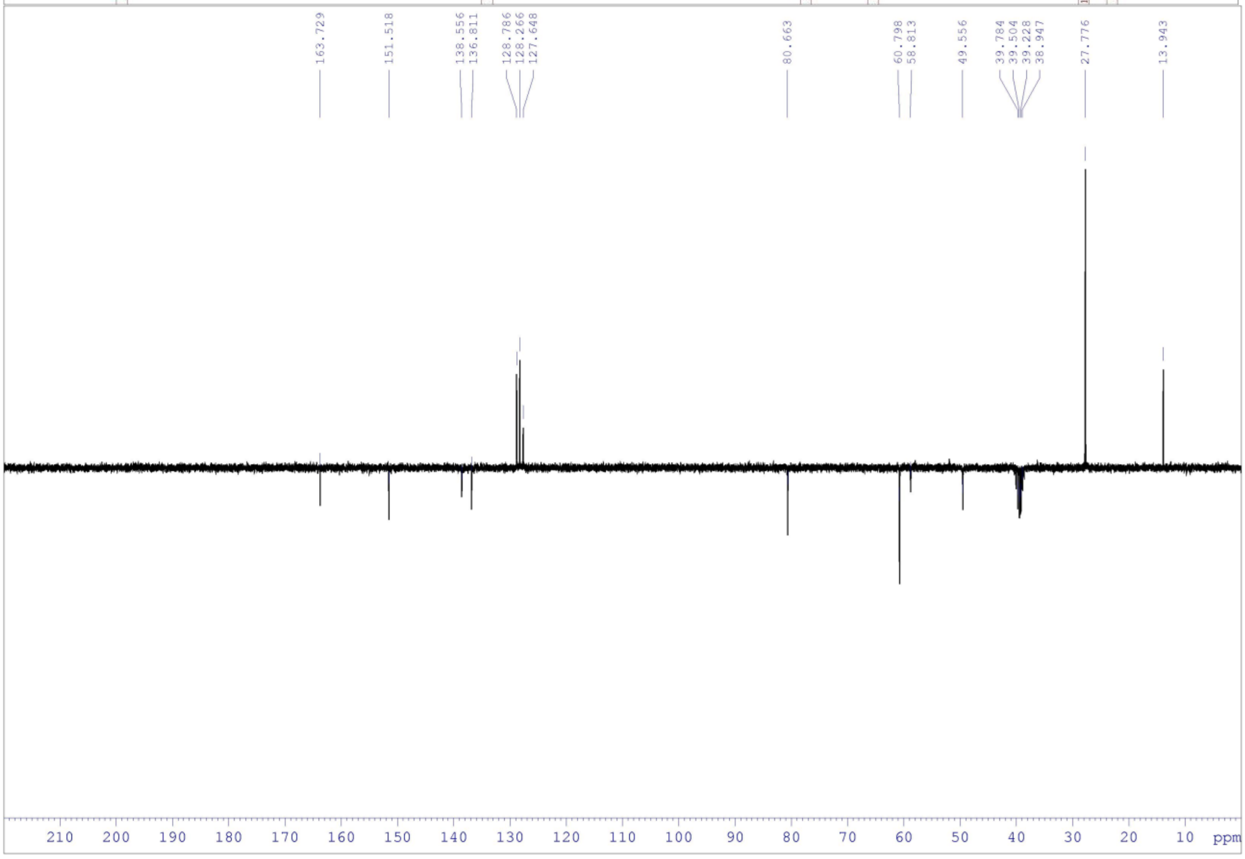
163.729
151.518
138.556
136.811
128.786
128.266
127.648
80.663
60.798
58.813
49.556
39.784
39.504
39.228
38.947
27.776
13.943
210 200 190 180 170 160 150 140 130 120 110 100 90 80 70 60 50 40 30 20 10 ppm

Compound **3**

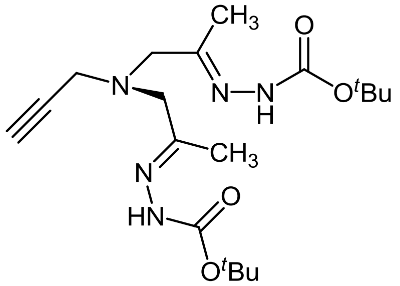

White solid, m.p. 169-174 °C (with decomposition), *E*,*E*- **3**.
^{1}H NMR (300 MHz, DMSO-d$_6$): δ = 1.45 (s, 18 H, 6 C*H$_3$* (^{t}Bu)), 1.83 (s, 6 H, 2 C*H$_3$*), 3.08 (s, 4 H, 2 C*H$_2$*), 3.14 (s, 1 H, CC*H*), 3.25 (s, 2 H, C*H$_2$*CC), 9.43 (s, 2 H, 2 N*H*).
^{13}C NMR (75 MHz, DMSO-d$_6$): δ = 14.3 (2 *C*H$_3$), 28.0 (6 *C*H$_3$ (^{t}Bu)), 41.8 (*C*H$_2$CC), 59.3 (2 *C*H$_2$), 75.7 (C*C*H), 78.8 (*C*CH), 79.0 (2 *C* (^{t}Bu)), 151.3 and 153.0 (2 *C*=N and 2 *C*=O).
HRMS: Calcd for $C_{19}H_{34}N_5O_4$ [MH^+] m/z: 396.2605. Found: 396.2600.

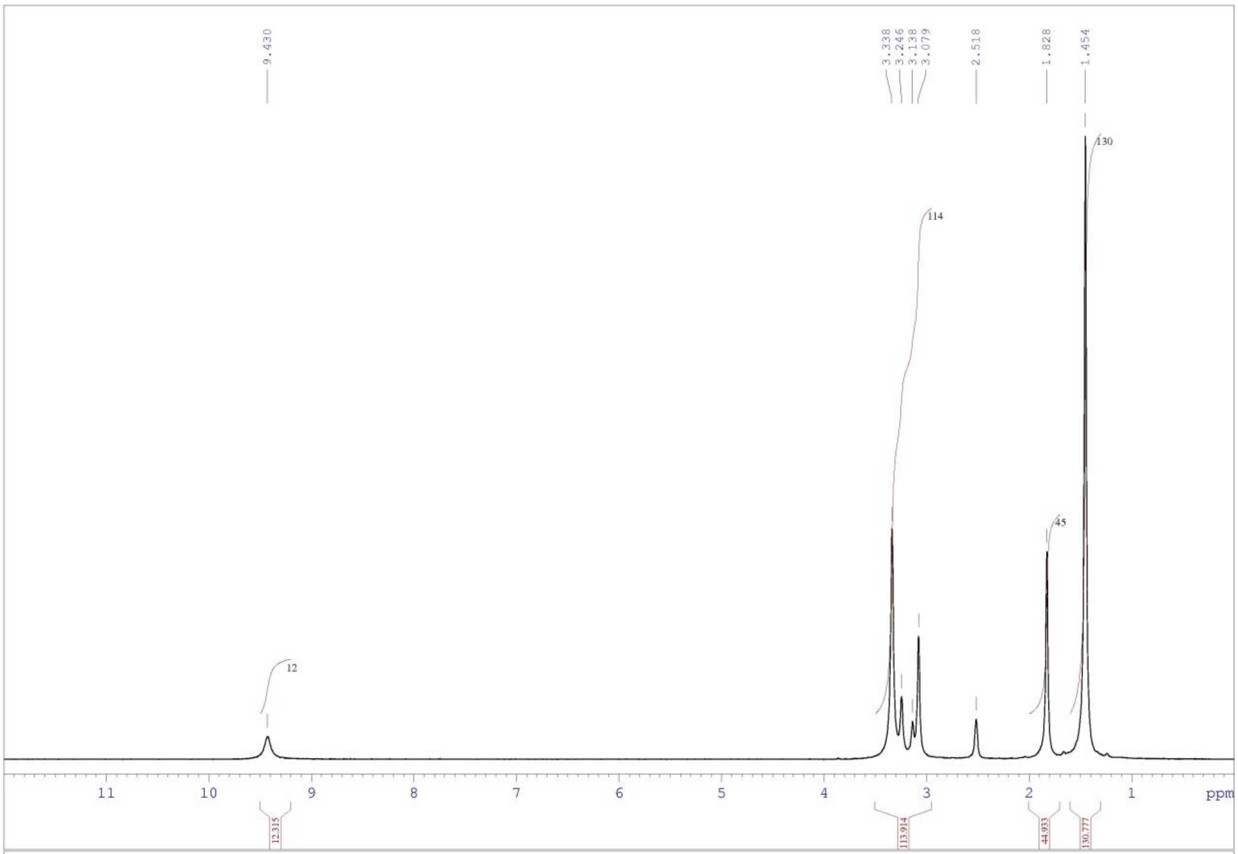

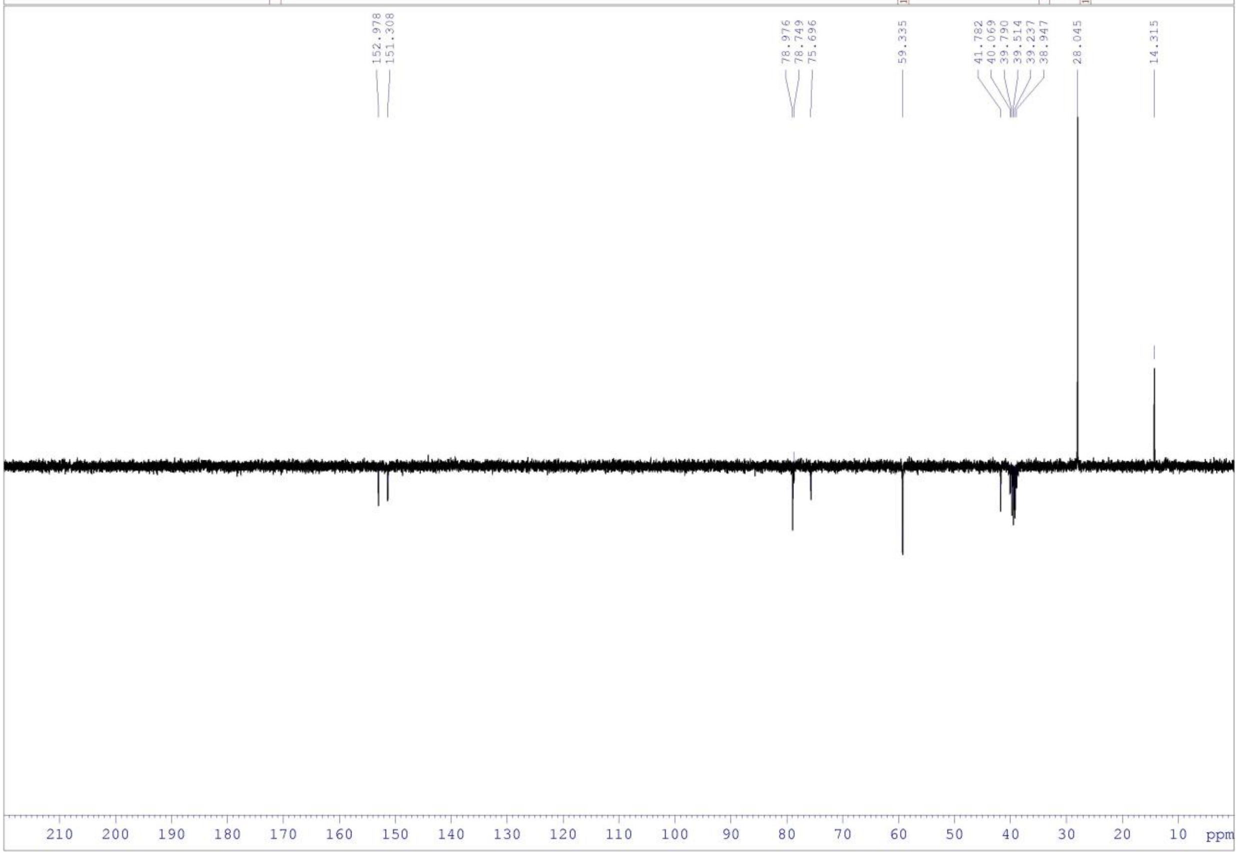

Compound **4**

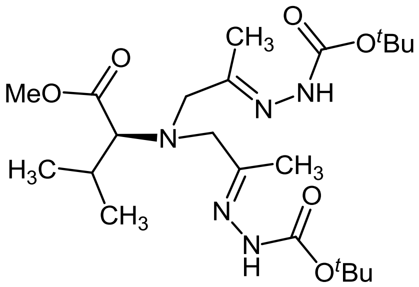

White solid, m.p. 164-166 °C. (s)-*E*,*E*-**4**.

^{1}H NMR (300 MHz, DMSO-d$_6$): δ = 0.80 and 0.96 (2 s, 6 H, 2 C*H$_3$* (^{i}Pr)), 1.45 (s, 18 H, 6 C*H$_3$* (^{t}Bu)), 1.81 (s, 6 H, 2 C*H$_3$*), 2.01 (s br, 1 H, C*H* (^{i}Pr)), 2.76 (m, 1 H, C*H*), 3.11 (dd, J_1 =9.9, J_2 = 1.32, 4 H, 2 C*H$_2$*), 3.66 (s, 3 H, C*H$_3$*O), 9.40 (s, 2 H, 2 N*H*).

^{13}C NMR (75 MHz, DMSO-d$_6$): δ = 14.5 (2 *C*H$_3$), 19.5 and 19.7 (2 *C*H$_3$ (^{i}Pr)), 26.9 (*C*H (^{i}Pr)), 28.1 (6 *C*H$_3$ (^{t}Bu)), 50.7 (*C*H), 57.3 (2 *C*H$_2$), 68.9 (*C*H$_3$O), 79.0 (2 *C* (^{t}Bu)), 151.4 and 153.0 (2 *C*=N and 2 *C*=O), 171.4 (*C*=O).

HRMS: Calcd for $C_{22}H_{42}N_5O_6$ [MH^+] m/z: 472.3130. Found: 472.3122.

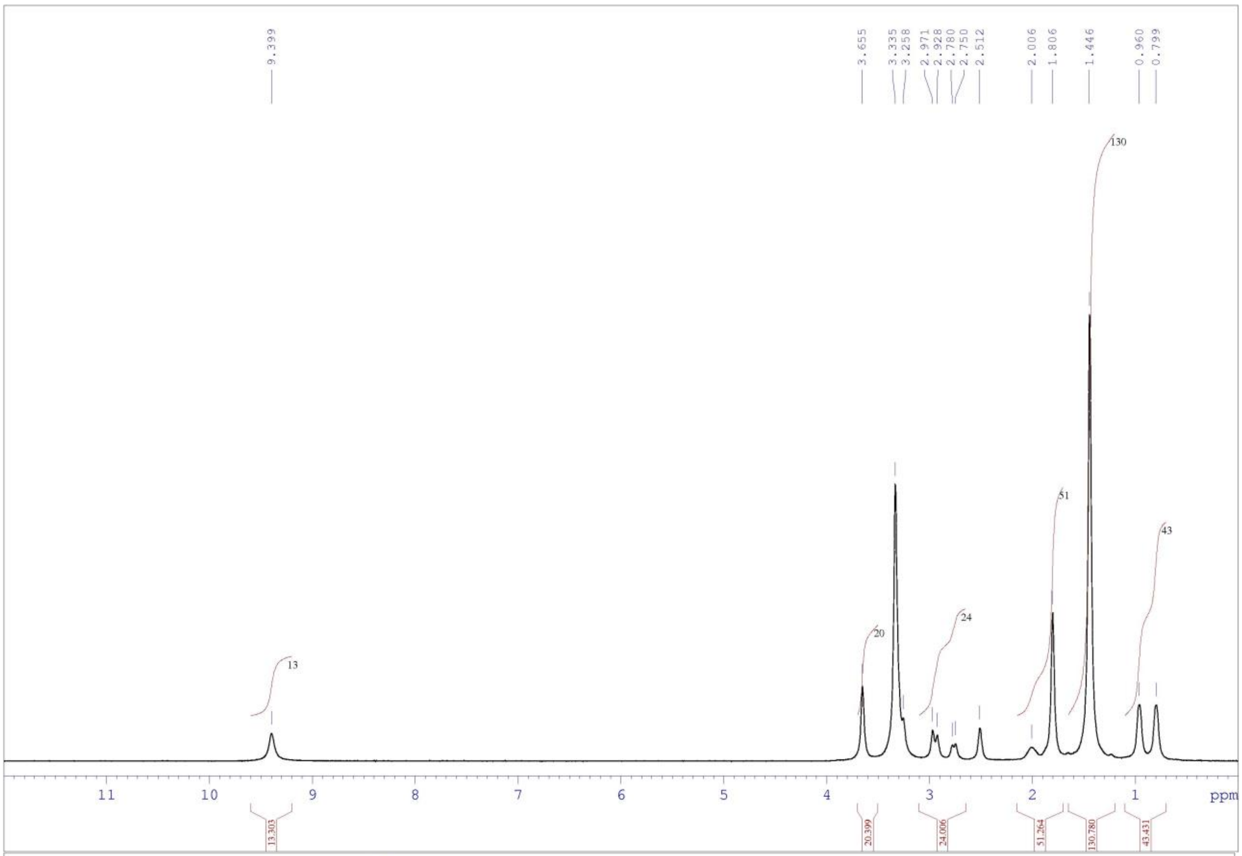
9.399
3.655
3.335
3.258
2.971
2.928
2.780
2.750
2.512
2.006
1.806
1.446
0.960
0.799
13
20
24
51
130
43
11 10 9 8 7 6 5 4 3 2 1 ppm
13.303
20.399
24.006
51.264
130.780
43.431

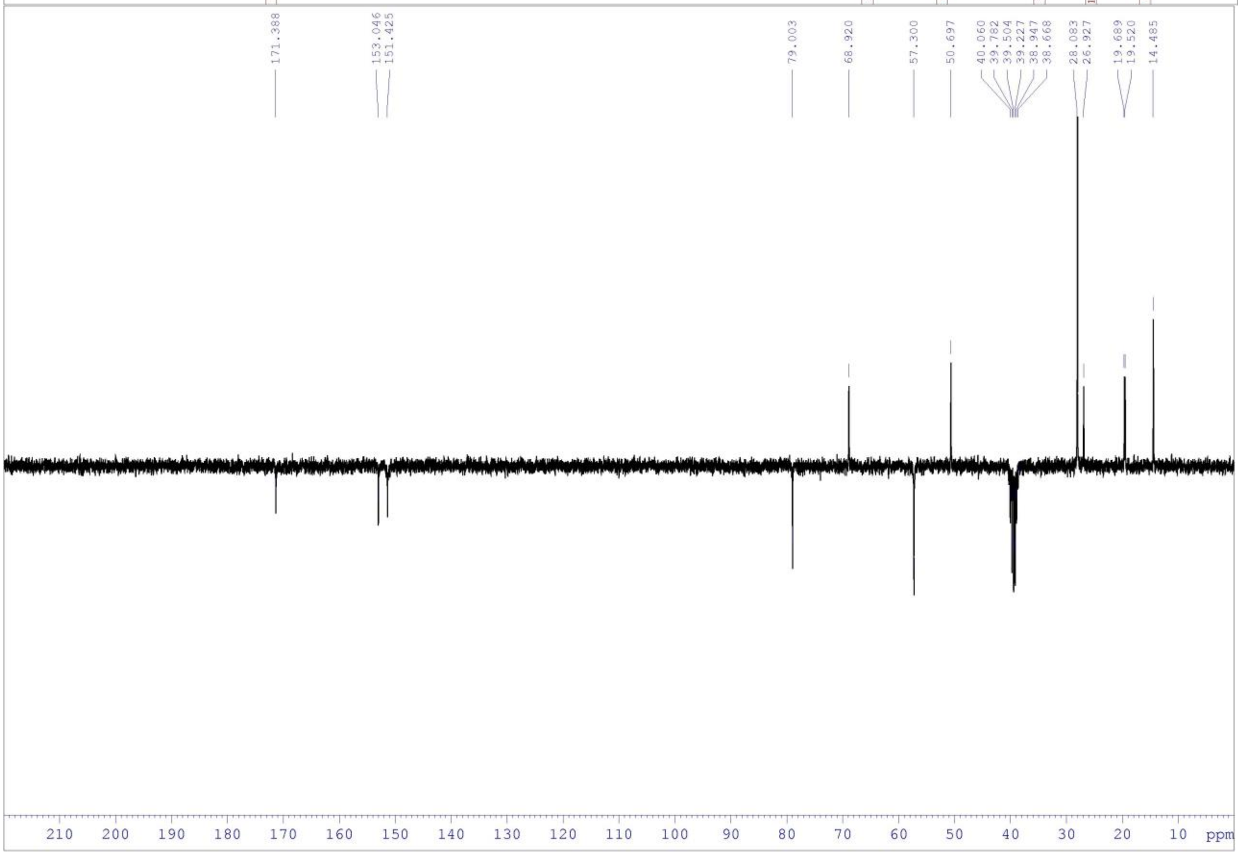
171.388
153.046
151.425
79.003
68.920
57.300
50.697
40.060
39.782
39.504
39.227
38.947
38.668
28.083
26.927
19.689
19.520
14.485
210 200 190 180 170 160 150 140 130 120 110 100 90 80 70 60 50 40 30 20 10 ppm

Compound **5**

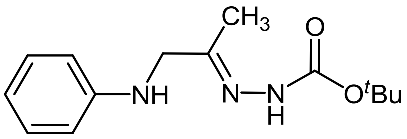

White solid, m.p. 150-156 °C, mixture of *E*- and *Z*-**5** in ratio 1:1.

^{1}H NMR (300 MHz, DMSO-d$_6$): δ = 1.45 and 1.47 (2 s, 9 H, 3 C*H$_3$* (^{t}Bu)), 1.75 and 1.81 (2 s, 3 H, C*H$_3$*), 3.73 and 4.05 (s and d, *J* = 5.9, 2 H, C*H$_2$*), 5.89, 6.56, 6.77 and 7.10 (4 m, 5 H, *Ph*), 9.52 (s, 1 H, N*H*).

^{13}C NMR (75 MHz, DMSO-d$_6$): δ = 13.7 and 1.9 (*C*H$_3$), 28.1 (*C*H$_3$ (^{t}Bu)), 49.6 and 57.4 (*C*H$_2$), 78.9 (*C* (^{t}Bu)), 112.1, 112.7, 115.9, 116.4, 128.8 and 128.9 (*Ph*), 148.5, 148.6 and 153.0 (Ph (*C*-N), *C*=N and *C*=O).

HRMS: Calcd for $C_{14}H_{22}N_3O_2$ [MH$^+$] m/z: 264.1707. Found: 264.1712.

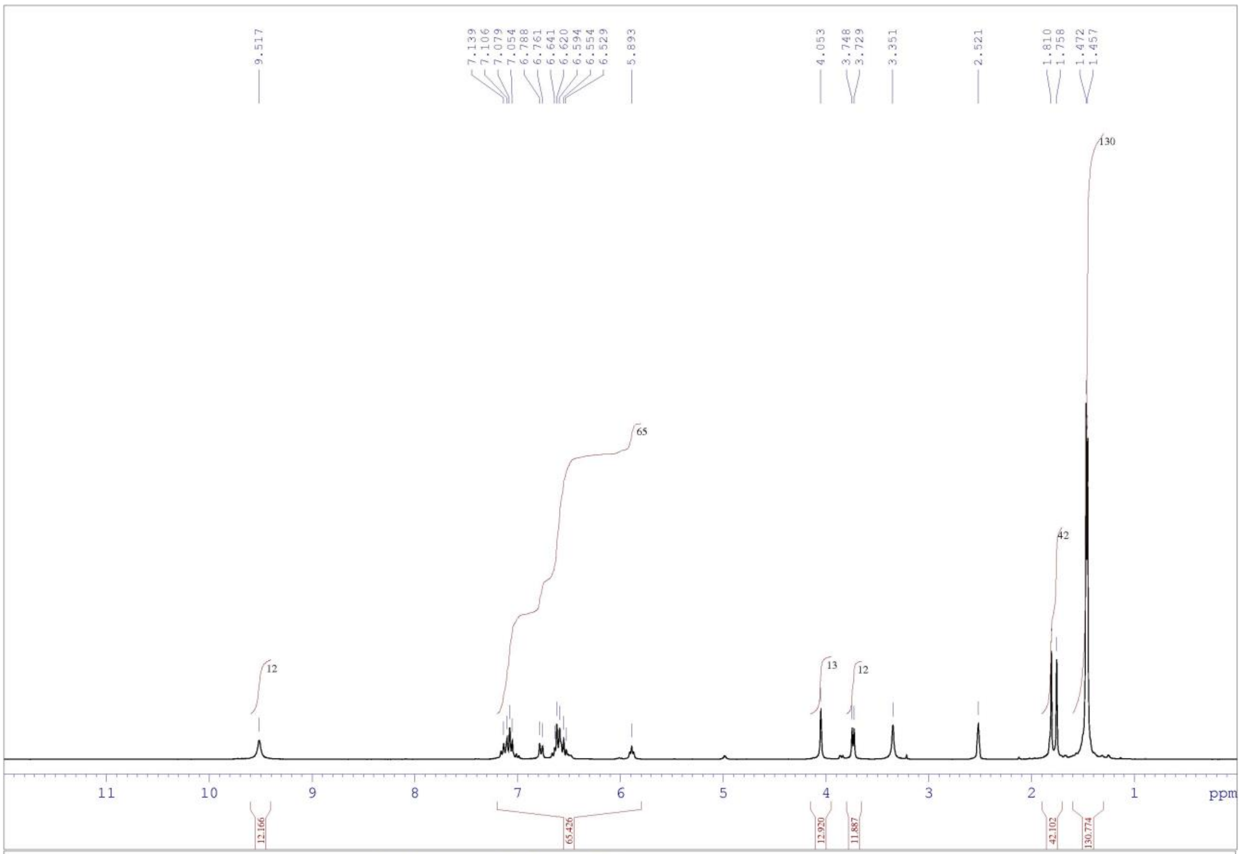
9.517
7.139
7.106
7.079
7.054
6.788
6.761
6.641
6.620
6.594
6.554
6.529
5.893
4.053
3.748
3.729
3.351
2.521
1.810
1.758
1.472
1.457
12
65
13
12
42
130
11 10 9 8 7 6 5 4 3 2 1 ppm
12.166
65.426
12.920
11.887
42.102
130.774

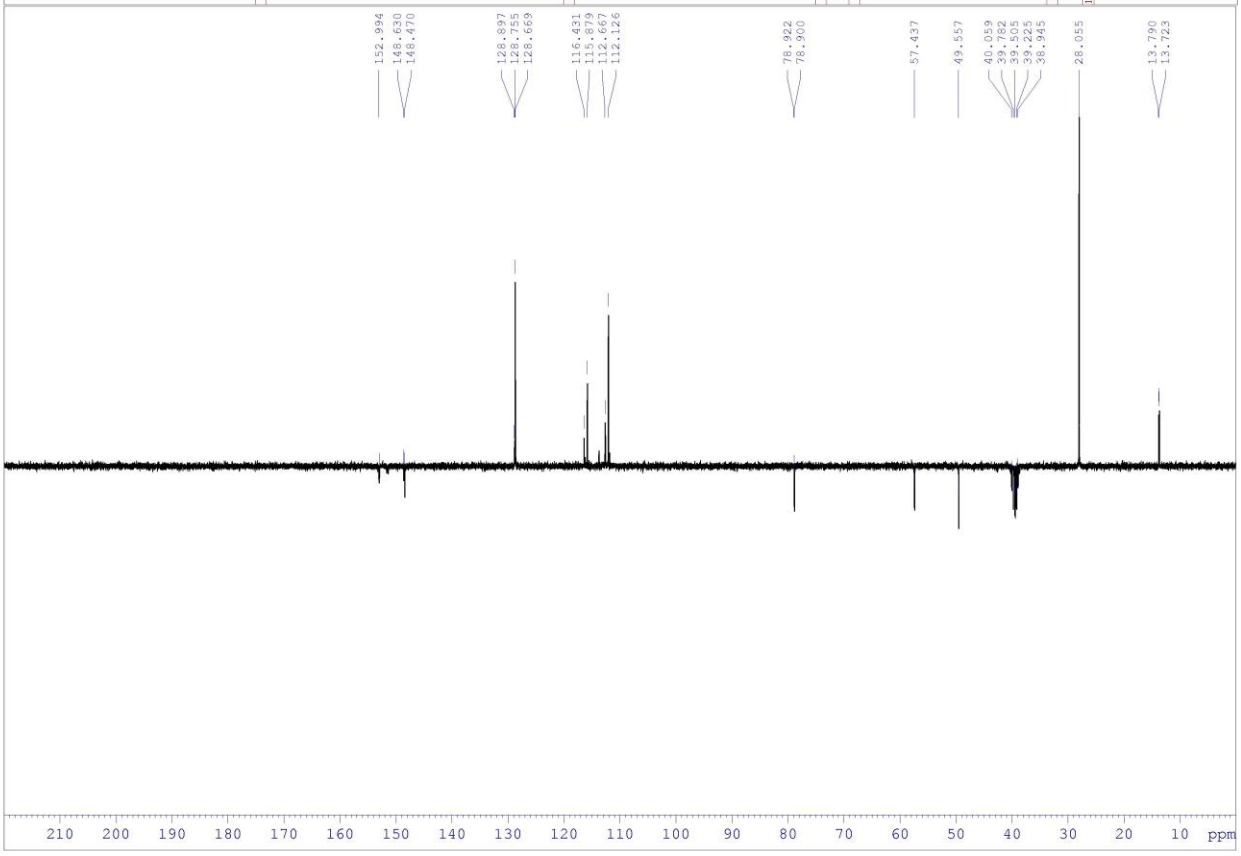
152.994
148.630
148.470
128.897
128.755
128.669
116.431
115.879
112.667
112.126
78.922
78.900
57.437
49.557
40.059
39.782
39.505
39.225
38.945
28.055
13.790
13.723
210 200 190 180 170 160 150 140 130 120 110 100 90 80 70 60 50 40 30 20 10 ppm

Compound **6**

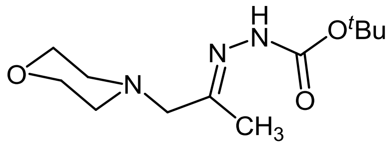

White solid, m.p. 186-189 °C, *E*-**6**.

^{1}H NMR (300 MHz, DMSO-d$_6$): δ = 1.45 (s, 9 H, 3 C*H$_3$* (^{t}Bu)), 1.84 (s, 3H, C*H$_3$*), 2.30 (s, 4 H, (C*H$_2$*)$_2$N), 2.96 (s, 2 H, C*H$_2$*), 3.98 (s, 4 H, (C*H$_2$*)$_2$O), 9.48 (s, 1 H, N*H*).

^{13}C NMR (75 MHz, DMSO-d$_6$): δ = 14.3 (*C*H$_3$), 28.0 (3 *C*H$_3$ (^{t}Bu)), 53.1 ((*C*H$_2$)$_2$N), 64.9 (*C*H$_2$), 66.1 ((*C*H$_2$)$_2$O), 78.9 (*C*, (^{t}Bu)), 151.0 and 153.0 (*C*=O and *C*=O).

HRMS: Calcd for $C_{12}H_{24}N_3O_3$ [MH$^+$] m/z: 258.1812. Found: 258.1829.

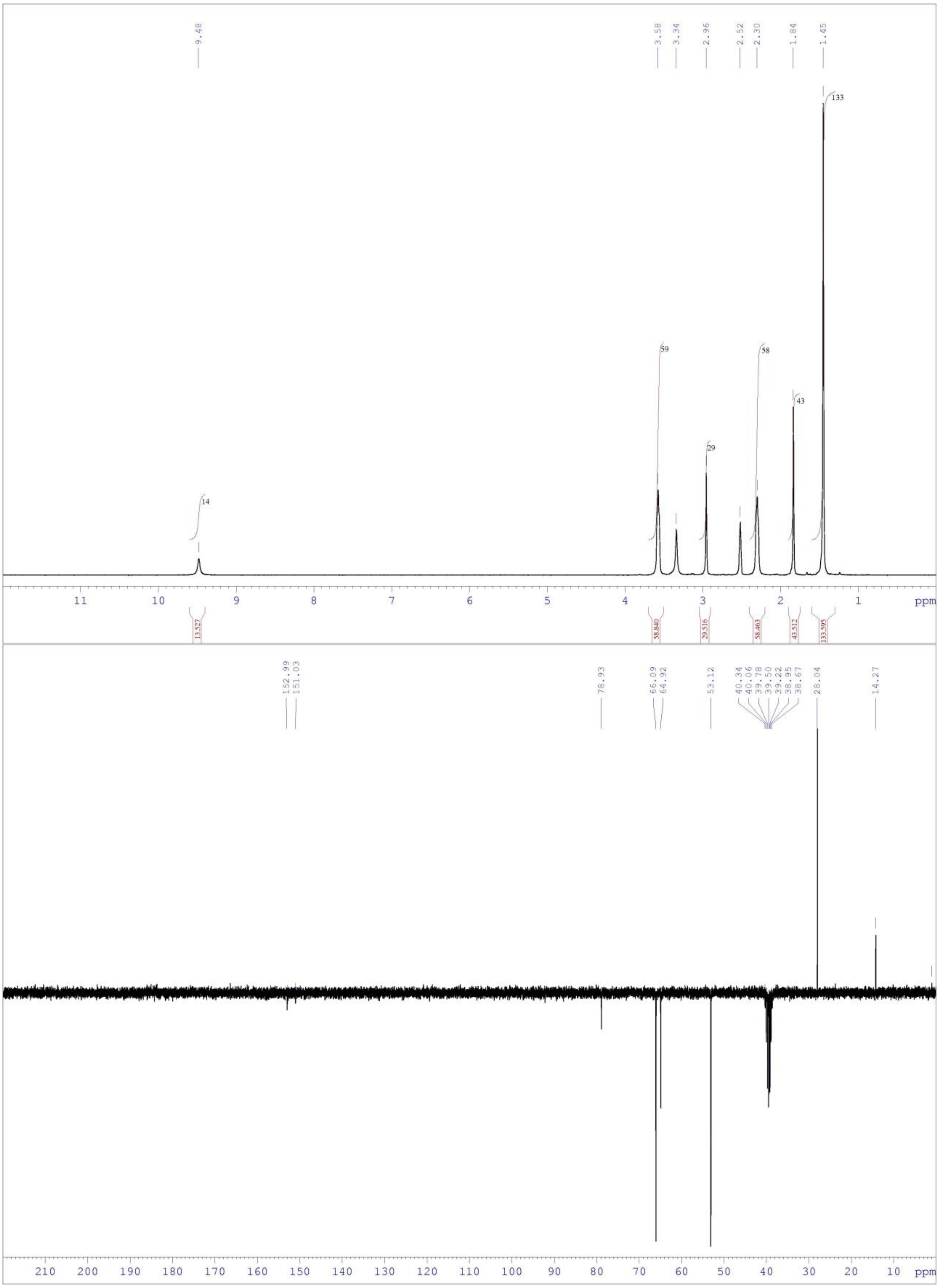
9.48
3.58
3.34
2.96
2.52
2.30
1.84
1.45
ppm
152.99
151.03
78.93
66.09
64.92
53.12
40.34
40.06
39.78
39.50
39.22
38.95
38.67
28.04
14.27
ppm

Compound **7**

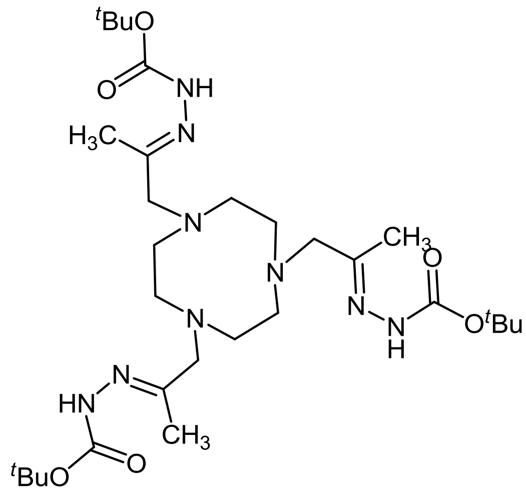

White solid, softening at 225 °C, decomposition at 240-255 °C, *E*,*E*,*E*-**7**.

^{1}H NMR (200 MHz, $CDCl_3$): δ = 1.50 (s, 27 H, 9 CH_3 (^{t}Bu)), 1.86 (s, 9 H, 3 CH_3), 2.70 (s, 12 H, 3 CH_2CH_2), 3.20 (s, 6 H, 3 CH_2), 7.49 (s, 3 H, 3 N*H*).

^{13}C NMR (50 MHz, $CDCl_3$): δ = 13.6 (3 *C*H_3), 28.4 (9 *C*H_3 (^{t}Bu)), 55.7 (3 *C*H_2*C*H_2), 65.5 (3 *C*H_2), 81.1 (3 *C* (^{t}Bu)), 151.6 and 152.8 (3 *C*=N and 3 *C*=O).

HRMS: Calcd for $C_{30}H_{58}N_9O_6$ [MH^+] m/z: 640.4505. Found: 640.4510.

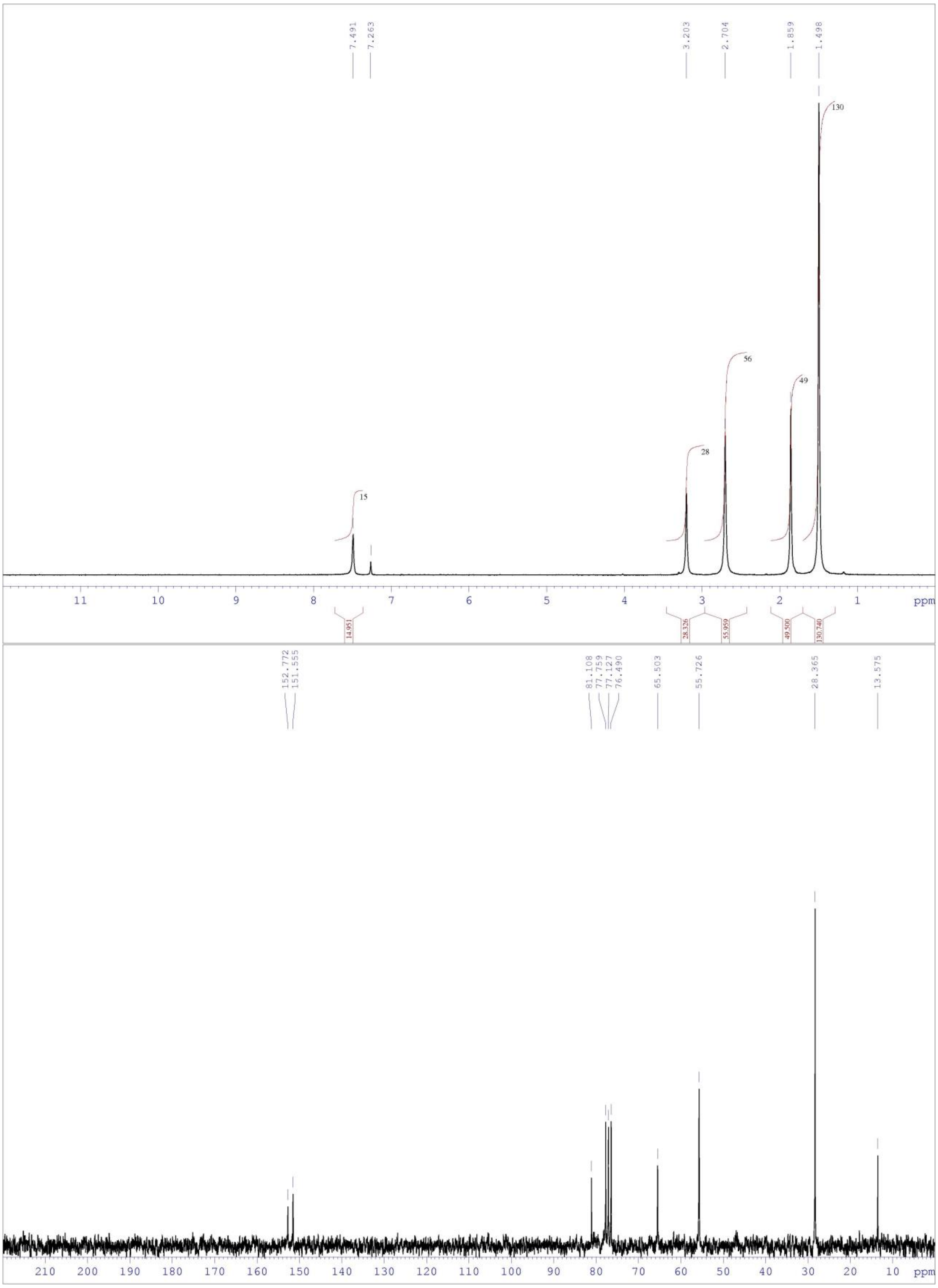
7.491
7.263
3.203
2.704
1.859
1.498
130
56
49
28
15
11 10 9 8 7 6 5 4 3 2 1 ppm
14.951
28.326
55.959
49.500
130.740
152.772
151.555
81.108
77.759
77.127
76.490
65.503
55.726
28.365
13.575
210 200 190 180 170 160 150 140 130 120 110 100 90 80 70 60 50 40 30 20 10 ppm

Compound **8**

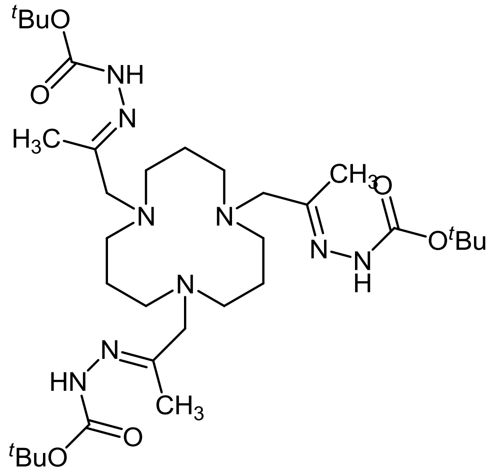

White solid, softening at 180 °C, decomposition at 240-250 °C, *E,E,E*-**8**

^{1}H NMR (200 MHz, $CDCl_3$): δ = 1.50 (s, 27 H, 9 C*H_3* (^{t}Bu)), 1.77 (m, 6 H, 3 CH_2C*H_2*CH_2), 1.84 (s, 9 H, 3 C*H_3*), 2.43 (m, 12 H, 3 C*H_2*CH_2C*H_2*), 3.08 (s, 6 H, 3 C*H_2*), 7.50 (s, 3 H, 3 N*H*).

^{13}C NMR (50 MHz, $CDCl_3$): δ = 13.6 (3 *C*H_3), 21.3 (3 CH_2*C*$H_2$$CH_2$), 28.4 (9 *C*$H_3$ (^{t}Bu)), 49.4 (3 *C*$H_2$$CH_2$*C*$H_2$), 61.6 (3 *C*$H_2$), 81.2 (3 *C* (^{t}Bu)), 151.5 and 152.8 (3 *C*=N and 3 *C*=O).

HRMS: Calcd for $C_{33}H_{64}N_9O_6$ [MH^+] m/z: 682.4974. Found: 682.4983

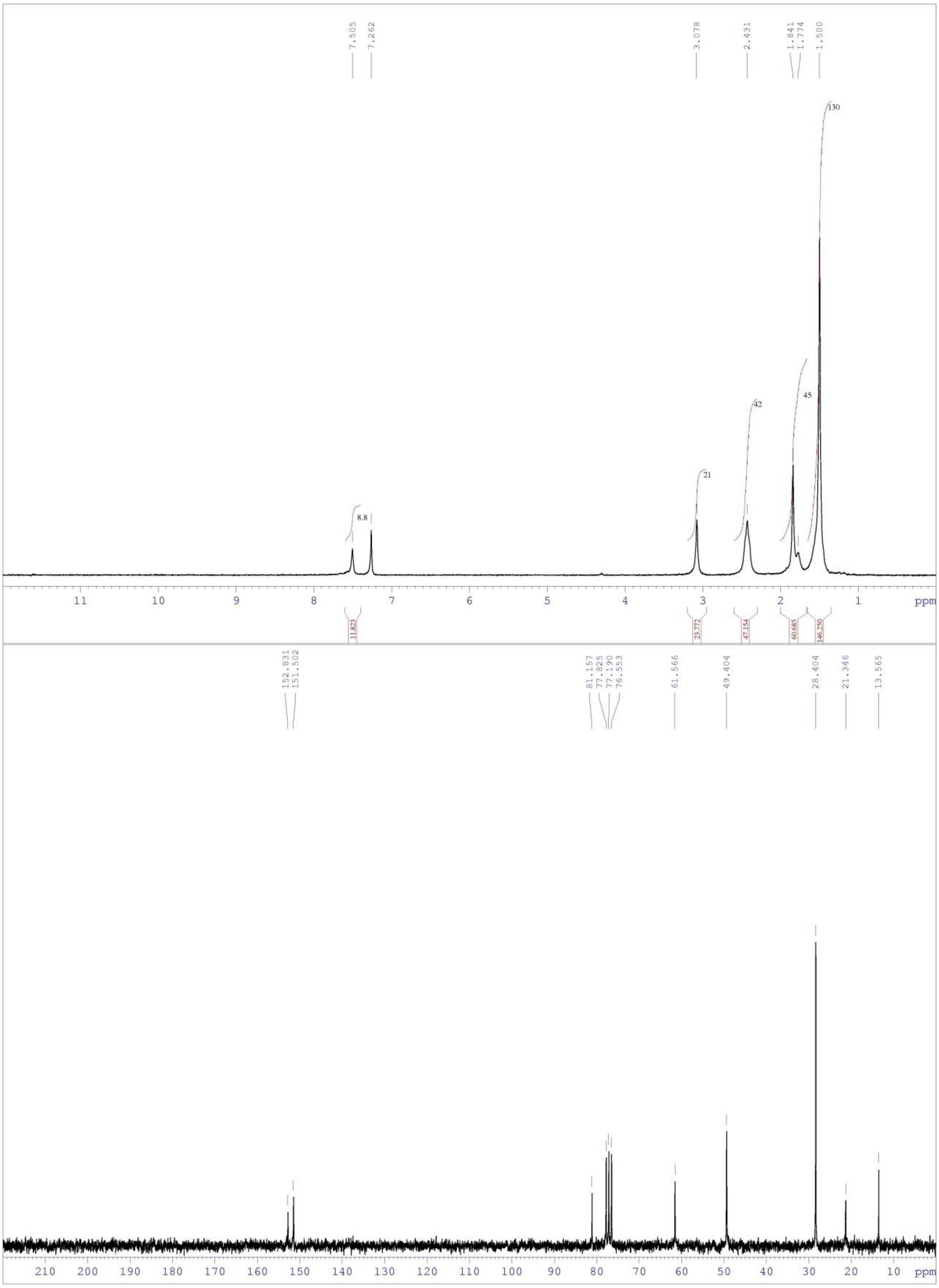
7.505
7.262
3.078
2.431
1.841
1.774
1.500
130
45
42
21
8.8
11 10 9 8 7 6 5 4 3 2 1 ppm
11.823
23.772
47.154
60.685
146.250
152.831
151.502
81.157
77.825
77.190
76.553
61.566
49.404
28.404
21.346
13.565
210 200 190 180 170 160 150 140 130 120 110 100 90 80 70 60 50 40 30 20 10 ppm

Compound **9**

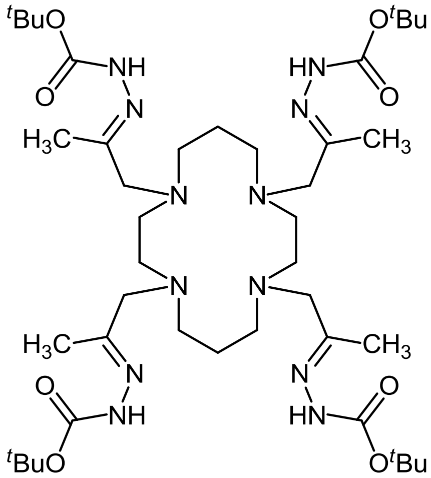

White solid, softening at 239 °C, decomposition at 245-250 °C, *E.E.E.E*-**7**.

^{1}H NMR (200 MHz, $CDCl_3$): δ = 1.50 (s, 36 H, 12 C*H$_3$* (^{t}Bu)), 1.82 (s, 12 H, 4 C*H$_3$*), 1.86 (m, 4 H, 2 CH_2C*H$_2$*CH_2), 2.42 and 2.51 (2 m, 16 H, 2 C*H$_2$*C*H$_2$* and 2 C*H$_2$*CH_2C*H$_2$*), 3.07 (s, 8 H, 4 C*H$_2$*), 7.54 (s, 4 H, 4 N*H*).

^{13}C NMR (50 MHz, $CDCl_3$): δ = 13.3 (4 *C*H_3), 22.7 (2 CH_2*C*H_2CH_2), 28.4 (12 *C*H_3 (^{t}Bu)), 50.4 and 51.5 (2 *C*H_2*C*H_2 and 2 *C*H_2CH_2*C*H_2), 61.7 (4 *C*H_2), 81.1 (4 *C* (^{t}Bu)), 151.4 and 152.8 (4 *C*=N and 4 *C*=O).

HRMS: Calcd for $C_{42}H_{81}N_{12}O_8$ [MH^+] m/z: 881.6295. Found: 881.6302.

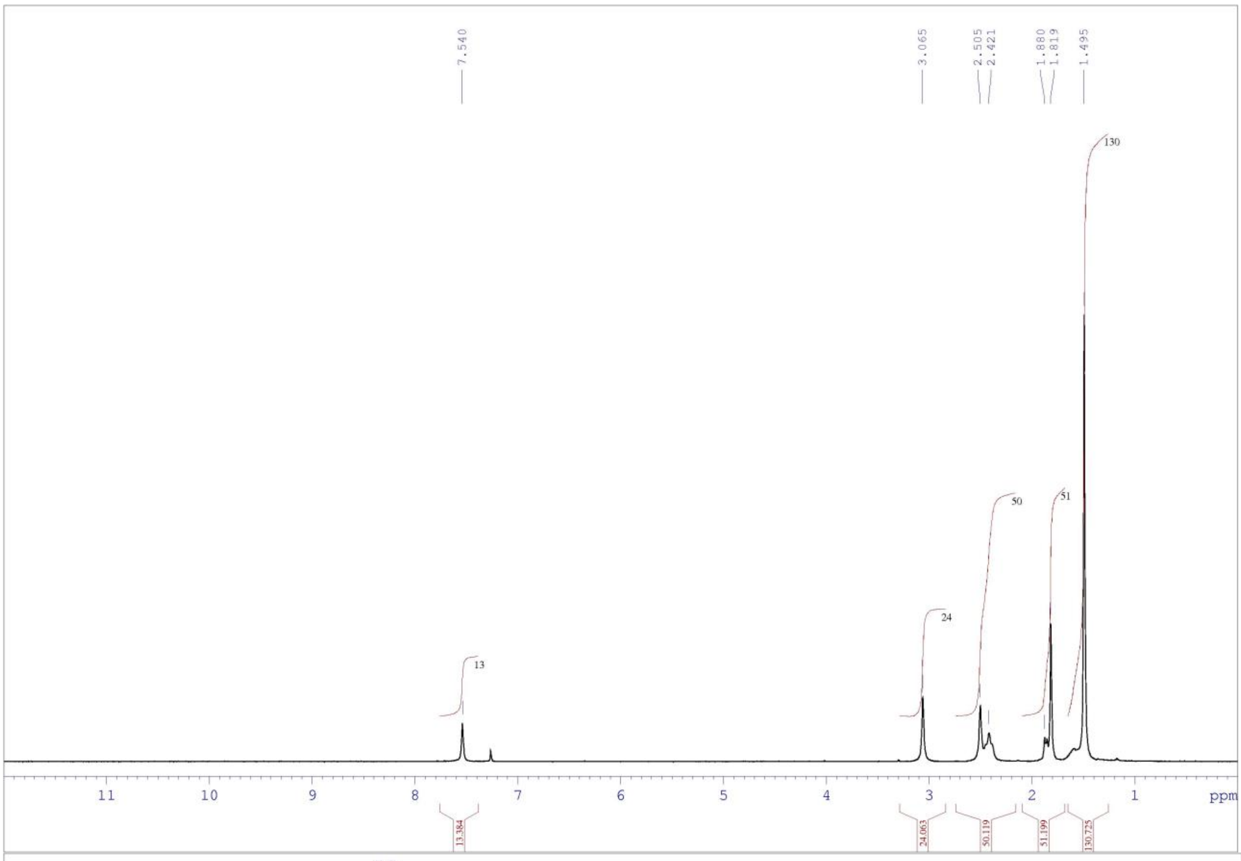
7.540
3.065
2.505
2.421
1.880
1.819
1.495
130
50
51
24
13
11
10
9
8
7
6
5
4
3
2
1
ppm
13.384
24.063
50.119
51.199
130.725

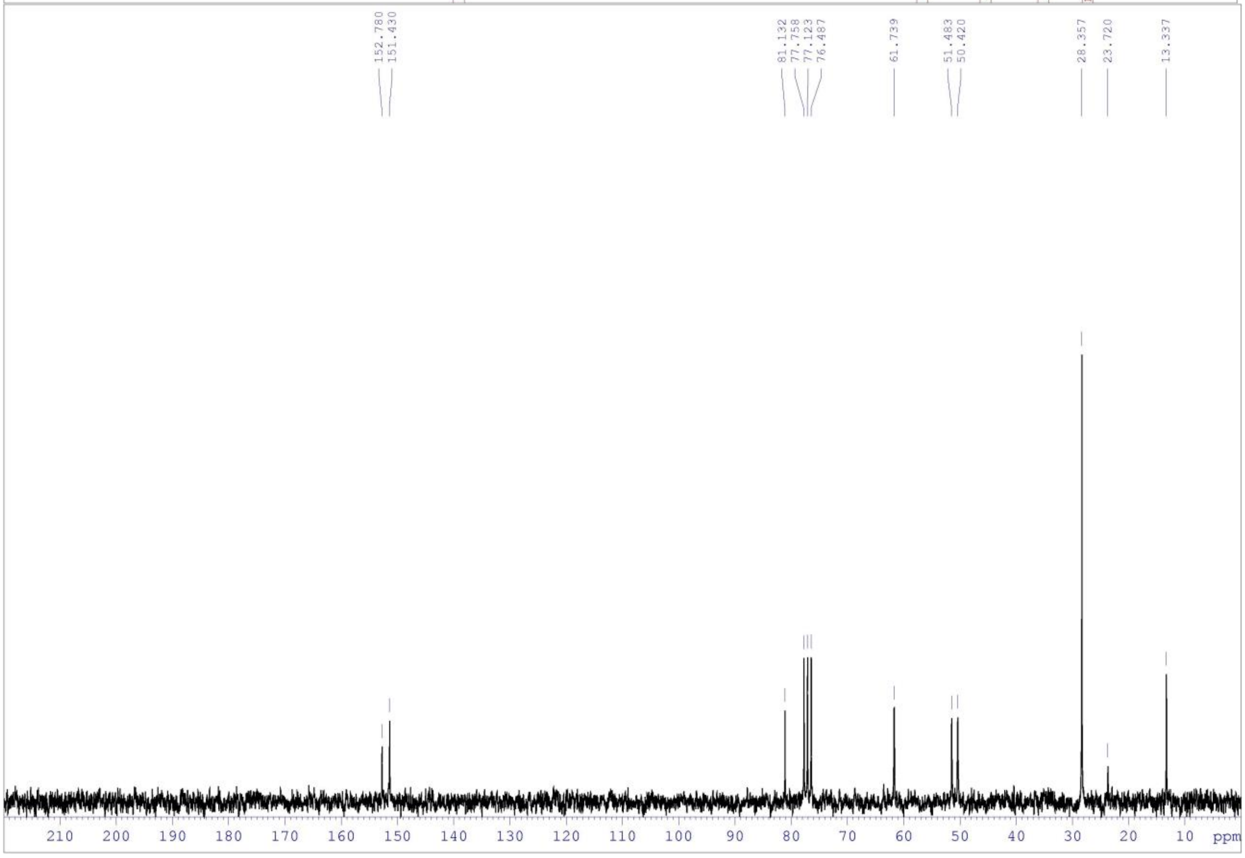
152.780
151.430
81.132
77.758
77.123
76.487
61.739
51.483
50.420
28.357
23.720
13.337
210
200
190
180
170
160
150
140
130
120
110
100
90
80
70
60
50
40
30
20
10
ppm

Compound **10**

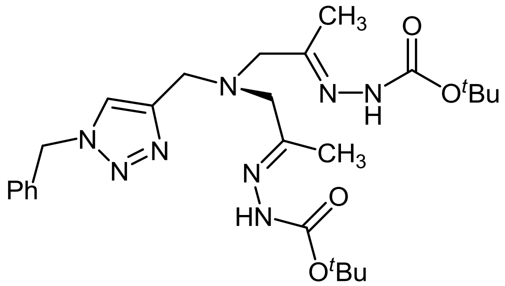

White solid, m.p. 140-144 °C, mixture of isomers with ratio of *E*- and *Z*-fragments 4.8:1.

^{1}H NMR (300 MHz, DMSO-d$_6$): main isomer, δ = 1.44 (s, 18 H, 6 C*H$_3$* (^{t}Bu)), 1.80 (s, 6 H, 2 C*H$_3$*), 2.99 (s, 4 H, 2 C*H$_2$*), 3.59 (s, 2 H, C*H$_2$*), 5.60 (s, 2 H, C*H$_2$*Ph), 7.2-7.4 (m, 5 H, *Ph*), 8.13 (s, 1 H, *Tz*), 9.47 (s, 2 H, 2 N*H*); selected signals of minor isomers, δ = 1.89 (s), 3.05 and 3.17 (2 s), 9.59 and 10.91 (2 s).

^{13}C NMR (75 MHz, DMSO-d$_6$): main isomer, δ = 14.4 (2 *C*H$_3$), 28.1 (6 *C*H$_3$ (^{t}Bu)), 47.6 and 52.8 (*C*H$_2$ and *C*H$_2$Ph), 59.7 (2 *C*H$_2$), 79.0 (2 *C* (^{t}Bu)), 124.3 and 143.3 (*Tz*), 127.7, 128.0, 128.7 and 136.2 (*Ph*), 151.6 and 153.1 (2 *C*=N and 2 *C*=O); selected signals of minor isomers, δ = 14.6 and 23.4, 47.2, 60.2, 79.1, 124.5, 127.6, 136.0.

HRMS: Calcd for $C_{26}H_{41}N_8O_4$ [MH$^+$] m/z: 529.3245. Found: 529.3238.

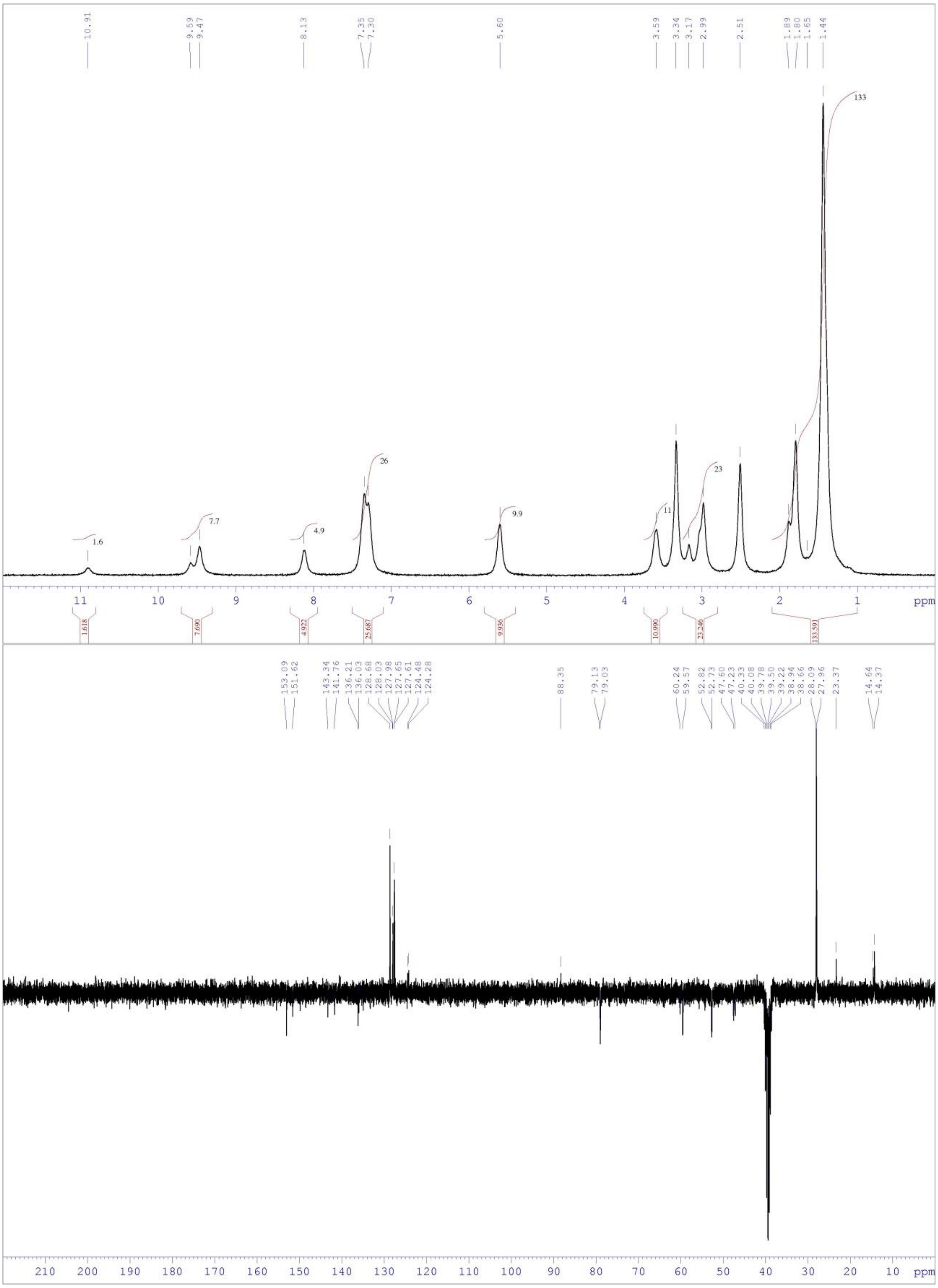

10.91
9.59
9.47
8.13
7.35
7.30
5.60
3.59
3.34
3.17
2.99
2.51
1.89
1.80
1.65
1.44
133
26
23
11
9.9
7.7
4.9
1.6
ppm
1.618
7.690
4.922
25.687
9.936
10.990
23.246
133.591
153.09
151.62
143.34
141.76
136.21
136.03
128.68
128.03
127.98
127.65
127.61
124.48
124.28
88.35
79.13
79.03
60.24
59.57
52.82
52.73
47.60
47.23
40.33
40.08
39.78
39.50
39.22
38.94
38.66
28.09
27.96
23.37
14.64
14.37

Compound **11a**

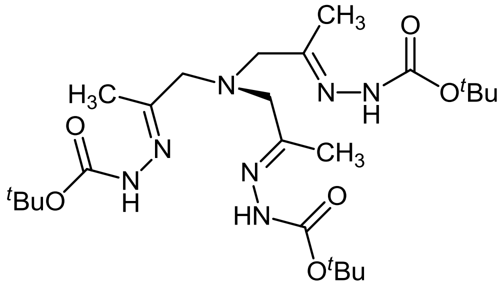

White solid, m.p. 184-187 °C, mixture of isomers with ratio of *E*- and *Z*-fragments >20:1.

^{1}H NMR (300 MHz, DMSO-d$_6$): main isomer (*E*,*E*,*E*-**11a**), δ = 1.45 (s, 27 H, 9 C*H*$_3$ (^{t}Bu)), 1.82 (s, 9 H, 3 C*H*$_3$), 2.94 (s, 6 H, 3 C*H*$_2$), 9.47 (3 N*H*); selected signals of minor isomers, δ = 1.41 (s), 1.86 (s), 3.02 (s), 3.20 (s), 9.55 (s).

^{13}C NMR (75 MHz, DMSO-d$_6$): main isomer (*E*,*E*,*E*-**11a**), δ = 14.5 (3 *C*H$_3$), 28.1 (9 *C*H$_3$ (^{t}Bu)), 60.0 (3 *C*H$_2$), 79.0 (3 *C* (^{t}Bu)), 151.4 and 153.0 (3 *C*=N and 3 *C*=O); selected signals of minor isomers, δ = 14.8, 23.5, 27.9, 61.3.

HRMS: Calcd for $C_{24}H_{46}N_7O_6$ [MH^+] m/z: 528.3504. Found: 528.3489.

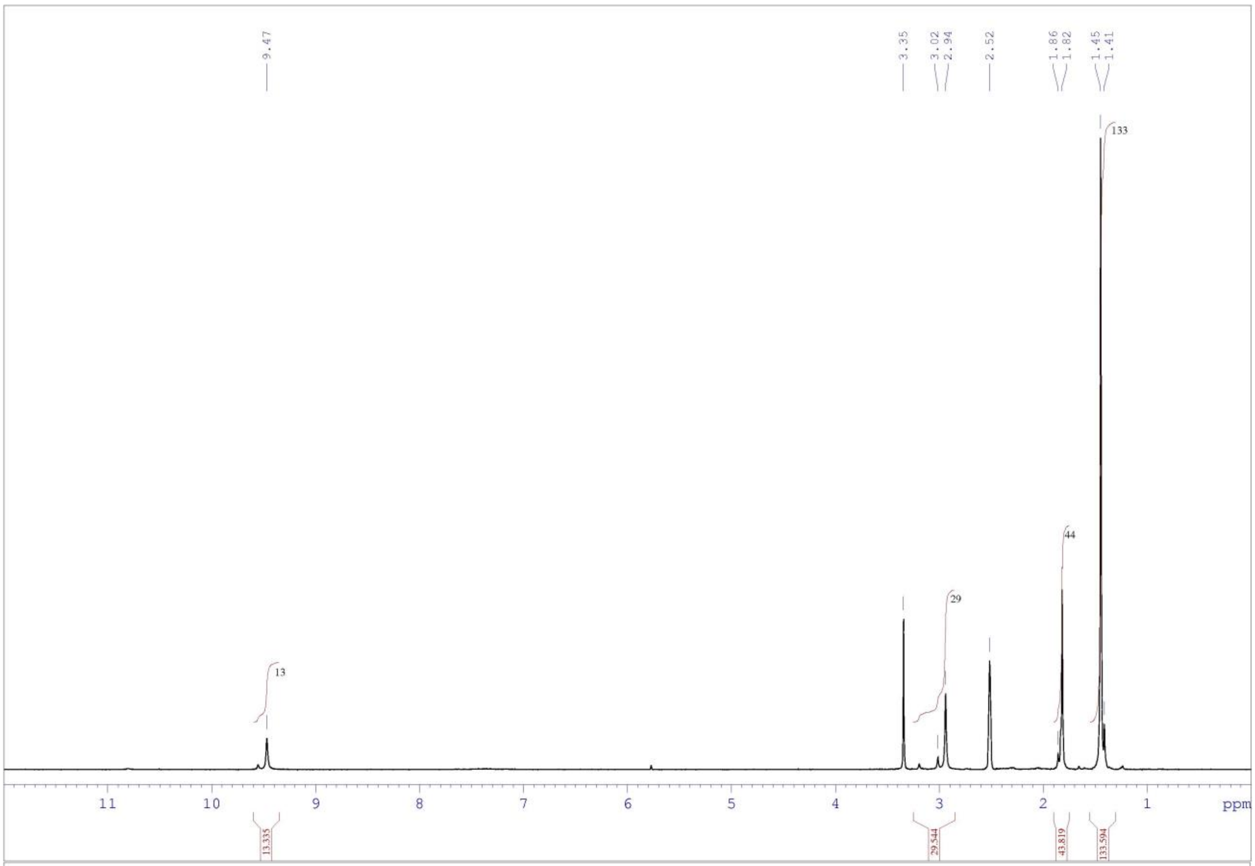
9.47
3.35
3.02
2.94
2.52
1.86
1.82
1.45
1.41
133
44
29
13
11 10 9 8 7 6 5 4 3 2 1 ppm
13.335
29.544
43.819
133.594

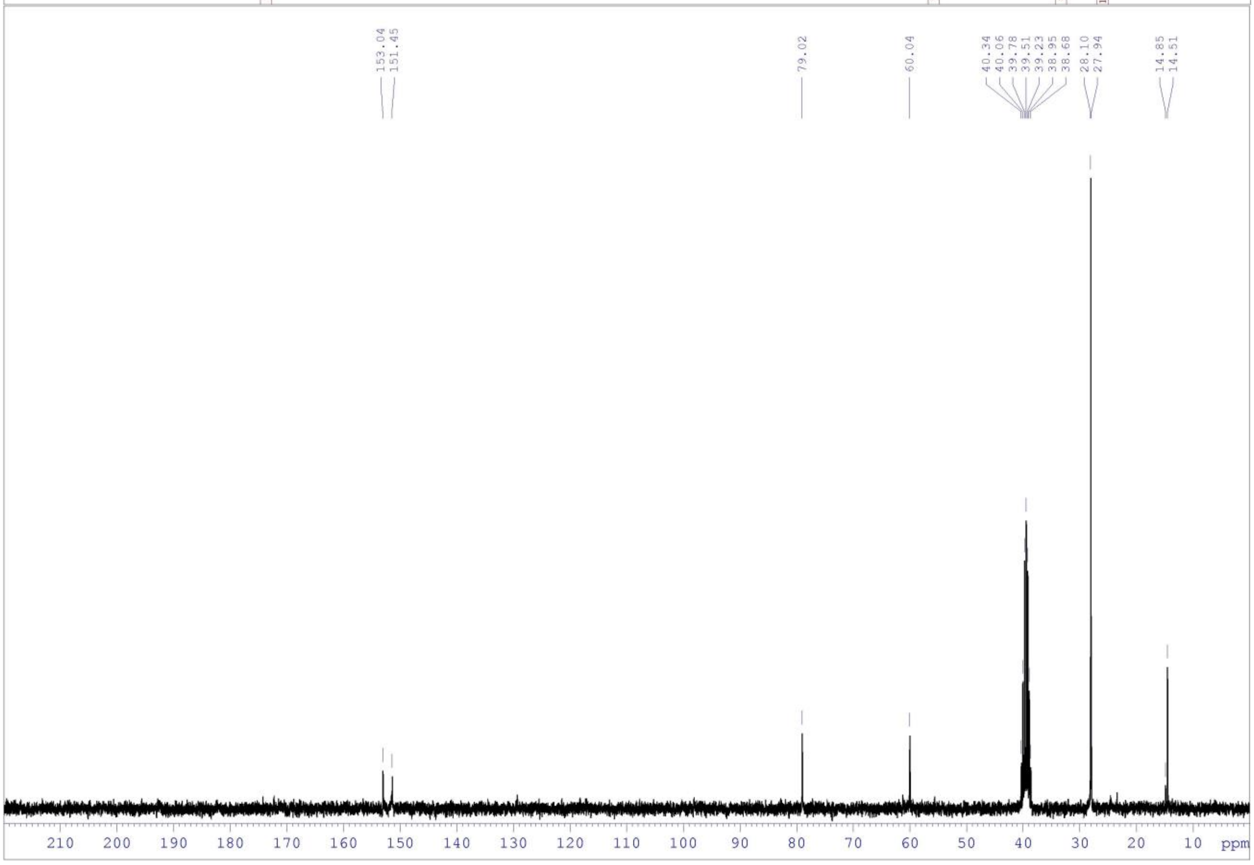
153.04
151.45
79.02
60.04
40.34
40.06
39.78
39.51
39.23
38.95
38.68
28.10
27.94
14.85
14.51
210 200 190 180 170 160 150 140 130 120 110 100 90 80 70 60 50 40 30 20 10 ppm

Compound **11b**

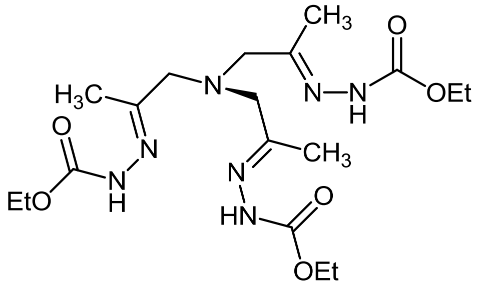

White solid, m.p. 181-185 °C, mixture of isomers with ratio of *E*- and *Z*-fragments 9:1.
^{1}H NMR (300 MHz, DMSO-d_6): main isomer (*E*,*E*,*E*-**11b**), δ = 1.23 (t, *J* = 7.0, 9 H, 3 CH_2CH_3), 1.84 (s, 9 H, 3 CH_3), 3.00 (s, 6 H, 3 CH_2), 4.12 (q, *J* = 7.0, 6 H, 3 CH_2CH_3), 9.5-9.8 (s br, 3 H, 3 N*H*); selected signals of minor isomers, δ = 1.88 (s), 3.07 (s), 3.20 (s).
^{13}C NMR (75 MHz, DMSO-d_6): main isomer (*E*,*E*,*E*-**11b**), δ = 14.5 (3 CH_3 and 3 CH_2CH_3), 60.0 and 60.2 (3 CH_2 and 3 CH_2CH_3), 151.8 and 154.1 (3 *C*=N and 3 *C*=O); selected signals of minor isomers, δ = 14.8, 23.3, 55.3, 61.1, 150.7.
Elemental analysis. For $C_{18}H_{33}N_7O_6$ calcd: C, 48.75%; H, 7.50%; N, 22.11%. Found: C, 48.85%; H, 7.63%; N, 21.89%.

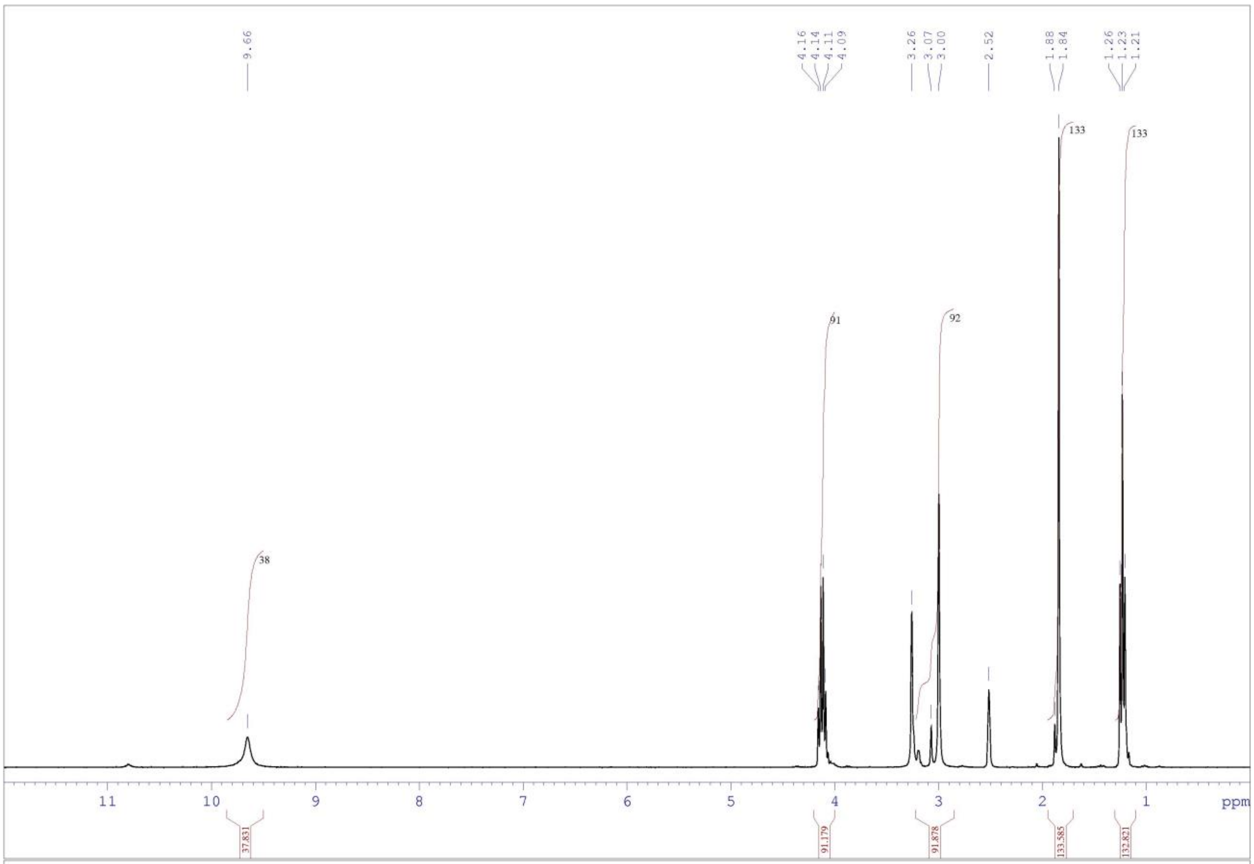
9.66
4.16
4.14
4.11
4.09
3.26
3.07
3.00
2.52
1.88
1.84
1.26
1.23
1.21
38
91
92
133
133
11 10 9 8 7 6 5 4 3 2 1 ppm
37.831
91.179
91.878
133.585
132.821

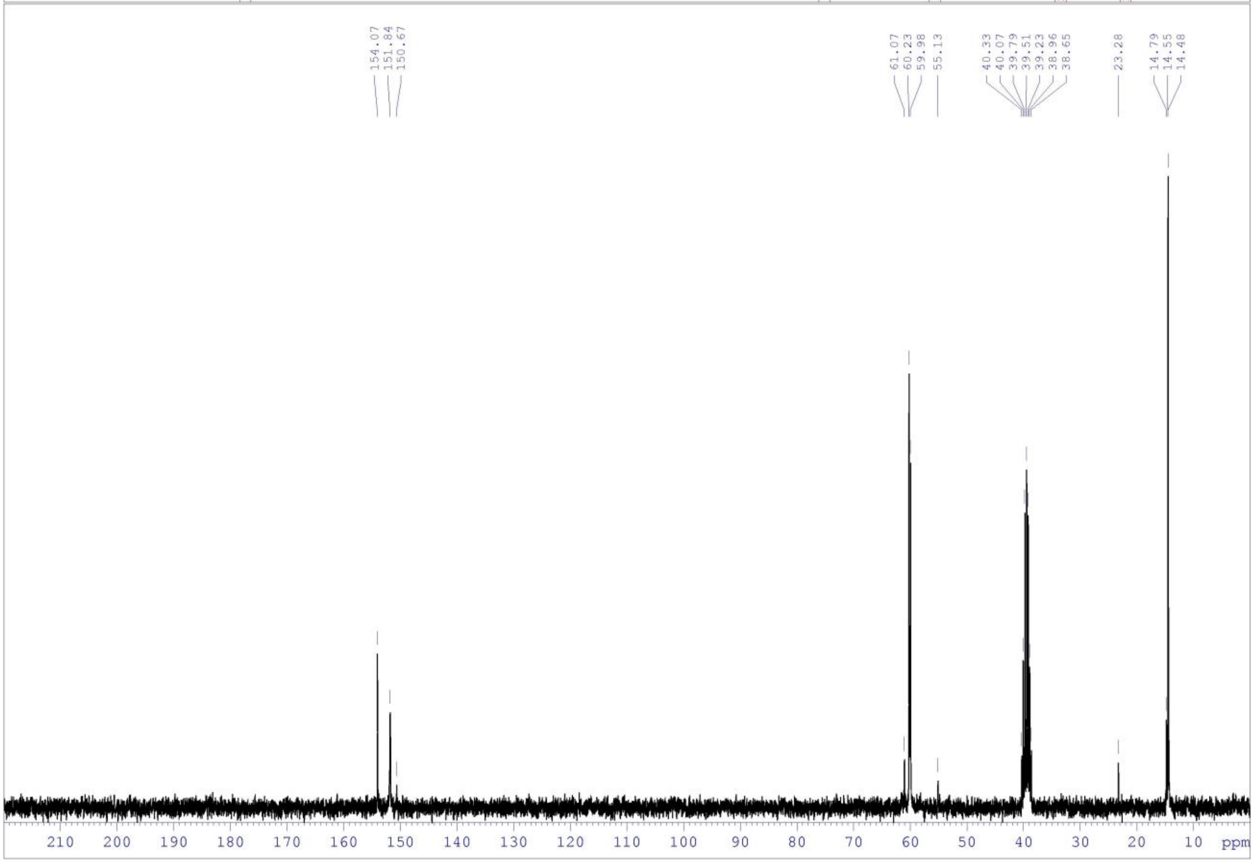
154.07
151.84
150.67
61.07
60.23
59.98
55.13
40.33
40.07
39.79
39.51
39.23
38.96
38.65
23.28
14.79
14.55
14.48
210 200 190 180 170 160 150 140 130 120 110 100 90 80 70 60 50 40 30 20 10 ppm

Compound **11d**

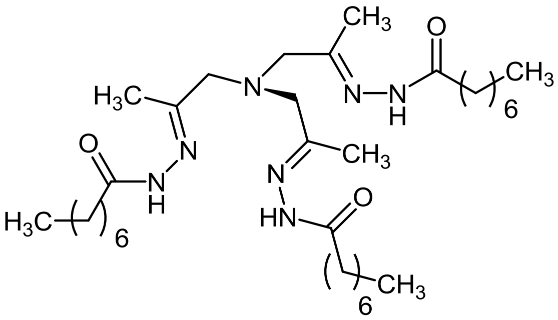

White solid, m.p. 118-124 °C, mixture of isomers with ratio of *E*- and *Z*-fragments 1:1.2.

^{1}H NMR (200 MHz, DMSO-d$_6$): δ = 0.85 (m, 9 H, 3 C*H$_3$* (Hept)), 1.25 (s, 24 H, 3 (C*H$_2$*)$_4$), 1.51 (m, 6 H, 3 C*H$_2$*(CH$_2$)$_4$), 1.83 and 1.85 (2 s, 9 H, 3 C*H$_3$*), 2.21 and 2.44 (2 m, 6 H, 3 C*H$_2$*CO), 3.05 and 3.09 (2 s, 6 H, 3 C*H$_2$*), 10.0 (s, 3 H, 2 N*H*).

^{13}C NMR (50 MHz, DMSO-d$_6$): δ = 13.9 and 14.4 (3 *C*H$_3$ and 3 *C*H$_3$ (Hept)), 22.1, 24.3, 25.1, 28.5, 28.7, 31.2 and 32.2 (3 (*C*H$_2$)$_6$), 60.7 (3 *C*H$_2$), 149.6 (3 *C*=N), 174.8 (3 *C*=N).

HRMS: Calcd for $C_{33}H_{64}N_7O_3$ [MH$^+$] m/z: 606.5065. Found: 606.5073.

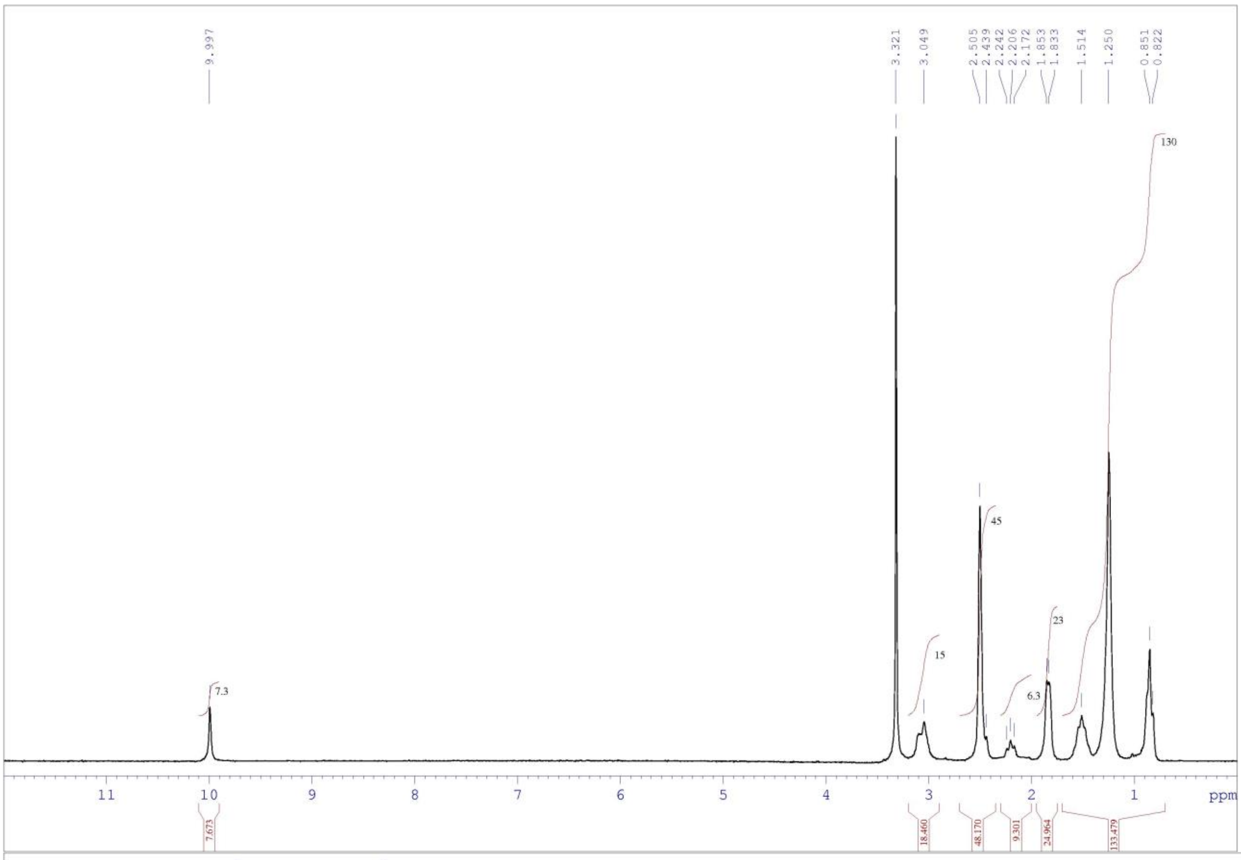
9.997
3.321
3.049
2.505
2.439
2.242
2.206
2.172
1.853
1.833
1.514
1.250
0.851
0.822
130
45
23
15
7.3
6.3
11
10
9
8
7
6
5
4
3
2
1
ppm
7.673
18.460
48.170
9.301
24.964
133.479

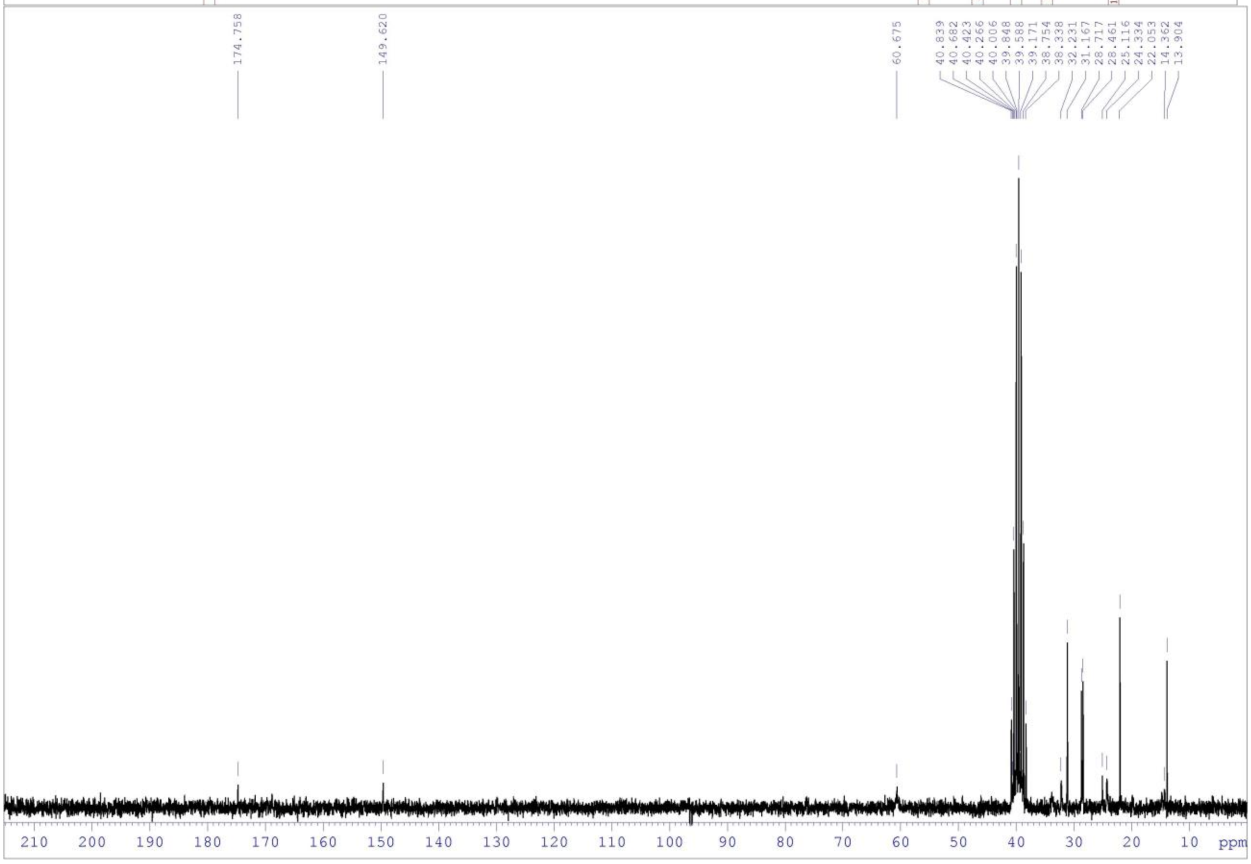
174.758
149.620
60.675
40.839
40.682
40.423
40.266
40.006
39.848
39.588
39.171
38.754
38.338
32.231
31.167
28.717
28.461
25.116
24.334
22.053
14.362
13.904
210
200
190
180
170
160
150
140
130
120
110
100
90
80
70
60
50
40
30
20
10
ppm

Compound **11h**

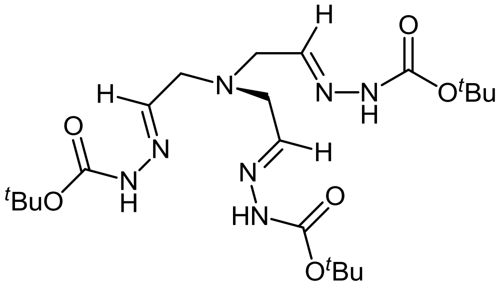

White solid, m.p. 169-172 °C (with decomposition), *E*,*E*,*E*-**11h**.
^{1}H NMR (200 MHz, DMSO-d$_6$): δ = 1.42 (s, 27 H, 9 C*H*$_3$ (^{t}Bu)), 3.13 (d, *J* = 4.4, 6 H, 3 C*H*$_2$), 7.27 (m, 3 H, 3 C*H*), 10.54 (s, 3 H, 3 N*H*).
^{13}C NMR (50 MHz, DMSO-d$_6$): δ = 28.1 (9 *C*H$_3$ (^{t}Bu)), 54.7 (3 *C*H$_2$), 79.2 (3 *C* (^{t}Bu)), 144.1 (3 *C*=N), 152.4 (3 *C*=O).
HRMS: Calcd for $C_{21}H_{40}N_7O_6$ [MH$^+$] m/z: 486.3035. Found: 486.3035.

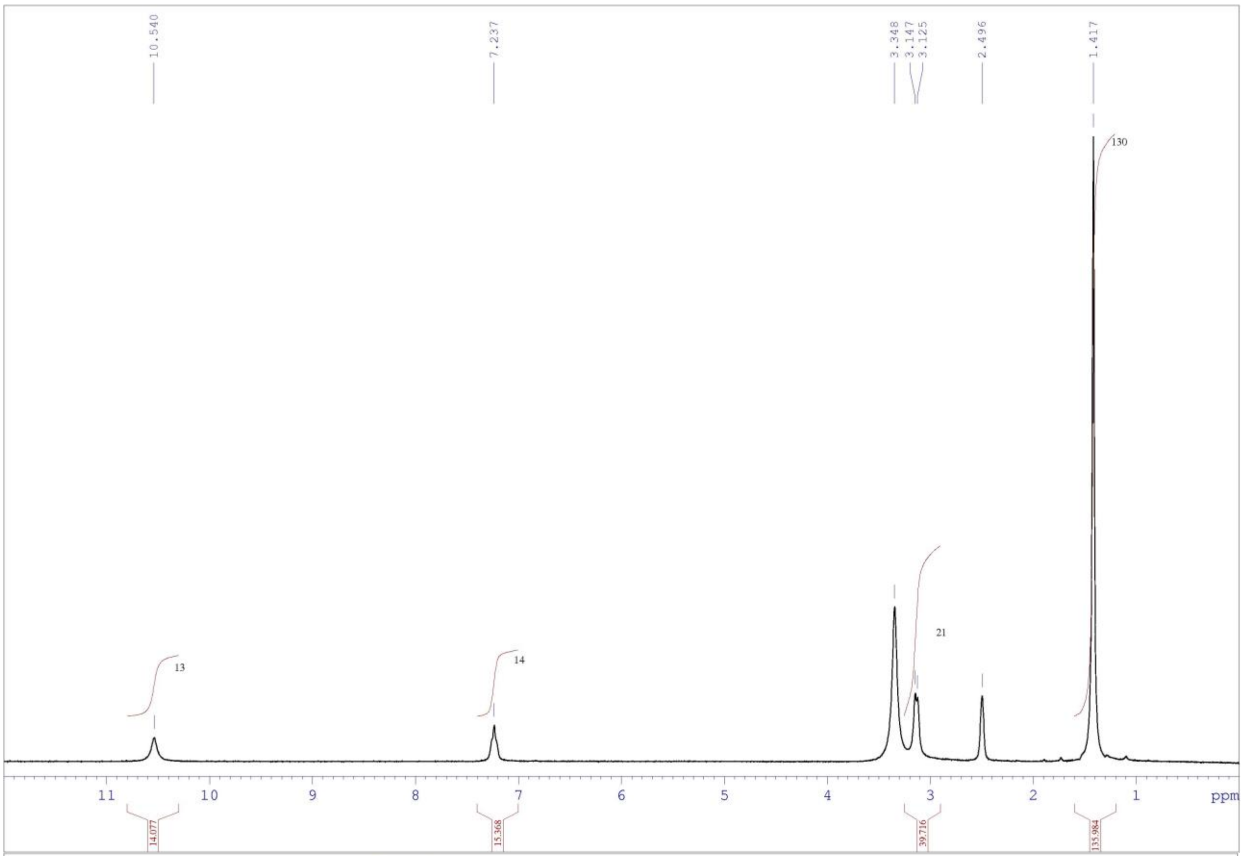
10.540
7.237
3.348
3.147
3.125
2.496
1.417
130
21
13
14
11
10
9
8
7
6
5
4
3
2
1
ppm
14.077
15.368
39.716
135.984
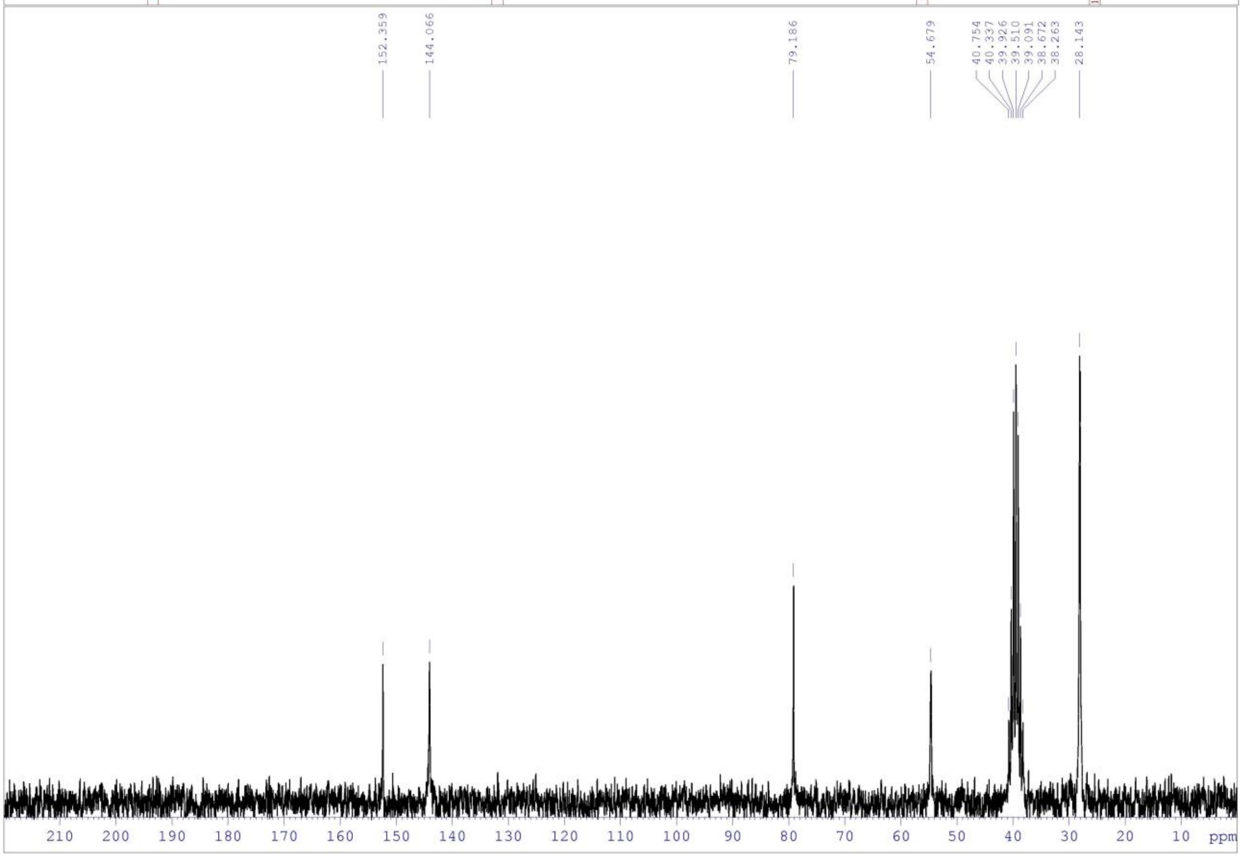
152.359
144.066
79.186
54.679
40.754
40.337
39.926
39.510
39.091
38.672
38.263
28.143
210
200
190
180
170
160
150
140
130
120
110
100
90
80
70
60
50
40
30
20
10
ppm

Compound **11f**

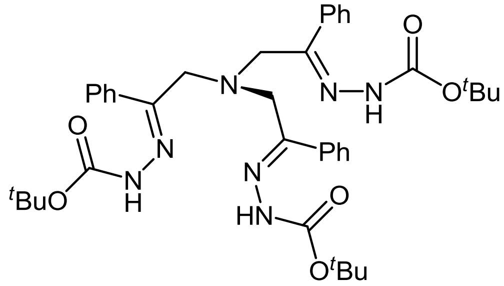

Pale yellow foam, softening at 77 °C, melting at 103 °C, mixture of isomers.
^{1}H NMR (200 MHz, DMSO-d$_6$): δ = 1.39, 1.44 and 1.48 (3 s, 27 H, 9 C*H$_3$* (^{t}Bu)), 3.61, 3.88 and 3.89 (3 s, 6 H, 3 C*H$_2$*), 7.1-7.8 (m, 15 H, 3 *Ph*), 8.68, 8.90, 10.0 and 10.1 (4 s, 3 H, 3 N*H*).
^{13}C NMR (50 MHz, DMSO-d$_6$): δ = 28.0 (9 *C*H$_3$ (^{t}Bu)), 58.9 (3 *C*H$_2$), 79.2 and 79.8 (3 *C* (^{t}Bu)), 126.3, 127.2, 128.0, 128.2, 128.5, 128.9, 129.2, 132.3 and 137.5 (3 *Ph*), 145.6, 150.6, 152.1 and 152.3 (3 *C*=N and 3 *C*=O).
HRMS: Calcd for $C_{39}H_{52}N_7O_6$ [MH$^+$] m/z: 714.3974. Found: 714.3955.

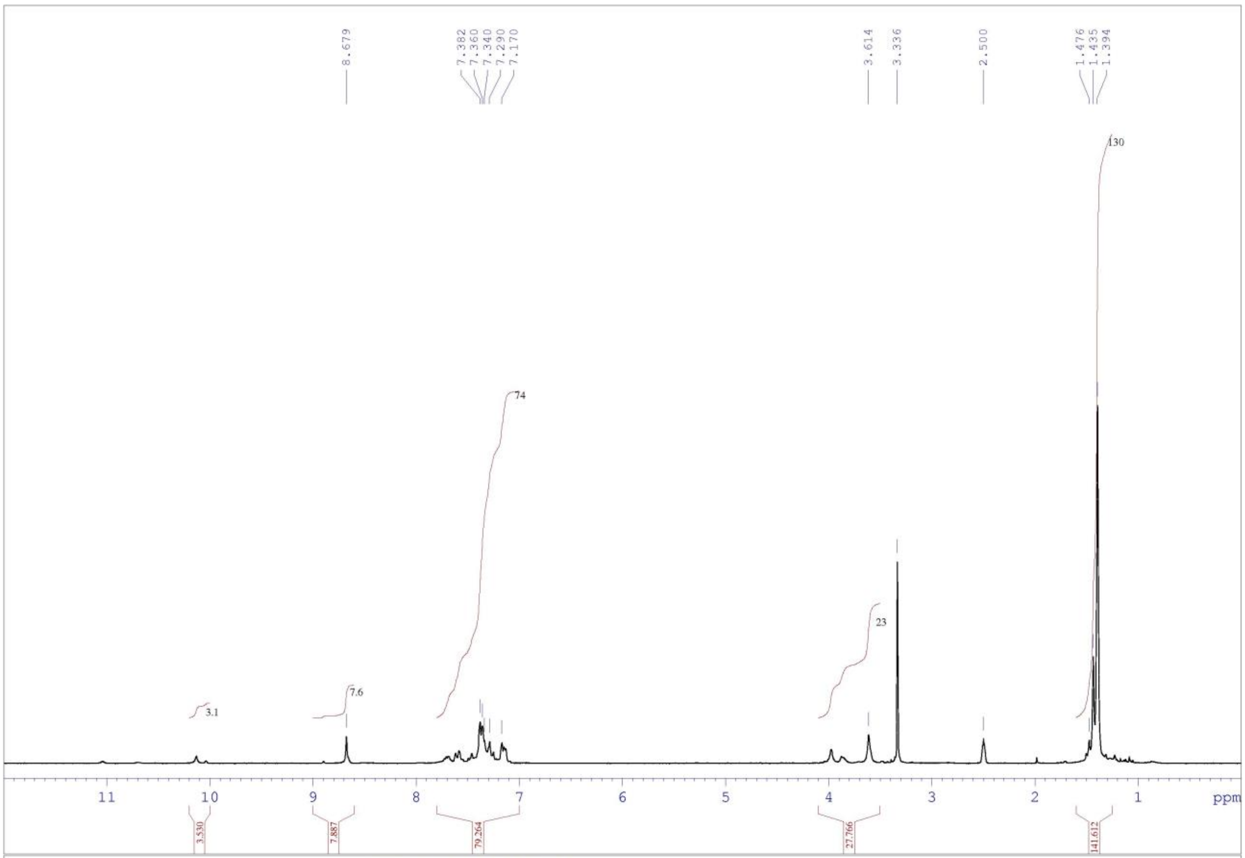
8.679
7.382
7.360
7.340
7.290
7.170
3.614
3.336
2.500
1.476
1.435
1.394
130
74
23
7.6
3.1
3.530
7.887
79.264
27.766
141.612
ppm

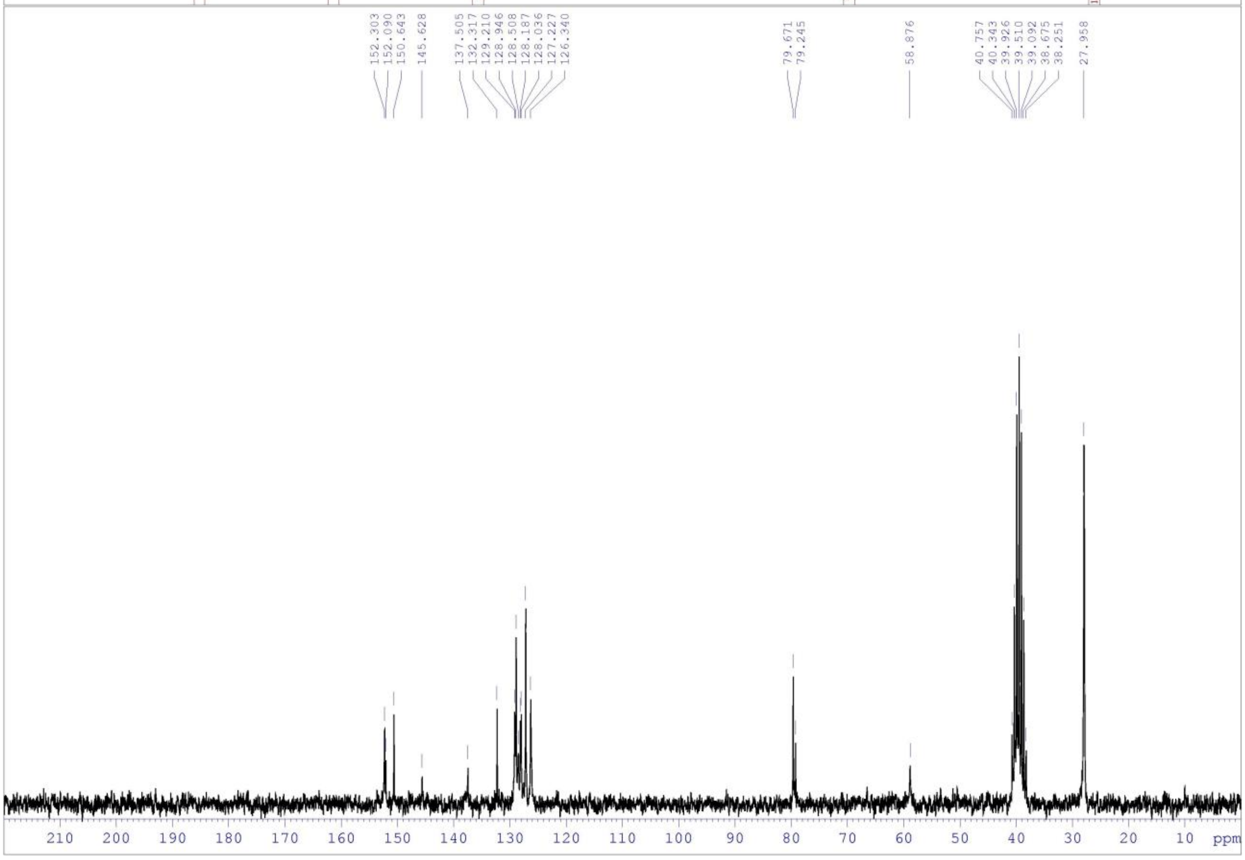
152.303
152.090
150.643
145.628
137.505
132.317
129.210
128.946
128.508
128.187
128.036
127.227
126.340
79.671
79.245
58.876
40.757
40.343
39.926
39.510
39.092
38.675
38.251
27.958
ppm

Compound **12f**

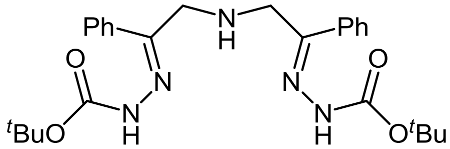

White foam, softening at. 99 °C, melting at 110 °C, *E*,*E*-**12f**.

^{1}H NMR (200 MHz, DMSO-d$_6$): δ = 1.39 (s, 18 H, 6 C*H_3* (^{t}Bu)), 3.49 (s, 4 H, 2 C*H_2)*, 6.67, 7.19 and 7.34 (3 m, 10 H, 2 *Ph*), 8.28 (s, 2 H, 2 N*H*),

^{13}C NMR (50 MHz, DMSO-d$_6$): δ = 27.9 (6 *C*H$_3$ (^{t}Bu)), 59.3 (2 *C*H$_2$), 79.7 (2 *C* (^{t}Bu)), 127.3, 128.6 and 132.7 (2 *Ph*), 150.7 and 152.1 (2 *C*=N and 2 *C*=O).

HRMS: Calcd for $C_{26}H_{35}N_5O_4$ [MNa$^+$] m/z: 504.2581. Found: 504.2569.

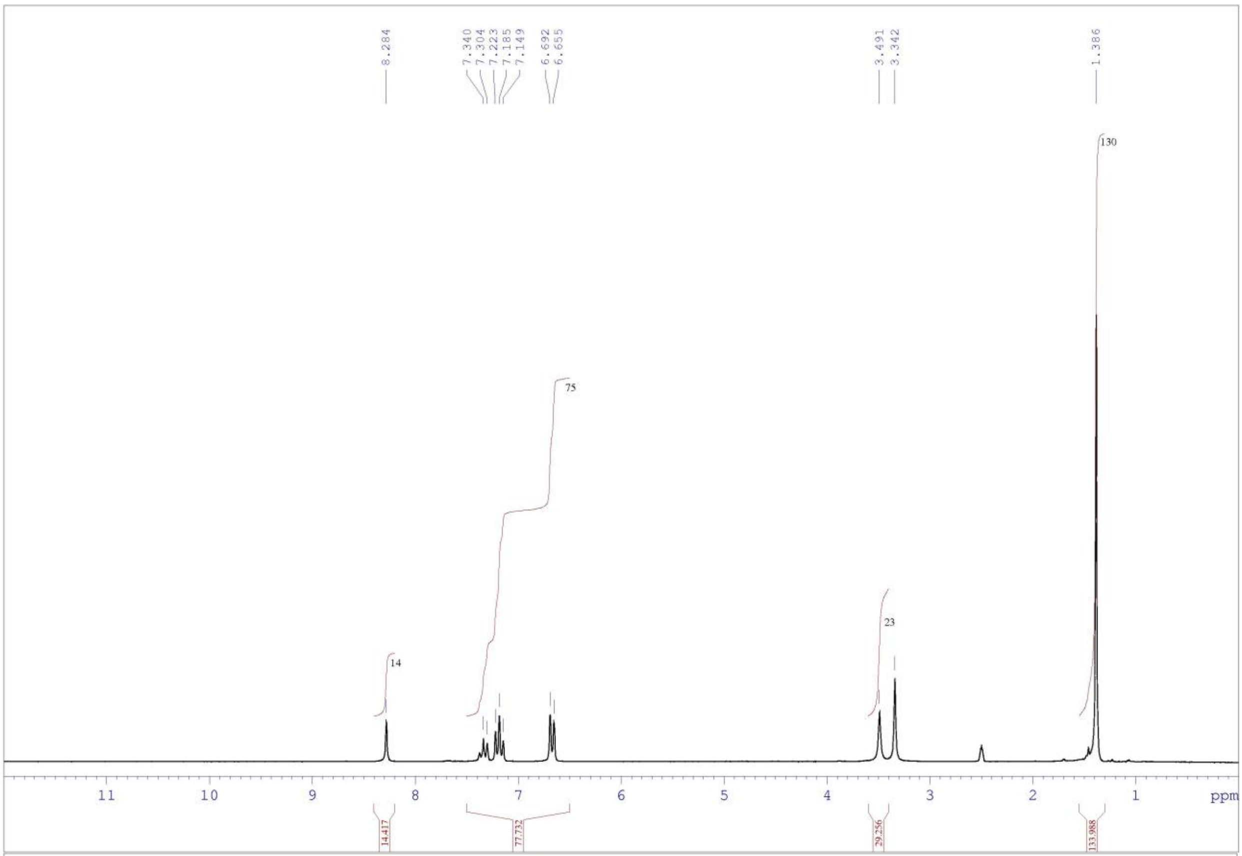
8.284
7.340
7.304
7.223
7.185
7.149
6.692
6.655
3.491
3.342
1.386
130
75
23
14
11
10
9
8
7
6
5
4
3
2
1
ppm
14.417
77.732
29.256
133.988

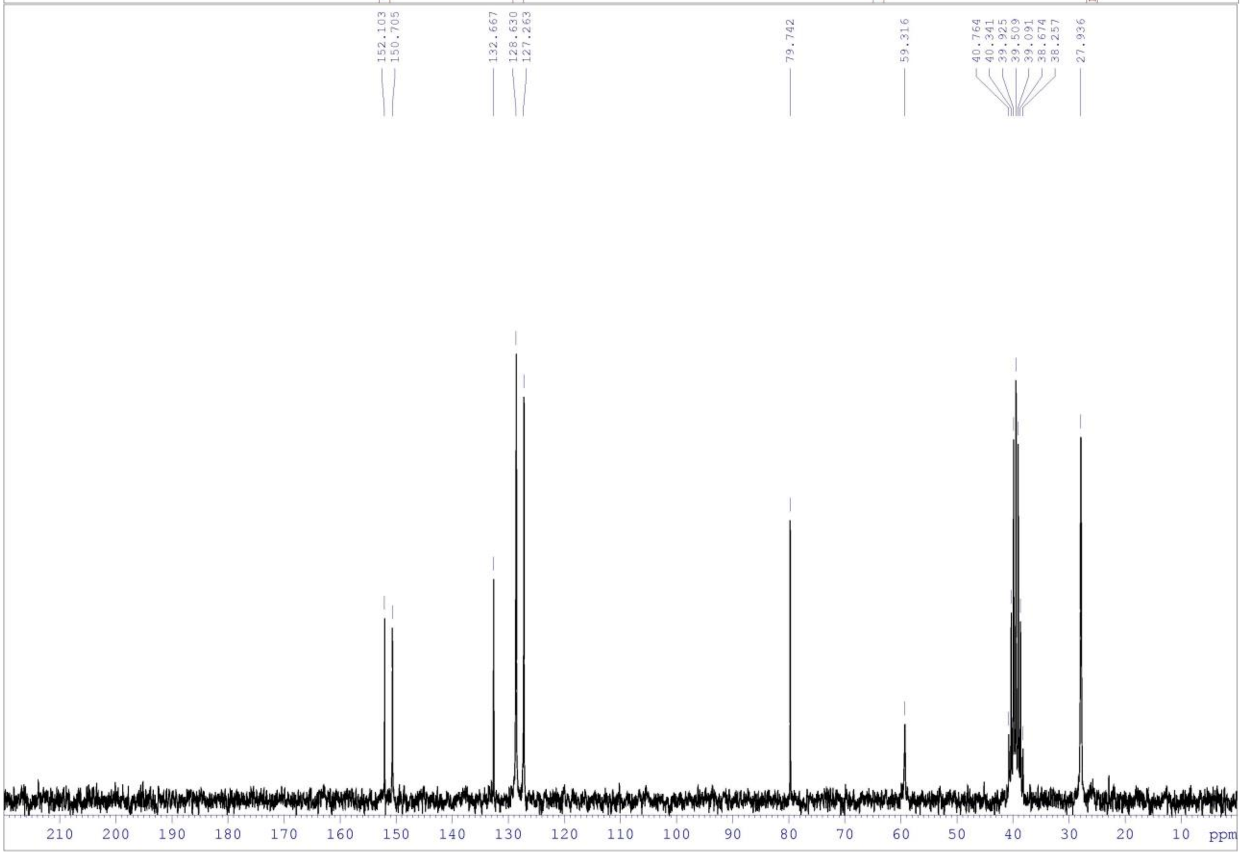
152.103
150.705
132.667
128.630
127.263
79.742
59.316
40.764
40.341
39.925
39.509
39.091
38.674
38.257
27.936
210
200
190
180
170
160
150
140
130
120
110
100
90
80
70
60
50
40
30
20
10
ppm

Compound **13b**

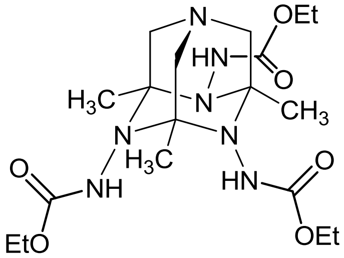

White solid, m.p. 135-138 °C. Mixture of conformers.

^{1}H NMR (200 MHz, DMSO-d$_6$): δ = 0.82 and 0.99 (2 s, 9 H, 3 C*H$_3$*), 1.17 (m, 9 H, 3 CH$_2$C*H$_3$*), 2.67, 2.96 and 3.30 (3 m, 6 H, 3 C*H$_2$*), 4.04 (m, 6 H, 3 C*H$_2$*CH$_3$), 7.34, 7.55, 8.21, 8.44 and 8.69 (5 br, 3 H, 3 N*H*).

^{13}C NMR (50 MHz, DMSO-d$_6$): δ = 14.6, 18.3 and 19.5 (6 *C*H$_3$), 46.2, 53.0, 54.6, 59.9 and 62.2 (6 *C*H$_2$), 72.9, 73.1 and 73.7 (3 *C*), 157.0 and 157.7 (3 *C*=O).

HRMS: Calcd for $C_{18}H_{34}N_7O_6$ [MH$^+$] m/z: 444.2565. Found: 444.2556.

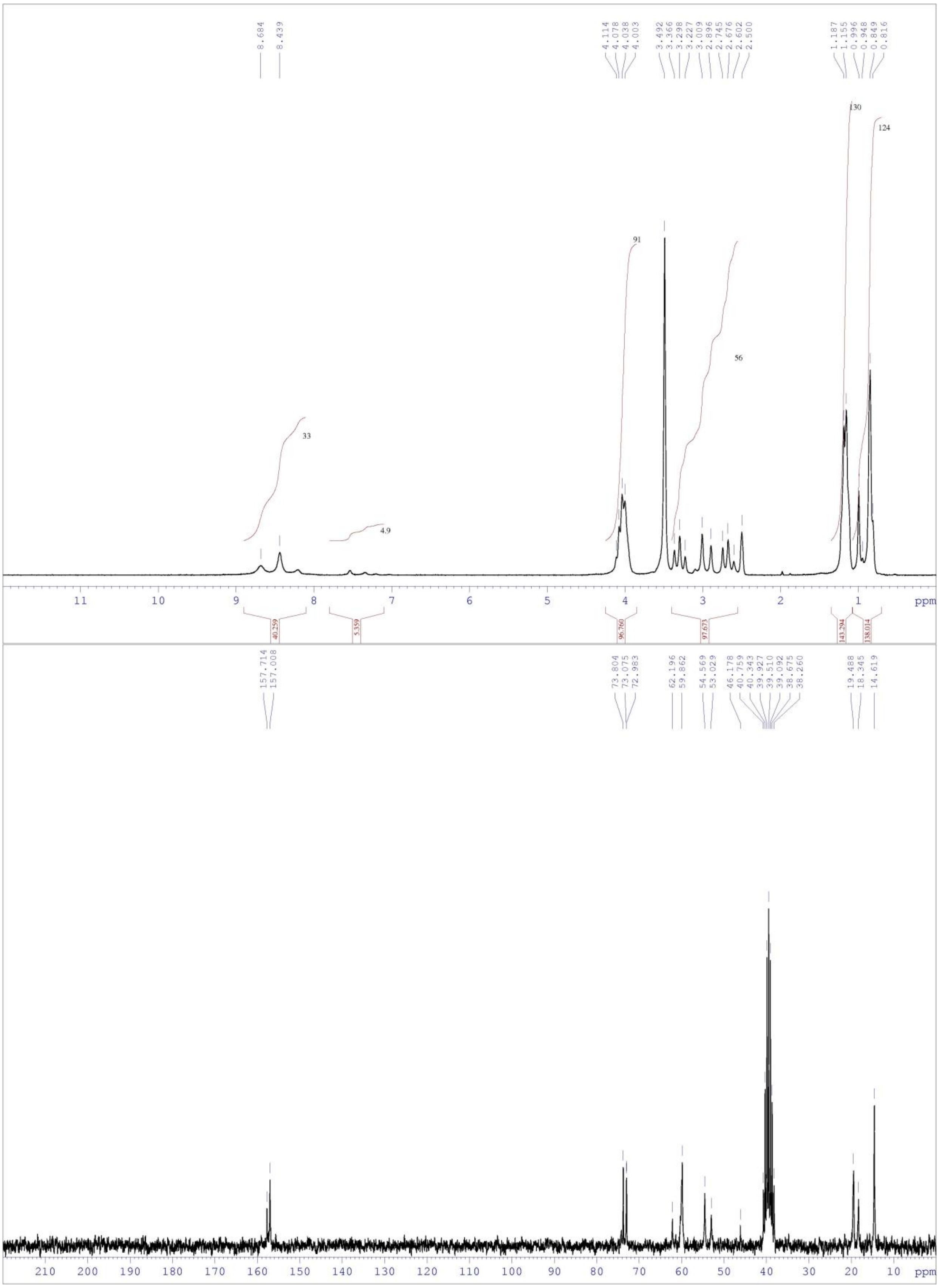

^{1}H NMR of **13b** at 330 K

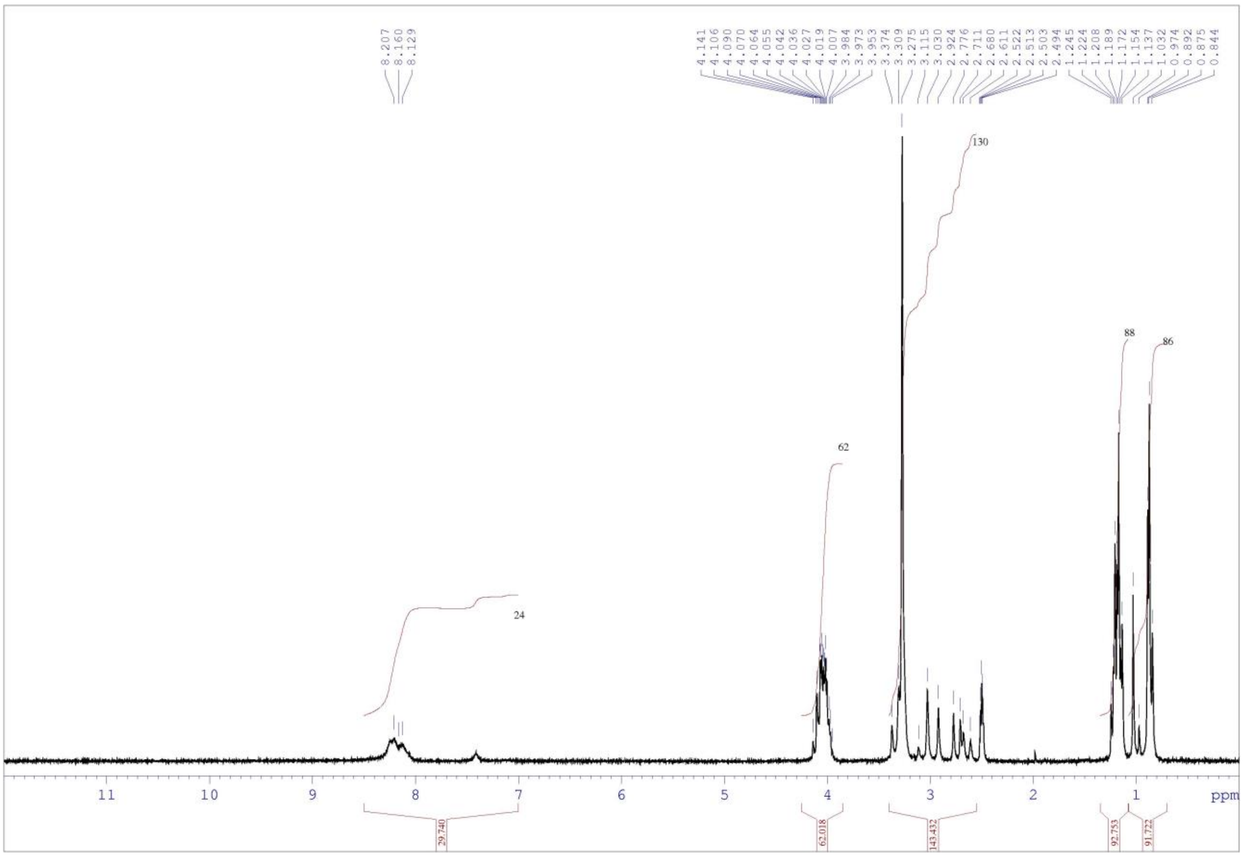

X-ray data for 2(13b)·2H_2O·MeOH

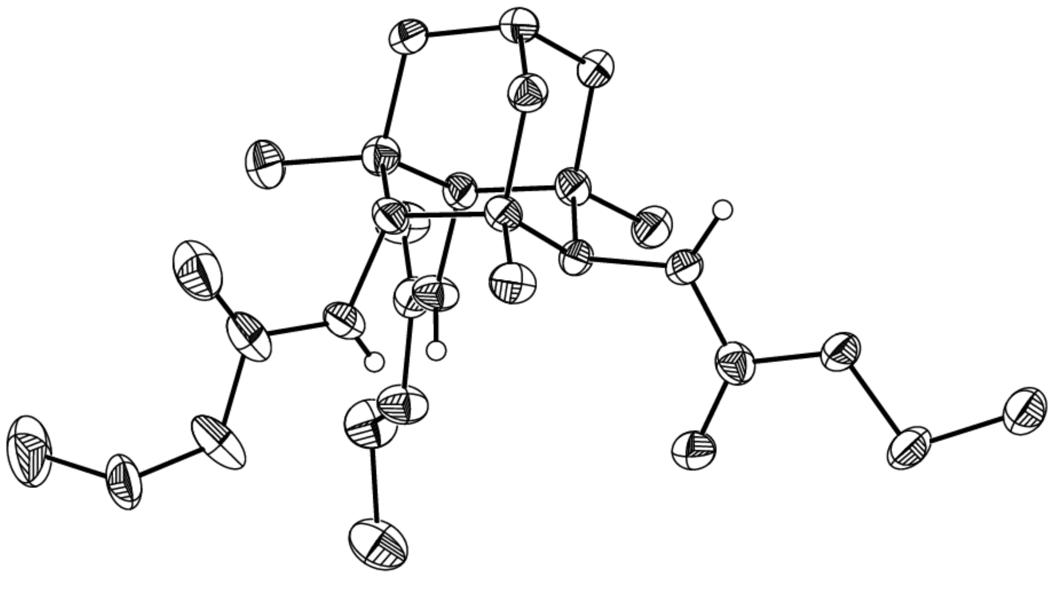

Figure S1: General view of **13b** in representation of atoms with thermal ellipsoids at 50% probability level; all hydrogen atoms (except for those of the NH groups) are omitted for clarity. The compound crystallizes as a crystallosolvate with two water and one methanol molecule (those are not shown) per two symmetry-independent molecules of the product.

Crystallographic data: Crystals of **13b** ($C_{37}H_{74}N_{14}O_{15}$, M = 955.10) are monoclinic, space group $P2_1/n$, at 100 K: a = 14.325(2), b = 12.234(2), c = 29.447(5) Å, β = 64.3830(10)°, V = 102.398(4) Å^3, Z = 4 (Z' = 2), d_{calc} = 1.259 g·cm^{-3}, μ(MoKα) = 0.98 cm^{-1}, F(000) = 2056. Intensities of 61716 reflections were measured with a Bruker APEX2 DUO CCD diffractometer [λ(MoKα) = 0.71072Å, ω-scans, 2θ<58°], and 13409 independent reflections [R_{int} = 0.2198] were used in the further refinement. The structure was solved by direct methods and refined by the full-matrix least-squares technique against F^2 in the anisotropic–isotropic approximation. The hydrogen atoms of the NH groups and those of water and methanol molecules were found in difference Fourier synthesis; the H(C) atom positions were calculated. All the hydrogen atoms were refined in the isotropic approximation within the riding model. The refinement converged to wR2 = 0.2712 and GOF = 1.054 for all the independent reflections (R1 = 0.0896 was calculated against F for 5215 observed reflections with I > 2σ(I))**.** All calculations were performed using SHELXTL PLUS 5.0.[5]

CCDC 1501437 contains the supplementary crystallographic data for **13b**. These data can be obtained free of charge via http://www.ccdc.cam.ac.uk/conts/retrieving.html (or from the CCDC, 12 Union Road, Cambridge, CB21EZ, UK; or deposit@ccdc.cam.ac.uk).

[5] Sheldrick, G. M. *Acta Cryst. A*, **2008**, *64*, 112-122
